# Quantitative Analysis of Proteome in Non-functional Pituitary Adenomas: Clinical Relevance and Potential Benefits for the Patients

**DOI:** 10.3389/fendo.2019.00854

**Published:** 2019-12-05

**Authors:** Tingting Cheng, Ya Wang, Miaolong Lu, Xiaohan Zhan, Tian Zhou, Biao Li, Xianquan Zhan

**Affiliations:** ^1^Key Laboratory of Cancer Proteomics of Chinese Ministry of Health, Xiangya Hospital, Central South University, Changsha, China; ^2^Hunan Engineering Laboratory for Structural Biology and Drug Design, Xiangya Hospital, Central South University, Changsha, China; ^3^State Local Joint Engineering Laboratory for Anticancer Drugs, Xiangya Hospital, Central South University, Changsha, China; ^4^Department of Oncology, Xiangya Hospital, Central South University, Changsha, China; ^5^National Clinical Research Center for Geriatric Disorders, Xiangya Hospital, Central South University, Changsha, China

**Keywords:** non-functional pituitary adenomas, quantitative proteomics, molecular network, Transcriptomics, Integrative analysis of proteomics and transcriptomics, signaling pathway, predictive preventive personalized medicine, biomarker pattern

## Abstract

**Background:** Non-functional pituitary adenoma (NFPA) is a common tumor that occurs in the pituitary gland, and generally without any symptoms at its early stage and without clinical elevation of hormones, which is commonly diagnosed when it grows up to compress its surrounding tissues and organs. Currently, the pathogenesis of NFPA has not been clarified yet. It is necessary to investigate molecular alterations in NFPA, and identify reliable biomarkers and drug therapeutic targets for effective treatments.

**Methods:** Tandem mass tags (TMT)-based quantitative proteomics was used to identify and quantify proteins in NFPAs. GO and KEGG enrichment analyses were used to analyze the identified proteins. Differentially expressed genes (DEGs) between NFPA and control tissues were obtained from GEO datasets. These two sets of protein and gene data were analyzed to obtain overlapped molecules (genes; proteins), followed by further GO and KEGG pathway analyses of these overlapped molecules, and molecular network analysis to obtain the hub molecules with Cytoscape. Two hub molecules (SRC and AKT1) were verified with Western blotting.

**Results:** Totally 6076 proteins in NFPA tissues were identified, and 3598 DEGs between NFPA and control tissues were identified from GEO database. Overlapping analysis of 6076 proteins and 3598 DEGs obtained 1088 overlapped molecules (DEGs; proteins). KEGG pathway analysis of 6076 proteins obtained 114 statistically significant pathways, including endocytosis, and spliceosome signaling pathways. KEGG pathway analysis of 1088 overlapped molecules obtained 52 statistically significant pathways, including focal adhesion, cGMP-PKG pathway, and platelet activation signaling pathways. These pathways play important roles in cell energy supply, adhesion, and maintenance of the tumor microenvironment. According to the association degree in Cytoscape, ten hub molecules (DEGs; proteins) were identified, including GAPDH, ALB, ACACA, SRC, ENO2, CALM1, POTEE, HSPA8, DECR1, and AKT1. Western-blotting analysis confirmed the upregulated expressions of SRC and PTMScan experiment confirmed the increased levels of pAKT1, in NFPAs compared to controls.

**Conclusions:** This study established the large-scale quantitative protein profiling of NFPA tissue proteome. It offers a basis for subsequent in-depth proteomics analysis of NFPAs, and insight into the molecular mechanism of NFPAs. It also provided the basic data to discover reliable biomarkers and therapeutic targets for NFPA patients.

## Introduction

Pituitary adenoma is an intracranial tumor, which is clinically classified as functional pituitary adenomas (FPAs) with the elevation of the corresponding hormone in blood and non-functional pituitary adenomas (NFPAs) without clinical elevation of hormones in the blood. NFPA has the prevalence of 7-22/100,000 (1, 2), the standardized incidence of 1.02/100,000 (3), and accounts for 15–37% of all pituitary adenomas, with a median age range from 51.5 to 65.5 years (1–3). Because NFPA does not have excessive hormone secretion, it is not easily diagnosed at its early stage, but is often recognized only when it grows up to compress its surrounding tissues and organs; and commonly 67–90% of diagnosed NFPAs have reached up to a quite large volume (1–3). The natural course of NFPA has not been fully understood. Most NFPAs are benign, only a very small number of NFPAs has the characteristics of invasiveness, aggressiveness, or malignancy. Conversely, the tumor volume of some NFPA patients is also gradually decreasing during its pathological process (4, 5). Currently, no early-stage-diagnosis biomarkers are used for NFPAs, and the classic oncogene mutations that occur in other tumors are not also found in NFPAs. However, studies show that the disrupted cell cycle control and growth factor signaling likely contribute to pathogenesis and natural history of NFPAs (6).

NFPA has a very complex pathogenesis process, involves multiple molecular systems (7), and has heterogeneity in origin of cells and in hormone expressions (8, 9). A study shows that Ki-67 is involved in the growth and reproduction of NFPA, just like other tumors, which is the most consistent marker to assess biological behavior in NFPAs (10). One proteomics analysis of 34 sera from NFPA patients and healthy controls with matched age and sex factors identified nine serum differentially expressed proteins (DEPs) (7 up-regulated and 2 down-regulated), which was able to discriminate NFPAs from normal controls with a good sensitivity (82.4%) and specificity (82.4%) (11). A two-dimensional gel electrophoresis (2DGE)-based mapping proteomic analysis of human FSH-positive NFPA tissues detected ~1,200 protein spots, which identified 192 redundant proteins from 141 spots and revealed several important pathway-network changes (cell cycle dysregulation, oxidative stress, mitochondrial dysfunction, and MAPK signaling abnormality) (12). However, this 2DGE mapping proteomic study has a relative narrow throughput in identification of proteins, and does not get quantitative information of proteins. Therefore, it is necessary to obtain a high-throughput and large-scale proteomic profile for in-depth understanding of molecular mechanisms and discovery of effective biomarkers for NFPAs. Mass spectrometry (MS) is an essential technique to identify and quantify proteins and post-translational modifications (PTMs) in a proteome (12). 2DGE or two-dimensional difference in-gel electrophoresis (2D DIGE) coupled with MS was extensively used to study pituitary adenomas (8, 13). However, the previous 2DGE-based proteomics in human pituitary adenomas usually achieved the relative low throughput (dozens to several hundreds) in identification of proteins due to the conventional concept of 2DGE (14, 15). Tandem mass tags (TMT)-based two-dimensional liquid chromatography-tandem mass spectrometry (2DLC-MS/MS) can easily achieve several thousands of proteins to significantly increase the throughput in identification of proteins for more effectively mining proteomic components, which is an effective peptide-based protein identification method (13). Moreover, studies have demonstrated that non-coding RNAs, including microRNAs (miRNAs) and long non-coding RNAs (lncRNAs), are involved in the development of NFPAs (16). For example, miR-145-5p in NFPA samples is significantly reduced and negatively correlated with NFPA invasiveness, and overexpression of miR-145-5p can inhibit proliferation and invasiveness of NFPA cells and promote apoptosis (17). Another study shows that CXCR4 mRNA is expressed in 92% of growth hormone secretory pituitary adenomas (GHoma) and 81% NFPAs, whereas SDF1 is found in 63% of GHomas and 78% of NFPAs; and CXCR4 and SDF1 are the strong homogenous markers in all tumor cells of GHomas and NFPAs (18). Therefore, integrative analysis of protomics and transcriptomics has significantly scientific merit for NFPAs (19, 20).

This study used TMT-labeled 2DLC–MS/MS to identify and quantify protein expression profiles of NFPAs. The identified protein data were compared to differentially expressed genes (DEGs) that were obtained from the Gene Expression Omnibus (GEO) database to obtain the overlapped molecules (DEGs; proteins). The overlapped molecules were analyzed with gene ontology (GO) enrichment, KyotoEncyclopaedia Gene and Genome (KEGG) pathway, and protein-protein interactions. The hub molecules (DEGs; proteins) were obtained from KEGG pathway networks, and verified with Western blotting analysis. The resulting data established the large-scale database for NFPA proteome, and provided the scientific data to in-depth understand molecular mechanisms of NFPAs, and discover reliable biomarkers for NFPA treatment.

## Materials and Methods

### NFPA and Control Pituitary Tissues

The post-mortem control pituitary tissue samples used for Western blotting analysis were obtained from Memphis Regional Medical Center, with an approval of University of Tennessee Health Science Center Internal Review Board (UTHSC-IRB). The NFPA tissue samples used for proteomics and Western blotting analyses were obtained from Department of Neurosurgery, Xiangya Hospital, Central South University, with an approval of the Medical Ethics Committee of Xiangya Hospital of Central South University. The written consent information was obtained from the family of each control pituitary donor or each patient after the purpose and nature of all used procedures were fully explained. The detailed information was shown for these NFPA and control pituitary tissue samples (Table 1).

**Table 1:** Clinical characteristics of NFPA and control tissue samples.

**Group**	**Sex**	**Age (year)**	**Immunohistochemistry (IHC) or clinical information**	**Experiments**	
NFPAs	Male	49	ACTH(–), hGH(–), PRL(–), FSH(–), LH(–), TSH(–)	Proteomics	
	Female	53	ACTH(–), hGH(–), PRL(–), FSH(–), LH(–), TSH(–)	Proteomics	
	Male	40	ACTH(–), hGH(–), PRL(–), FSH(+), LH(–), TSH(–)	Proteomics	
	Male	52	ACTH(–), hGH(–), PRL(–), FSH(+), LH(–), TSH(–)	Proteomics	
	Female	43	ACTH(–), hGH(–), PRL(–), FSH(+), LH(–), TSH(–)	Proteomics;	Western blot
	Male	58	ACTH(–), hGH(–), PRL(–), FSH(–), LH(–), TSH(–)	Proteomics;	Western blot
	Female	44	ACTH(–), hGH(–), PRL(–), FSH(+), LH(–), TSH(–)		Western blot
	Male	53	ACTH(–), hGH(–), PRL(–), FSH(–), LH(–), TSH(–)		Western blot
Controls	Female	40	White, Multiple toxic compounds. Blood: HepB (+), HepC (+), HIV(–). IHC: do not test.		Western blot
	Male	36	White, Multiple toxic materials. Blood alcohol = 0.5 g/L. Blood: HepB (+), HepC (–), HIV (–). IHC: do not test.		Western blot
	Female	34	Black, Gunshot wound to chest. Blood alcohol = 0.3 g/L; no drugs. Blood: HepB (+), HepC (–), HIV (–). IHC: do not test.		Western blot
	Female	/	White, 15 h gunshot wound to head. No drugs or alcohol. Blood: HepB (–), HepC (–), HIV (–). IHC: do not test.		Western blot

### Protein Extraction

Each sample was grinded with liquid nitrogen. The grinded samples were collected into a 5-mL centrifuge tube, and a volume of lysis buffer was added that contained 8 M urea, 10 mM dithiothreitol (DTT), 2 mM ethylene diamine tetraacetic acid (EDTA), and 1% protease inhibitor cocktail III, followed by sonication (3x; ice), and centrifugation (20,000 g, 4°C, 10 min). The proteins in the supernatant were precipitated (2 h; −20°C) with a volume of 15% trichloroacetic acid (TCA), and centrifuged (4°C, 10 min) to discard the supernatant. The proteins in precipitate were washed with cold acetone (3x), and then redissolved in a volume of buffer that contained 8 M urea, and 150 μl 100 mM tetraethylammonium bromide (TEAB) at pH 8.0. The 2-D Quant kit was used to determine the protein concentration.

### Trypsin Digestion

A volume of reducing solution including 10 mM DTT was added to the protein samples, incubated (37°C; 2 h) in water bath, and cooled at room temperature, followed by a quick addition of alkylating reagent (20 mM iodoacetamide) and incubation in the dark (room temperature; 45 min). The solution of 100 mM TEAB was added to each protein sample to dilute urea concentration to 2M or less. Finally, an amount of trypisn (trypsin/protein mass ratio = 1:50) was added to each tube for overnight hydrolysis, and then added trypsin (trypsin/protein mass ratio = 1: 100) to each tube for another 4 h digestion. The tryptic peptides (100 μg) of each sample were used for subsequent experiments.

### TMT Labeling

Each tryptic peptide sample was desalted with Strata X C18 solid-phase extraction column (Phenomenex), and vacuum dried. Each desalted tryptic peptide sample was dissolved in the solution of 0.5 M TEAB, and labeled with 6-plex TMT reagent according to the manufacturer's procedure with a ratio of 1 unit of TMT reagent to 100 μg of tryptic peptides. Briefly, the tryptic peptide sample was incubated (room temperature; 2 h) with TMT reagent, mixed together (1:1), desalted, and lyophilized by vacuum centrifugation. Parallel replicates of the peptide fragments of two groups were performed to eliminate errors due to external confounding factors such as experimental procedures and instrument.

### HPLC Fractionation

The desalted TMT-labeled tryptic peptide mixture was fractionated into 80 fractions over 80 min with high-pH reverse-phase high-performance liquid chromatography (HPLC). The Agilent 300 Extend C18 column (5 μm particles, 4.6 mm ID, and 250 mm length) was used to separate peptides with a 2–60% acetonitrile (ACN) plus 10% ammonium bicarbonate at pH 10. Finally, 80 fractions were grouped into 18 fractionated samples, and vacuum dried.

### LC-MS/MS

For 18 fractionated peptide samples, each fractionated peptide sample was added with a volume of 0.1% trifluoroacetic acid (TFA), mixed well, and loaded onto the Acclaim PepMap 100 reverse-phase precolumn (Thermo Scientific) to online enter the Acclaim PepMap RSLC reverse-phase analytical column (Thermo Scientific). The LC gradient was set as a 5–25% increase of solvent B (0.1% TFA plus 98% ACN) over 60 min, a 25–35% increase of solvent B in 12 min, and an increase to 80% of solvent B in 4 min, then remained in 80% of solvent B for the last 4 min, with a constant flow-rate (320 nl/min) on an EASY-nLC 1000 UPLC system. MS/MS spectra were obtained on an OrbiTrap Fusion^TM^ MS instrument (ThermoFisher Scientific). Its detection resolution was set as 70,000 for precursor ions in the MS spectrum. Product ion information in the MS/MS spectrum was obtained with high energy collision dissociation (HCD) cleavage for fragmentation of precursor ion, with the collision energy of 38. The resolution of product ions was set as 15,000. The electrospray voltage was set as 2.0 kV. With the automatic gain control (AGC) function, the pre-scan before each sample scan automatically balanced the number of ion implants to prevent charge overload in the analyzer. Cumulative 5E4 intensity ions in the MS spectra were analyzed for MS/MS. The primary MS scan range was set as *m/z* 400–1,600, the starting point of the secondary MS scan range was fixed at *m/z* 100.

### Database Search of MS/MS Data and Functional Characteristics of Identified Proteins

Mascot search engine (v.2.3.0) was used to search proteins with MS/MS data against UniProt human database (https://www.uniprot.org). UniProt is the most informative and resource-rich protein database. Its data are mainly the subsequent protein sequences, which are derived from the completion of the genome sequencing. It contains a wealth of information on the biological functions of proteins from the literature. The R-software cluster profile was used to reveal gene ontology (GO) characteristics of identified proteins: cellular components (CCs), biological processes (BPs), and molecular functions (MFs). KEGG pathway enrichments were performed for the identified proteins. Benjamini-Hochberg-based adjusted *p* < 0.05 was used as statistical significance. PANTHER (http://www.pantherdb.org/) and Cytoscape software were also used to enrich CCs.

### GEO Gene Data of NFPAs

The GEO database is a high-throughput gene expression database submitted by research institutions around the world, which is created in 2000 and maintained by the National Center for Biotechnology Information (NCBI). This study obtained microarray gene data GSE51618 profile datasets of human pituitary adenomas from the public GEO database (http://www.ncbi.nlm.nih.gov/geo/), which were derived from the analysis of 11 tissue samples (3 control pituitaries, 4 non-invasive NFPAs, and 4 invasive NFPAs) with a gene chip human genome platform (Agilent-014850 Whole Human Genome Microarray 4x44K G4112F) in other laboratory. The R-software was used to analyze these NFPA vs. control GEO gene data. False discovery rate (FDR) < 0.05 and fold-changes (FC) ≥ 2 were used to determine each DEG. DEGs were obtained between non-invasive NFPAs and controls, and between invasive NFPAs and controls. Because non-invasive and invasive NFPAs were all NFPAs, thus two sets of DEG data were combined to become one set of DEG data between NFPA and control tissues, which were overlapped with the identified proteins in NFPAs.

### Overlapping Analysis of Protein Data and DEG Data

The gene name corresponding to each identified protein was obtained in UniProt human database. Thus, overlapping analysis was performed between the gene names of identified proteins in NFPAs and DEG data between NFPA and control tissues, to obtain the overlapped molecules (DEGs; proteins) for further bioinformatics and functional analysis.

### GO and KEGG Pathway Enrichments of Overlapped Molecules

The Database for Annotation, Visualization, and Integrated Discovery (DAVID) provides the comprehensive functional annotation tools for investigators to understand biological meaning behind a large list of genes. DAVID-based GO and KEGG pathway enrichments were used to analyze those overlapped molecules (DEGs; proteins). The parameters (*p* < 0.05 and gene count > 5) were considered as statistical significance. Furthermore, each *p*-value was corrected with FDR for multiple testing.

### Prediction of Protein–Protein Interaction

STRING 10.0 (http://string-db.org/cgi/input.pl) was used to construct the protein-protein interaction (PPI) network of those overlapped molecules (DEGs; proteins) with a high confidence (>0.700). Then Cytoscape software (3.6.1) was used to get hub molecules (genes; proteins) based on degrees (Pearson's correlation coefficient >0.50, *P* < 0.05).

### Western Blotting

The 10% sodium dodecyl sulfate-polyacrylamide gel electrophoresis (SDS-PAGE) gel was used to separate proteins (NFPAs; controls). The separated proteins were transferred onto a polyvinylidene fluoride (PVDF) membrane. The proteins on PVDF membrane were incubated (4°C; overnight) with mouse anti-human SRC antibody (1:1000), AKT1 antibody (1:1000), and β-actin antibody (1:2000), followed by incubation (2 h; room temperature) with secondary antibody (horseradish peroxidase-conjugated goat anti-mouse antibody; 1:5000). The Western blotting experiments of each protein between NFPAs and controls were repeated (≥3). Student's *t*-test was used to calculate the *p*-value, with a statistical significance level of *p* < 0.05.

## Results

### Proteomic Profiling of NFPAs and Its Functional Characteristics

TMT-based quantitative proteomics identified 6076 proteins in NFPAs (Supplemental Table 1), including 4666 proteins with quantitative information. Peptide sequence match (PSM) was set as ≥ 1 for identification of each protein. The analysis of 6076 proteins revealed that more than 94% of identified proteins were distributed in the range of 7–200 kDa and pH 4–10. The top 11 abundance proteins (Supplemental Table 1) were SNRPB (small nuclear ribonucleoprotein-associated proteins B), TECR (very-long-chain enoyl-CoA reductase), TMEM263 (transmembrane protein 263), MARC1 (mitochondrial amidoxime-reducing component 1), APMAP (adipocyte plasma membrane-associated protein), CSNK1A1 (casein kinase I isoform alpha), APOC3 (apolipoprotein C-III), CSTB (cystatin-B), TTR (transthyretin), IGHA1 (immunoglobulin heavy constant alpha 1), and GET4 (Golgi to ER traffic protein 4 homolog). However, it is worth noting that many lower abundance proteins might play more important roles in the molecular networks (12).

**Figure 1:**
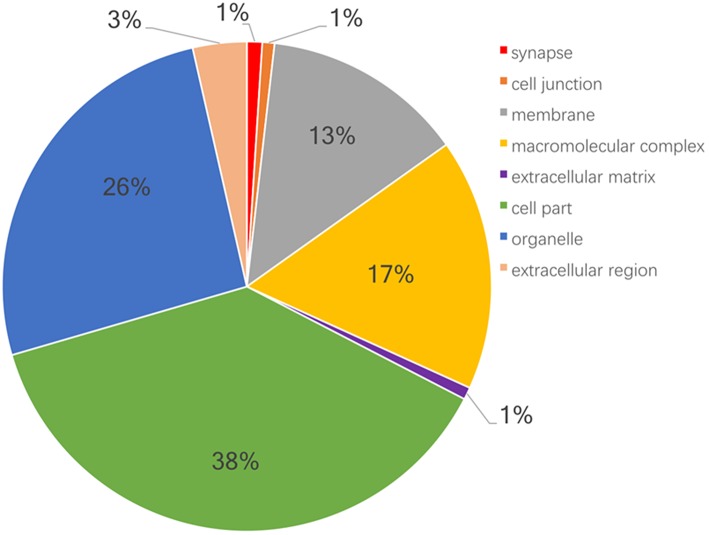
Classification of 6076 proteins according to the cell components with PANTHER.

**Figure 2:**
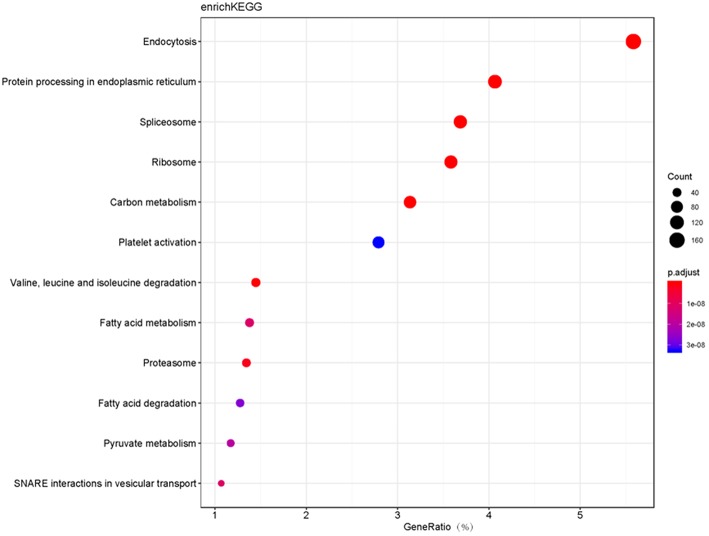
The bubble plot of top 12 KEGG pathway enrichments with 6076 proteins (*P* < 0.05). The bubble color scaled the enrichment score. Red color means more significant enrichment. The size of the bubble scaled the count of the enriched genes. X-axis is equal to gene ratio, which means the percentage of enriched target genes among total 6076 genes. Y-axis is the name of the KEGG pathway.

Moreover, GO and KEGG pathway enrichment analyses were used to reveal the potential functions of those 6076 proteins. GO-based CC, BP, and MF enrichment analyses revealed the overall functional characteristics of 6076 proteins. Those proteins were mainly distributed in cell part (38 %), organelle (26 %), macromolecular complex (17 %), and membrane (13%) (Figure 1). KEGG pathway enrichment analysis (*P* < 0.05) of 6076 proteins identified 114 statistically significant signaling pathways (Supplemental Table 2). Based on the number of matched proteins in each pathway and the *p*-value, 12 important pathways were identified (Figure 2), including endocytosis, protein processing in endoplasmic reticulum, spliceosome, ribosome, carbon metabolism, platelet activation, valine, leucine and isoleucine degradation, fatty acid metabolism, proteasome, fatty acid degradation, pyruvate metabolism, and SNARE interactions in vesicular transport. These pathways were involved in cellular energy metabolism, protein synthesis and processing, which played important roles in tumorigenesis and progression.

### DEG Profiling in NFPAs and Its Functional Characteristics

**Figure 3:**
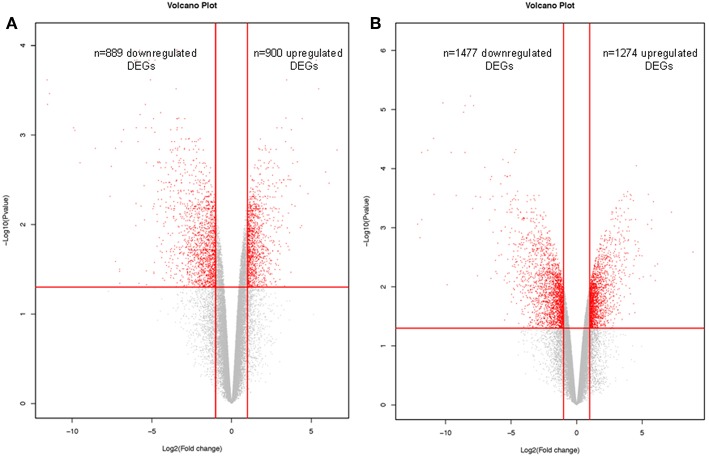
Volcano plots of differentially expressed genes (DEGs) that were derived from microarray analysis of non-invasive NFPAs **(A)** and invasive NFPAs **(B)** compared to control tissues. The native log_10_ [false discovery rate (FDR) adjusted *p*-values] in the y-axis was plotted against the average log_2_(fold changes in expression) in the x axis. DEGs were determined using Limma followed by FDR correction. Horizontal dashed line indicated the threshold for significance (FDR adjusted *p* < 0.05) and vertical dashed line indicated the upregulated (right side) and downregulated (left side) probes.

Microarray transcritpomic data of NFPAs were obtained from GEO database. A total of 1789 DEGs was obtained between non-invasive NFPAs and controls, including 900 (50.31%) upregulated and 889 (49.69%) downregulated DEGs (Figure 3A, Supplemental Table 3). A total of 2751 DEGs was identified between invasive NFPAs and controls, including 1274 (46.31%) upregulated and 1477 (53.69%) downregulated DEGs (Figure 3B, Supplemental Table 4). Because NFPAs include invasive and non-invasive NFPAs, these two sets of DEG data (*n* = 1,798 in non-invasive NFPAs; and *n* = 2,751 in invasive NFPAs) were combined into one set of DEG data (*n* = 3,598) between NFPAs and controls after removal of the repeated DEGs between invasive and non-invasive NFPAs, including 1761 upregulated DEGs, 1764 downregulated DEGs, and 73 genes that were inconsistent in two sets (non-invasive vs. invasive) of DEG data (Supplemental Table 5).

### Overlapped Molecules Between 3598 DEGs and 6076 Proteins, and Their Functional Characteristics

**Figure 4:**
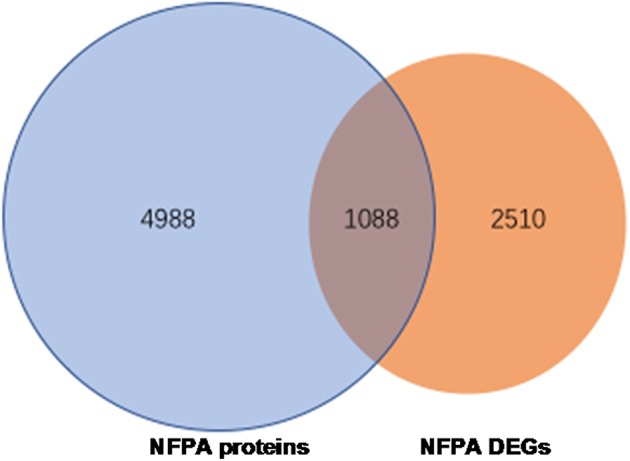
The 1088 overlapped molecules (DEGs; proteins) between 6076 identified proteins and 3598 DEGs obtained from GEO database.

Overlapped molecules (DEGs; proteins): An overlapping analysis was performed between 3598 DEG data and 6076 proteins, which obtained 1088 overlapped molecules (DEGs; proteins) (Figure 4), including 644 upregulated DEGs, 426 downregulated DEGs, and 18 genes that were inconsistent in two sets (non-invasive vs. invasive) of DEG data (Supplemental Table 6).

#### GO Enrichment Analysis

**Figure 5:**
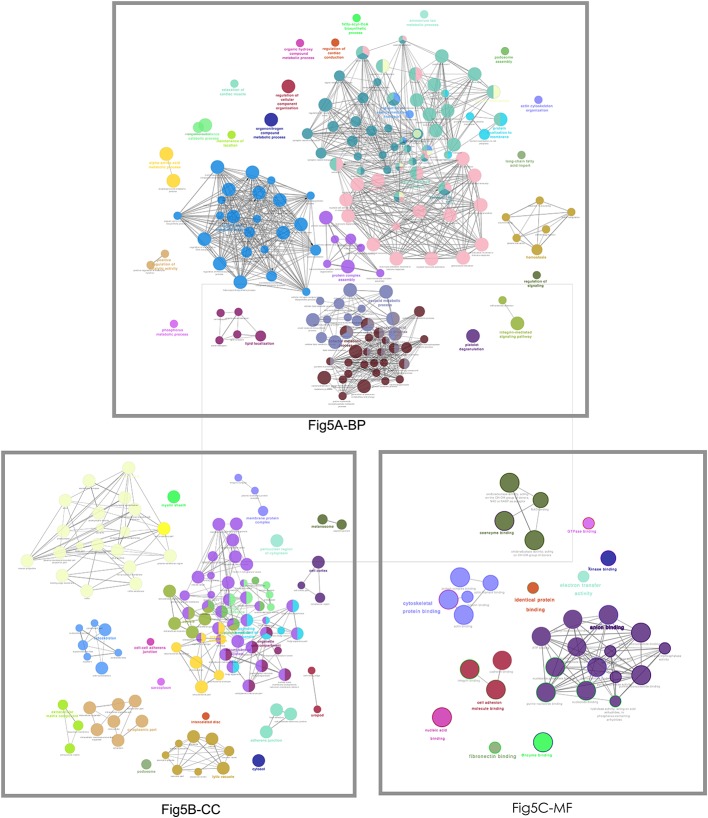
The functional characteristics of 1088 overlapped molecules (DEGs; proteins) according to the biological process (BP), cellular component (CC), and molecular function (MF). The less p-value and more significant enrichments were shown with the greater node size. The same color indicated the same function group. Among the groups, a representative of the most significant term and lag highlighted was chosen. The larger node means less p-values and more significant enrichments. The same color represents the same functional group.

Those 1088 overlapped molecules (DEGs; proteins) were grouped according to BP, CC, and MF with Cytoscape software (Figure 5). For BP enrichment, the overlapped molecules (DEGs; proteins) were mainly enriched in neutrophil degranulation, neutrophil activation, neutrophil-mediated immunity, regulation of vesicle-mediated transport, small molecule catabolic process, signal release, organic hydroxy compound metabolic process, coenzyme metabolic process, organic acid biosynthetic process, and carboxylic acid biosynthetic process. For CC enrichment, the overlapped molecules (DEGs; proteins) were mainly enriched in cell-substrate junction, focal adhesion, cell-substrate adhesion junction, myelin sheath, cytoplasmic vesicle lumen, vesicle lumen, secretory granule lumen, ruffle membrane, postsynapse, and ruffle. For MF enrichment, the overlapped molecules (DEGs; proteins) were mainly involved in cell adhesion molecule binding, actin binding, cadherin binding, tubulin binding, coenzyme binding, guanyl ribonucleotide binding, guanyl nucleotide binding, ATPase activity coupled, and microtubule binding.

#### KEGG Pathways

**Figure 6:**
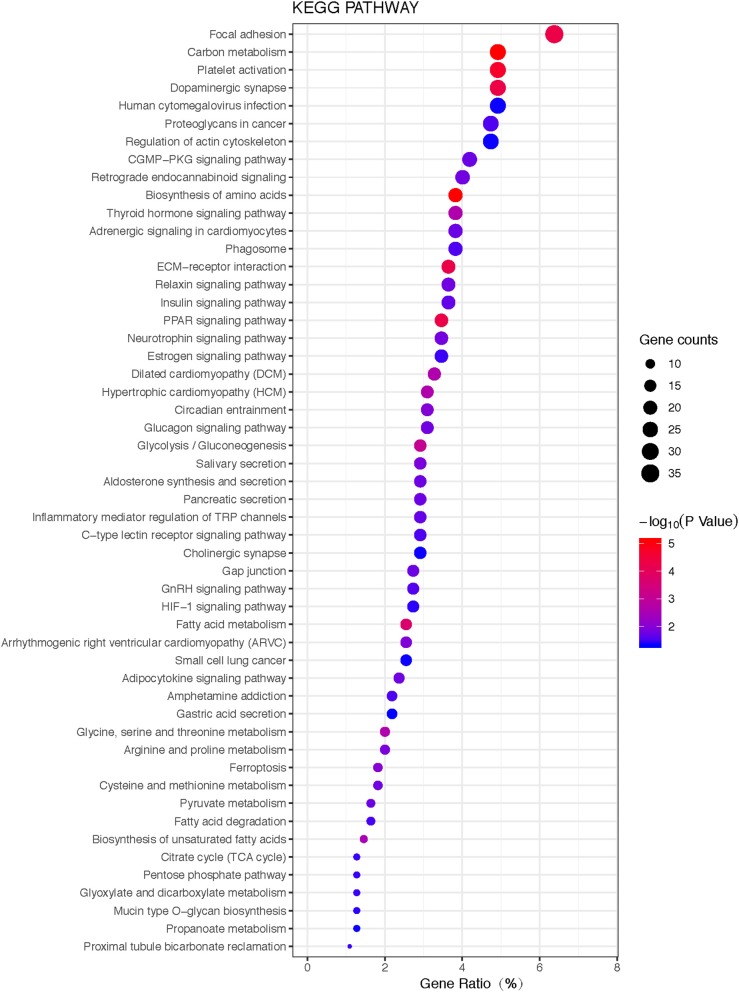
The bubble plot of KEGG pathway enrichments of 1088 overlapped molecules (DEGs; proteins) (*P* < 0.05). The bubble color scaled the enrichment score. Red color means more significant enrichment. The size of the bubble scaled the count of the enriched genes. X-axis is equal to gene ratio, which means the percentage of enriched target genes among total 1088 molecules (DEGs; proteins). Y-axis is the name of the KEGG pathway.

KEGG pathway analysis of those 1088 overlapped molecules (DEGs; proteins) revealed 52 statistically significant pathways (adjusted *p*-value < 0.05, count>5) (Figure 6; Supplemental Figure 1). The overlapped molecules (DEGs; proteins) were mainly enriched in the following pathways: focal adhesion, cGMP-PKG signaling pathway, platelet activation, carbon metabolism, dopaminergic synapse, human cytomegalovirus infection, proteoglycans in cancer, regulation of actin cytoskeleton, retrograde endocannabinoid signaling, and biosynthesis of amino acids.

#### Construction of PPI Network to Select Hub Molecules

**Figure 7:**
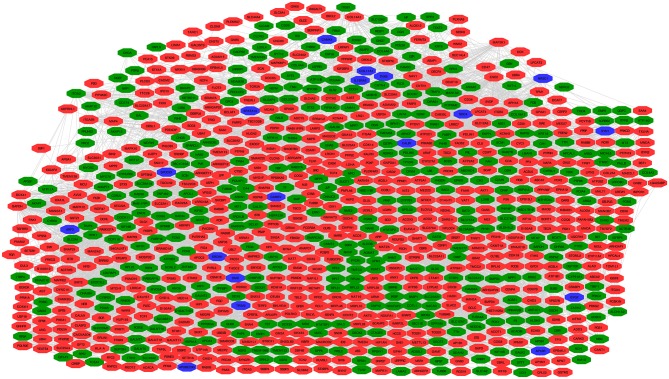
Protein-protein interaction (PPI) network of 1088 overlapped molecules (DEGs; proteins). Pearson's correlation coefficient >0.50, and *P* < 0.05. Green indicates the downregulated genes, red indicates the upregulated genes, and blue indicates genes that are inconsistent in invasive or non-invasive NFPAs.

**Figure 8:**
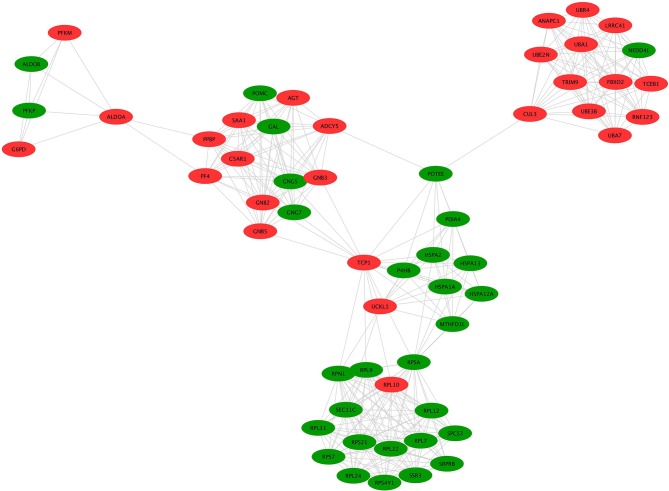
MCODE identification of the most significantly enriched module. The module with the highest MCODE score was selected from the PPI network. Green indicates the downregulated genes and red indicates the upregulated genes.

A hub molecule is a molecule that plays a vital role in biological processes, and regulates other molecules in a pathway network. The PPI network of 1088 overlapped molecules (DEGs; proteins) was constructed and the most significant module was obtained with Cytoscape (Figure 7). According to degree levels, the top 10 hub molecules (nodes: DEGs; proteins) were glyceraldehyde-3-phosphate dehydrogenase (GAPDH; degree = 148), serum albumin (ALB; degree = 143), acetyl-CoA carboxylase 1 (ACACA; degree = 137), proto-oncogene tyrosine-protein kinase (SRC; degree = 115), RAC-alpha serine/threonine-protein kinase (AKT1; degree = 114), calmodulin (CALM1; degree = 109), POTE ankyrin domain family member E (POTEE; degree = 108), heat shock cognate 71 kDa protein (HSPA8; degree = 92), mitochondrial 2,4-dienoyl-CoA reductase (DECR1; degree = 82), and gamma-enolase (ENO2; degree = 75). The degree of the vertice is the most basic structure of the graph, which refers to the number of edges associated with it. Molecular Complex Detection (MCODE) detects the densely connected regions in large protein-protein interaction networks that may represent molecular complexes (21). A significant module was subsequently constructed with 57 nodes and 347 edges, which gained the highest MCODE score (Figure 8).

#### Western Blotting Validation of Overlapped Molecules (DEGs; Proteins)

**Figure 9:**
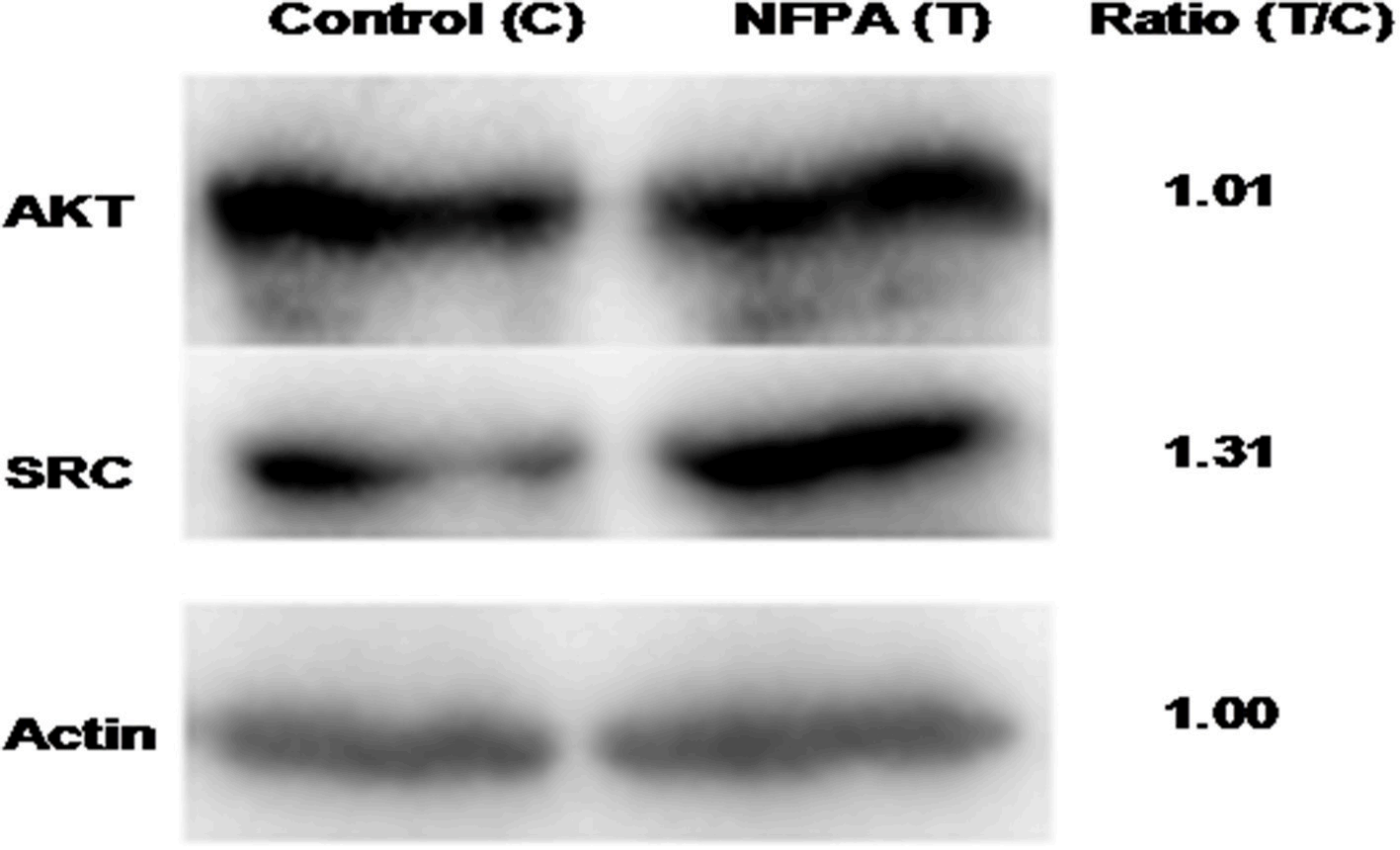
Western blotting analysis of overlapped molecules (AKT and SRC) between NFPAs and controls. T, tumor (NFPAs); C, control (pituitary tissues). Ratio (T/C) means the ratio of the optic density (OD) values between NFPAs and controls (*n* = 3). The internal reference actin was no difference between NFPAs and controls.

To validate the hub molecules, which were also overlapped molecules (DEGs; proteins) in NFPAs, western blotting was used to analyze the randomly selected hub molecules (SRC and AKT1). The results showed the fold-change of 1.31 for SRC and 1.01 for AKT (Figure 9). For SRC, western blotting analysis confirmed SRC was an unregulated DEG and its proteomic result. For AKT1, western blotting results demonstrated that AKT1 protein was expressed in NFPAs, but did not have significant difference between NFPAs and controls.

## Discussion

NFPA is a type of pituitary adenomas without specific clinical symptoms of hormone hypersecretion in its early stages (22). However, NFPA gradually grows up to compress its surrounding tissues, which results in visual defects and may progress to hypopituitarism. Thus, it is not easily diagnosed at its early stage, but often diagnosed at its middle or late stages. It is critically important to clarify molecular mechanisms of NFPAs for identification and development of more effective diagnostic and therapeutic strategies. The NFPA is a complex disease, and involves a series of molecular changes at the levels of genome, transcriptome, proteome, and metabolome. Multi-omics strategy is an effective approach to achieve those molecular changes in NFPAs (19, 20). This study integrated TMT-based quantitative proteomics and the public GEO transcriptomic data in NFPAs.

**Figure 10:**
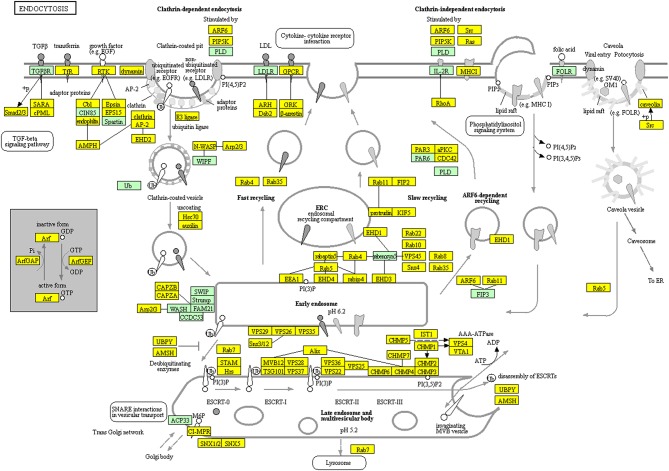
Endocytosis pathway altered in an NFPA. Yellow means the identified proteins.

**Figure 11:**
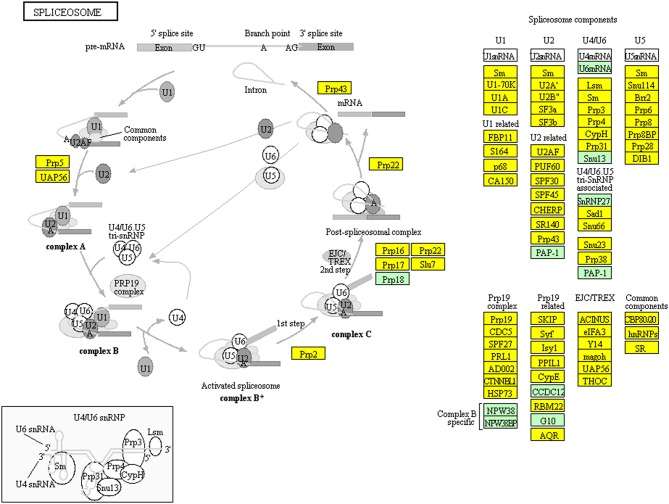
Spliceosome pathway altered in an NFPA. Yellow means the identified proteins.

TMT-based quantitative proteomics identified a total of 6076 proteins in NFPA tissues. A total of 114 statistically significant KEGG pathways were enriched with those 6076 proteins, including endocytosis and spliceosome pathways. KEGG pathway analysis found that 162 proteins among 6076 proteins were involved in endocytosis (Figure 10, Supplemental Table 7). Endocytosis was the basic process of all eukaryotic cells, including extracellular nutrient uptake, processing and presentation of antigens, apoptotic cell clearance, and cell surface protein regulation such as adhesion proteins, channel proteins, and receptors. Endocytosis and endocytic proteins regulated multiple biological processes, including cell apoptosis, cell cycle, and mitosis in cancer cells (23, 24). It is worth noting that 107 proteins among 6076 proteins were distributed in the spliceosome pathway (Figure 11, Supplemental Table 8). Spliceosome was an important way to regulate alternative splicing in cancer because spliceosomes made different transcripts for the same one gene in cancer cells, which was a driving factor for cancer progression (25). These pathways were involved in cellular energy metabolism, protein synthesis, and protein processing, which might significantly contribute to the pathogenesis of NFPAs.

Microarray and high throughput sequencing were widely used to detect and quantify the transcriptomic profile of the human genome, which is useful in the identification of target genes of interest for diagnosing or treating NFPAs (26). A total of 3598 DEGs was identified in NFPAs compared to controls with the public GEO transcriptomic data. An overlapping analysis was performed between 3598 DEG data and 6076 protein data, which obtained 1088 overlapped molecules (DEGs; proteins). It also means that these 1088 DEGs at the level of transcriptome, through transcriptional regulation and translation, ultimately functioned in the form of proteins in the body. The KEGG pathway analysis of those 1088 overlapped molecules (DEGs; proteins) revealed 52 statistically significantly KEGG pathways, including focal adhesion, cGMP/PKG, and platelet activation pathways.

**Figure 12:**
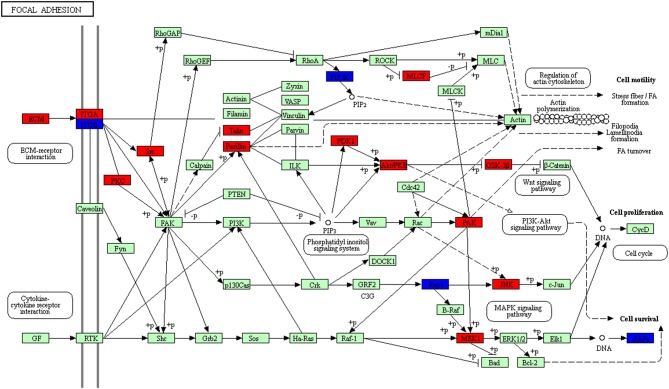
Focal adhesion pathway altered in an NFPA. Red means the upregulated overlapped molecules. Blue means the downregulated overlapped molecule.

The pathway with distribution of the most overlapped molecules (DEGs; proteins) was the focal adhesion pathway (Figure 12, Supplemental Table 9). Focal adhesion pathway consisted of many pro-survival signaling molecules such as growth factor receptors, intracellular molecules, and integrins. This pathway played important roles in the regulation of cell behavior and tumor cell survival, and might be cancer therapeutic targets (27). Thus, clarification of molecular processes of focal adhesion signaling might offer better insights into signal bypass and molecular mechanism of resistance, and help to develop reasonable multiple options of treatments. One of the important protein families in the pathway was the integrin family. FAK was a core mediator in the integrin signaling (28). FAK had three domains, including (i) focal adhesion targeting (FAT) region in the C-terminal, (ii) kinase domain in the central region, and (iii) band 4.1, ezrin, radixin and moesin (FERM) sequence in the N-terminal (29). Binding of integrin to ECM resulted in phosphorylation of FAK at several tyrosine residues including Tyr397 to increase kinase activities and promote the interaction of FAK with other proteins including SRC (30–32). One study found that FAK directly bond to cortactin (an actin regulator), which was not only a key force movement and focal adhesion conversion, but also affected cell survival (33). This study found 35 molecules (DEGs; proteins) distributed in this pathway (Supplemental Table 9), which suggested that the biological behavior of NFPA should be similar to that of other tumors, and that there should be a huge difference in cell adhesion between NFPAs and normal pituitaries. Previous studies examined the expression of FAK with immunohistochemistry in 49 human pituitary adenomas, and analyzed the relationship of FAK and invasiveness of pituitary adenomas. The results showed that there were 36 cases (73.5%) with FAK expression, and the expression level of FAK was highly correlated with invasiveness of pituitary adenomas, which clearly indicated that the integrin-focal adhesion kinase signaling pathway played a role in the invasion of pituitary adenomas (34). It also provided new ideas for one to study the pathogenesis and treatment of targeted drugs in pituitary adenomas.

**Figure 13:**
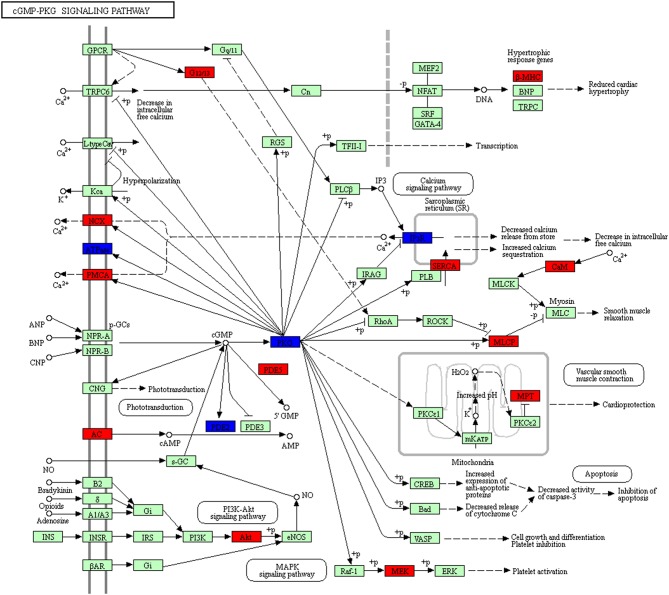
cGMP-PKG pathway altered in an NFPA. Red means the upregulated overlapped molecule. Blue means the downregulated overlapped molecule.

The cGMP/PKG pathway played complex roles in cancer, and differed in different tumor types and even in different model systems (32). This study found 23 molecules (DEGs; proteins) distributed in cGMP/PKG pathway (Figure 13, Supplemental Table 10). It has been reported that cGMP promoted tumorigenesis and anticancer effects. For example, the activated cGMP/PKG pathway induced multiple cancers (33, 35–37). Studies found specifically activated protein kinase G1 (PKG1) triggered MAPK signaling pathway to promote growth of melanoma (38). GMP analogs that activated PKG were a novel molecular strategy that interferes with tumor progression, and had attracted interest in oncology (32). Some studies demonstrated that cGMP/PKG pathway-targeted therapeutic potentials for multiple cancers (39, 40). This signaling was also involved in NFPA pathogenesis, with a wide distribution of DEGs from GEO database and of proteins identified with TMT-quantitative proteomics in human NFPAs. Generally NFPAs grow slowly, with low rate of invasiveness, it may be associated with activation of cGMP-PKG pathway. However, no cGMP/PKG pathway had been studied previously in NFPA yet. This present study provided a basis for further in-depth investigation of cGMP/PKG pathway in pituitary adenomas.

**Figure 14:**
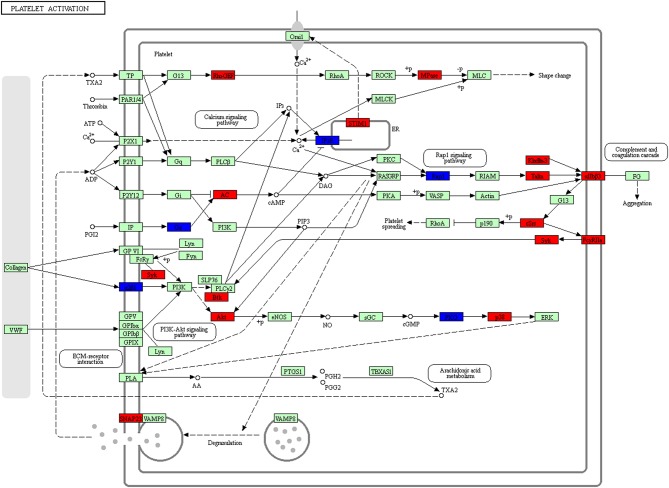
Platelet activation pathway altered in an NFPA. Red means the upregulated overlapped molecule. Blue means the downregulated overlapped molecule.

The platelet activation pathway was always in a prominent position in the list of enriched KEGG pathways (Figure 14, Supplemental Table 11). In recent years, the change of tumor microenvironment has become a research hotspot. Different tumor microenvironments were formed in each progression step of cancer and had multiple abilities to induce adverse and beneficial consequences of tumorigenesis (41). The roles of platelets in hemostasis and thrombosis were well-recognized. Platelets were also looked as multi-functional cells; for example, it recruited and regulated monocytes and granulocytes in tumor tissues, which indicated that platelet played important roles in production of tumor-associated macrophages/neutrophils (42). Many studies revealed that platelets were relevant to cancer biology as a hallmark of cancer, and that platelets participated in multistep processes of tumorigenesis (43, 44). In addition to its traditional role in hemostasis and thrombosis, platelets involved in hemostasis, thrombosis, inflammation and cancer interactions are complex and important in every disease process. Our study for the first time reported a correlation between platelet activation and the development of NFPAs.

Comparison of GEO transcriptomics data (DEGs) and proteomics data found that not all DEGs were expressed at the protein level. For many upregulated DEGs, the corresponding proteins were not detected. It reminded one not all DEGs in this study were transcribed into proteins to exert their functions. However, the molecular mechanisms of these DEGs without expressions at the protein level have not been fully understood yet. Among 1088 overlapped molecules (DEGs; proteins), the highest change in the GEO database was GH (log_2_FC = −12.56.). This means that GH was most significantly downregulated in NFPAs compared to normal pituitary tissues. It is consistent with previous research results (8, 13). Also, pituitary adenoma itself often results in the development of hypopituitarism, including GH deficiency (GHD). GHD has been reported in up to 85% of NFPA patients (45, 46).

The 1088 overlapped molecules (DEGs; proteins) were analyzed with String and Cytoscape software, and the hub molecules were obtained according to the degree, including GAPDH (degree = 148), ALB (degree = 143), ACACA (degree = 137), SRC (degree = 115), and AKT1 (degree = 114). GAPDH is glyceraldehyde-3-phosphate dehydrogenase, an enzyme in the glycolysis process. The gene encoding this enzyme is a housekeeping gene, which is highly expressed in almost all tissues. ALB is the main substance that maintains the body's nutrition and osmotic pressure. SRC is activated by different classes of receptors. The activated SRC regulates multiple biological processes such as cell adhesion, cell cycle, cell migration, immune response, cell transformation, and cell apoptosis (47). Studies found that when the SRC/FAK pathway was disturbed, FAK began to be depleted, and the isolation of FAK-high-efficiency cells or the expression of non-phosphorylated FAK proteins resulted in the active SRC to be isolated from focal adhesions into intracellular sites (48), and inhibition of autophagy recovered active SRC in the peripheral adhesion process to cause the death of cancer cells. Therefore, these proteins were considered to be the main targets of cancer treatment (49). In fact, SRC family kinases (SFKs) were overexpressed/overactivated in various malignancies, which was associated with poor disease progression and poor prognosis in patients with various cancers (50); for example, SRC was overexpressed in lung cancers (51). Some SFK inhibitors had successfully entered clinical trials, and were considered as the most potential drugs. For example, an SRC inhibitor BMS-354825 (aka Dasatinib) targeted all SFKs to inhibit the SRC signaling pathway (52). This present study also showed that SRC was a hub molecule and participated in multiple pathways in NFPAs. SRC inhibitors might be also potential target genes for the treatment of NFPA patients, which provided new ideas for treatment of NFPAs. Another hub molecule, acetyl-CoA carboxylase (ACACA) was a rate-limiting enzyme and upregulated in fatty acid synthetic pathway. ACACA was traditionally recognized as target of metabolic syndrome. However, studies found that malignant tumors had a strong capability for fatty acids synthesis (53), that ACACA was overexpressed in malignant cancers, and that the inhibition of ACACA resulted in cell-cycle arrest and apoptosis of cancer cells (54, 55). Thus, ACACA and some fatty acids might play important roles in cancer cell survival. Cancer cells could re-connect metabolic pathways from glycolysis-dependent patterns to lipogenesis-dependent patterns. AMPK was phosphorylated by an ACACA, which maintained cellular energy homeostasis to play an important role, while the cells were under stress (56, 57). Highly expressed lipogenic enzymes such as ACACA were integrated with certain signaling pathways that triggered tumorigenesis, invasion, and metastasis (58). The effects of abnormal metabolism on malignant transformations in cancer research have been underestimated for decades and may become another hallmark of anti-cancer and an attractive target for intervention. Akt kinase was an important protein in the PI3K-AKT-mTOR pathway and was activated by PI3K (phosphoinosidite-3-OH kinase). It was dysregulated in various tumors. Studies found that AKT was an important regulator in cell proliferation and apoptosis in the tumorigenesis process (59). Several studies demonstrated that AKT activation was associated with several tumor invasions (60). The PI3K-AKT pathway has been proposed to play an important role in the metabolic pathway of pituitary tumors, and future clinical studies should focus on the PI3K-AKT pathway for drug research and individualized treatment (61), which was consistent with our results regarding the overlapped molecule (DEG; protein)—AKT gene was upregulated in NFPAs compared to controls. Furthermore, this study used western blotting analysis to confirm that the expression of AKT1 protein in NFPA and control tissues although it was not changed significantly between NFPAs and controls. However, in our research group, another study found phosphorylated AKT1 in NFPA was significantly increased (62), which might lead to the activation of PI3K-AKT-mTOR pathway. In addition, studies also showed that low levels of APAF-1 were inversely related to invasiveness, and cathepsin B expression was positively related to invasiveness in pituitary adenomas (63). This TMT-quantitative proteomics identified multiple cathepsin family members, including cathepsin L1, cathepsin B, cathepsin G, cathepsin D, cathepsin Z, cathepsin S, cathepsin F, cathepsin W, and pro-cathepsin H, in human NFPA tissues (Supplemental Table 1). It clearly demonstrated that cathepsin family might play important roles in NFPA pathogenesis. Vascular endothelial growth factor (VEGF) and basic-fibroblast growth factor (bFGF) were significantly elevated in sera of pituitary adenoma patients (64). This TMT-quantitative proteomics found that FGF receptor 4 (FGFR4) and FGFR1 oncogene partner 2 (FGFR1OP2) were expressed in NFPA tissues. These findings demonstrated that these hub molecules (DEGs; proteins) were potential biomarkers for NFPAs.

For AKT1, western blotting results showed that AKT1 protein was expressed in NFPAs, but did not have significant difference between NFPAs and controls. This result indicated that AKT1 was a DEG at the level of transcriptome, but it was not a DEP at the level of proteome, between NFPAs and controls. It might be derived from different protein PTMs to produce different AKT1 proteoforms (65–68), which was confirmed by our another PTMScan experimental study that the phosphorylation levels at residues Ser473, Thr308, or Thr312 in AKT1 were significantly increased by at least 3 folds in NFPAs compared to controls (62). Therefore, the phosphorylated AKT1 (pAKT1) might contribute to NFPA pathogenesis.

Furthermore, a total of 6076 proteins identified from human NFPA tissues with TMT-based quantitative proteomics in this study (Supplemental Table 1) were compared to a total of 2175 proteins identified from human anterior pituitary gland with SDS-PAGE-LC-MS/MS and LC-LC-MS/MS (Supplemental Table 12) (69). A total of 1933 proteins were identified in both NFPAs and anterior pituitary gland, 242 proteins were only identified in anterior pituitary gland but not in NFPAs, 4143 proteins were only identified in NFPAs but not in anterior pituitary gland. It clearly demonstrated that much more proteins (*n* = 3,901 6,076–2,175) were identified in NFPAs compared to anterior pituitary gland. The main reason might be because we used more peptide fractions, longer LC gradient, and more sensitive mass spectrometer. Anyway, those two mapping proteomic data-sets from the corresponding NFPA tissues and anterior pituitary gland were precious resource to establish proteomic databases for human NFPA tissues and anterior pituitary glands, study the physiological functions of anterior pituitary glands and pathogenesis of NFPAs, and discover potential protein pattern biomarkers for NFPAs.

## Strenths and Limitations

### Strengths

NFPNA is involved in a series of molecule changes at the levels of DNAs (genome), RNAs (transcriptome), proteins (proteome), and metabolites (metabolome). Of them, transcriptome and proteome are the functional performers of genome. This study performed a large-scale of proteomics analysis of NFPAs (*n* = 6), and integrated the transcriptomics data in NFPAs (*n* = 8) vs. controls (*n* = 3) from GEO database, which found 6076 proteins in NFPA tissues, and 3598 DEGs in NFPAs vs. controls. Overlapping analysis obtained 1088 overlapped molecules (proteins; DEGs). Moreover, a set of data about signaling pathway network alterations were obtained based on those 1088 overlapped molecules. Those data are currently the biggest protein database and molecule network changes for NFPA tissues, which is precious resource to discover reliable biomarker pattern and explore in-depth molecular mechanisms for NFPAs.

### Limitations

*One* must note that for proteomics and transcriptomics analyses in this study, the sample size was not big, only 6 NFPA tissues for proteomics, and 8 NFPA tissues and 3 control tissues for transcriptomics. In future, it is necessary to significantly expand the tissue sample size for biomarker studies. Also, one must note that NFPA tissue is relative pure cell type in cell origin, while control pituitary tissue contains multiple pituitary cell types, which might cause bias when comparison is performed between NFPA and control pituitary tissues. However, this is a common problem for any researchers who study human pituitary adenoma tissues. In this study, more experiments such as western blotting among different NFPA and control tissues, and different omics methods were used to versify mutually the results, achieving the consistent results.

## Conclusion and Outlook

NFPA is a type of complex disease involved in multiple molecule changes at the levels of genome, transcriptome, and proteome. This study identified 6076 proteins with quantitative information in NFPAs with TMT-based quantitative proteomics, which was the large-scale quantitative protein reference map for human NFPA tissue proteome. Also, 3598 DEGs were identified between NFPAs and controls with the transcriptomic data from GEO database. Further, 1088 overlapped molecules (DEGs; proteins) between 6076 proteins and 3598 DEGs were analyzed to confirm that NFPAs differed from normal pituitary tissues in terms of FAK, cGMP/PKG, and platelet activation signaling pathways. In addition, the hub molecules derived from PPI network of those overlapped molecules (DEGs; proteins) were obtained and verified in NFPAs, which confirmed the differences between NFPA and normal pituitary tissues. These proteomic and transcriptomic data were the important resource to screen out new tumor biomarkers to form a pattern biomarker for diagnosis and target treatments, which is emerging as a great promise for NFPAs (19, 20) to achieve predictive, preventive, and personalized medicine (PPPM) for NFPA future research and clinical practice (70). These overlapped molecules are the important biomarker resource for sample stratification and clinical significance analysis (71). Thereby, in clinical applications, patients can be classified according to changes in these important molecules, providing better treatment options and predicting patient outcomes.

## Ethics Statement

The NFPA tissues were obtained from the Department of Neurosurgery, Xiangya Hospital, Central South University, and was approved by the Medical Ethics Committee of Xiangya Hospital of Central South University. Control pituitary glands were post-mortem tissues obtained from the Memphis Regional Medical Center, which were approved by University of Tennessee Health Science Center Internal Review Board (UTHSC-IRB). Consent was obtained from each patient or the family of control pituitary subject after full explanation of the purpose and nature of all procedures used.

## Author Contributions

TC analyzed data, performed Western blot experiment, prepared figures and tables, and wrote the manuscript. YW, ML, TZ, and BL participated in Western blot experiment and partial data analysis. XiaohanZ participated in language revisions and partial data analysis. XianquanZ conceived the concept, designed experiments and manuscript, collected the samples, obtained TMT quantitative proteomic data, instructed experiments and data analysis, supervised results, coordinated, critically revised and wrote the manuscript, and was responsible for its financial supports and the corresponding works. All authors approved the final manuscript.

### Conflict of Interest

The authors declare that the research was conducted in the absence of any commercial or financial relationships that could be construed as a potential conflict of interest.
