# Supplementary material for: Quantitative Analysis of Proteome in Non-functional Pituitary Adenomas: Clinical Relevance and Potential Benefits for the Patients
Source: Front Endocrinol (Lausanne). 2019 Dec 5;10:854. doi: 10.3389/fendo.2019.00854 (PMC6915109; doi:10.3389/fendo.2019.00854)

Supplemental Figure 1

52 statistically significant KEGG pathways enriched with 1088 overlapped molecules (DEGs; proteins)

1. Focal adhesion
2. Carbon metabolism
3. Platelet activation
4. Dopaminergic synapse
5. Human cytomegalovirus infection
6. Proteoglycans in cancer
7. Regulation of actin cytoskeleton
8. cGMP-PKG signaling pathway
9. Retrograde endocannabinoid signaling
10. Biosynthesis of amino acids
11. Thyroid hormone signaling pathway
12. Adrenergic signaling in cardiomyocytes
13. ECM-receptor interaction
14. Relaxin signaling pathway
15. Insulin signaling pathway
16. PPAR signaling pathway
17. Neurotrophin signaling pathway
18. Estrogen signaling pathway
19. Dilated cardiomyopathy (DCM)
20. Hypertrophic cardiomyopathy (HCM)
21. Circadian entrainment
22. Glucagon signaling pathway
23. Glycolysis / Gluconeogenesis
24. Phagosome
25. Salivary secretion
26. Aldosterone synthesis and secretion
27. Pancreatic secretion
28. Inflammatory mediator regulation of TRP channels
29. C-type lectin receptor signaling pathway
30. Cholinergic synapse
31. Gap junction
32. GnRH signaling pathway
33. HIF-1 signaling pathway
34. Fatty acid metabolism
35. Arrhythmogenic right ventricular cardiomyopathy (ARVC)
36. Small cell lung cancer
37. Adipocytokine signaling pathway
38. Amphetamine addiction
39. Gastric acid secretion
40. Glycine, serine and threonine metabolism
41. Arginine and proline metabolism
42. Ferroptosis
43. Cysteine and methionine metabolism
44. Pyruvate metabolism
45. Fatty acid degradation
46. Biosynthesis of unsaturated fatty acids
47. Citrate cycle (TCA cycle)
48. Pentose phosphate pathway
49. Glyoxylate and dicarboxylate metabolism
50. Mucin type O-glycan biosynthesis
51. Propanoate metabolism
52. Proximal tubule bicarbonate reclamation

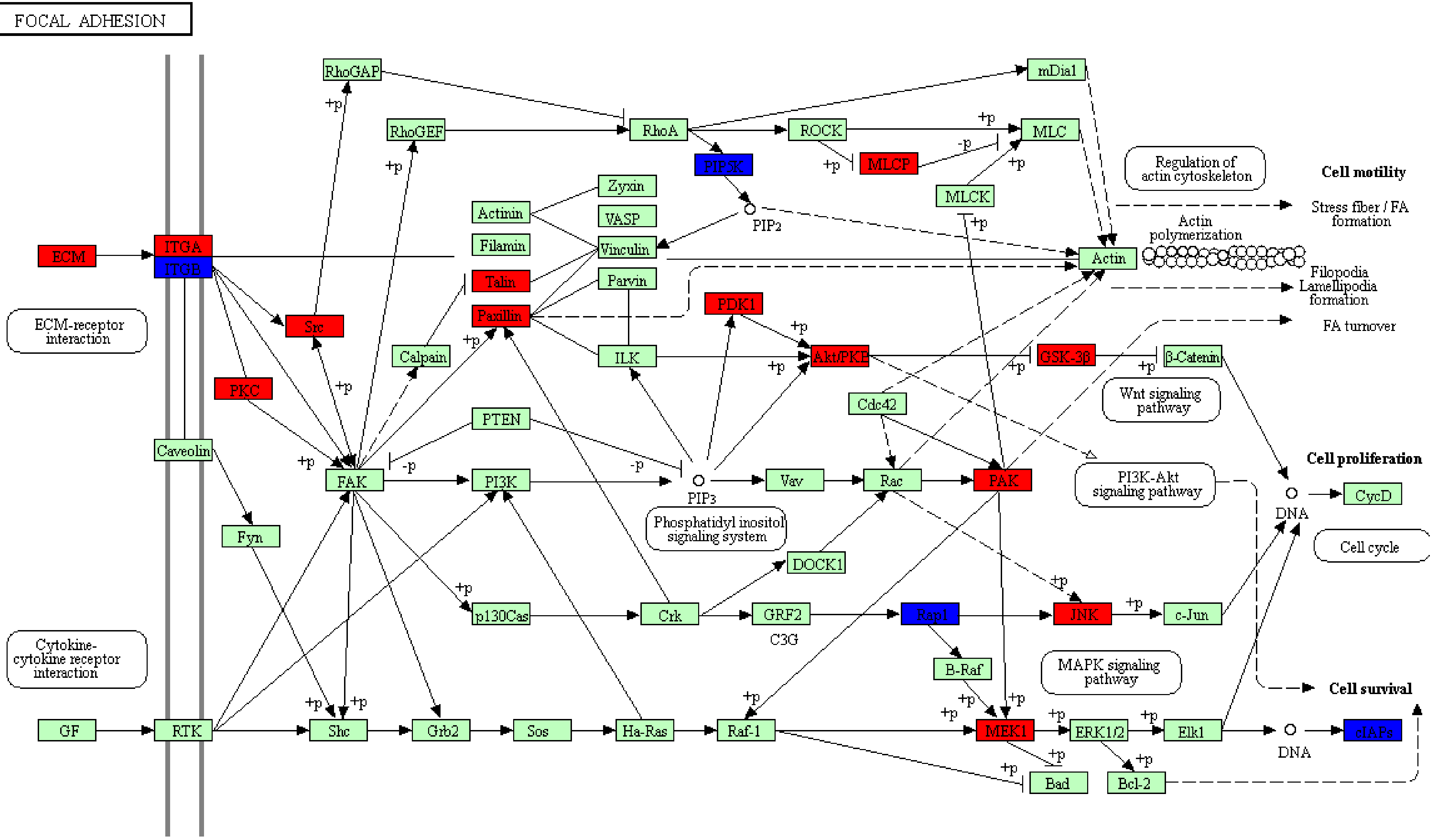
FOCAL ADHESION
RhoGAP
mDia1
RhoGEF
RhoA
ROCK
MLC
PIP5K
MLCP
Regulation of actin cytoskeleton
Cell motility
Zyxin
MLCK
Actinin
VASP
PIP2
Stress fiber / FA formation
Actin polymerization
Filamin
Vinculin
ECM
ITGA
ITGB
Actin
Talin
Parvin
Filopodia Lamellipodia formation
PDK1
Src
Paxillin
ECM-receptor interaction
FA turnover
Calpain
ILK
Akt/PKB
GSK-3β
β-Catenin
PKC
Wnt signaling pathway
Cdc42
PTEN
Caveolin
PI3K-Akt signaling pathway
Cell proliferation
FAK
PI3K
PIP3
Vav
Rac
PAK
DNA
CycD
Phosphatidyl inositol signaling system
Fyn
Cell cycle
DOCK1
p130Cas
Crk
GRF2
C3G
Rap1
JNK
c-Jun
Cytokine-cytokine receptor interaction
B-Raf
MAPK signaling pathway
Cell survival
GF
RTK
Shc
Grb2
Sos
Ha-Ras
Raf-1
MEK1
ERK1/2
Elk1
DNA
cIAPs
Bad
Bcl-2

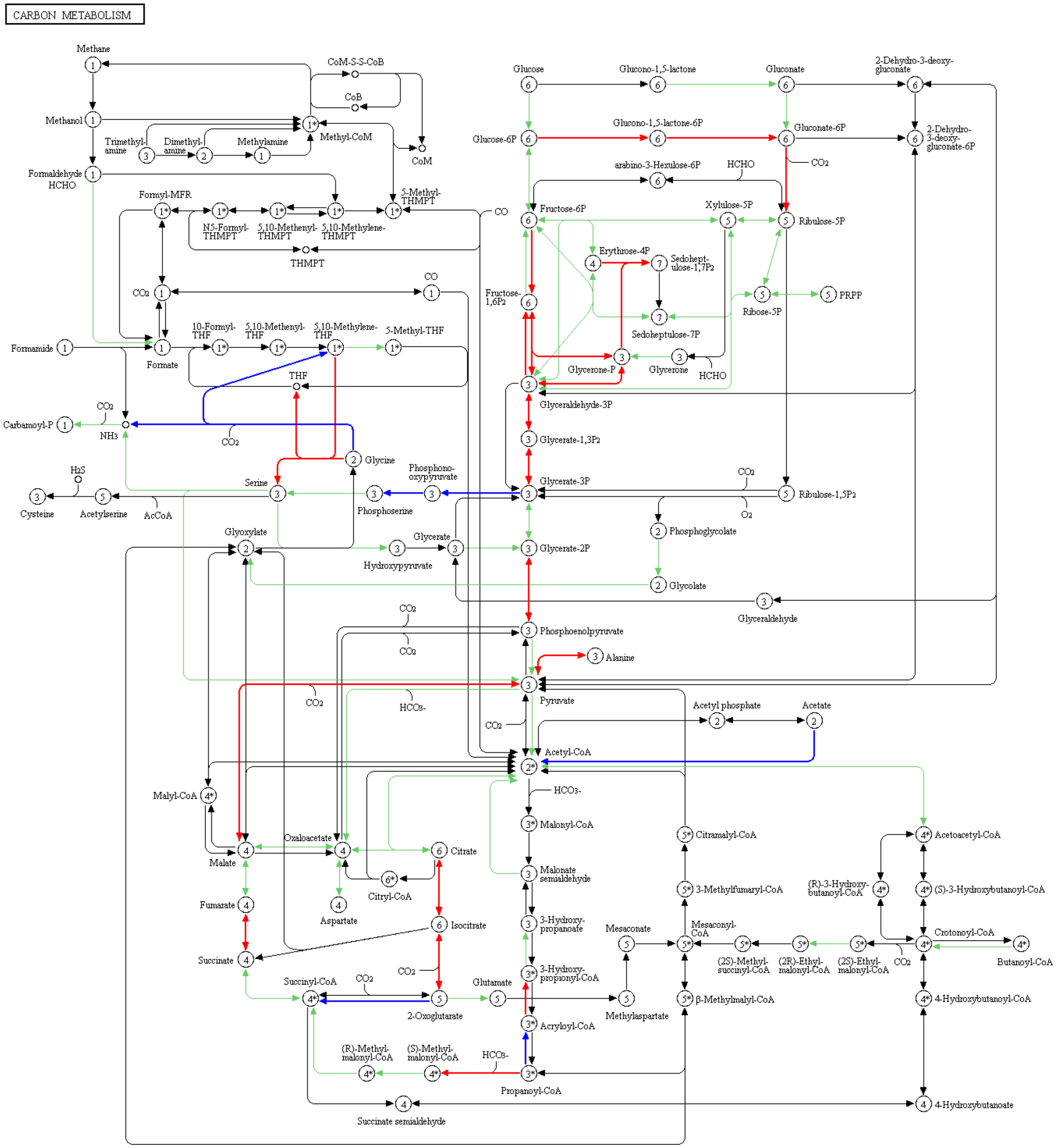

CARBON METABOLISM
Methane
CoM-S-S-CoB
CoB
Methanol
Methyl-CoM
CoM
Trimethyl-amine
Dimethyl-amine
Methylamine
Formaldehyde
HCHO
Formyl-MFR
5-Methyl-THMPT
N5-Formyl-THMPT
5,10-Methenyl-THMPT
5,10-Methylene-THMPT
THMPT
CO
CO2
10-Formyl-THF
5,10-Methenyl-THF
5,10-Methylene-THF
5-Methyl-THF
Formamide
Formate
THF
Carbamoyl-P
NH3
Glycine
Serine
H2S
Cysteine
Acetylserine
AcCoA
Phosphono-oxypyruvate
Phosphoserine
Glyoxylate
Glycerate
Hydroxypyruvate
Glucose
Glucono-1,5-lactone
Gluconate
2-Dehydro-3-deoxy-gluconate
Glucose-6P
Glucono-1,5-lactone-6P
Gluconate-6P
2-Dehydro-3-deoxy-gluconate-6P
arabino-3-Hexulose-6P
Fructose-6P
Xylulose-5P
Ribulose-5P
Erythrose-4P
Sedoheptulose-1,7P2
Fructose-1,6P2
Ribose-5P
PRPP
Sedoheptulose-7P
Glycerone-P
Glycerone
Glyceraldehyde-3P
Glycerate-1,3P2
Glycerate-3P
Ribulose-1,5P2
O2
Phosphoglycolate
Glycerate-2P
Glycolate
Glyceraldehyde
Phosphoenolpyruvate
Alanine
Pyruvate
HCO3-
Acetyl phosphate
Acetate
Acetyl-CoA
Malyl-CoA
Malonyl-CoA
Malonate semialdehyde
3-Hydroxy-propanoate
3-Hydroxy-propionyl-CoA
Acryloyl-CoA
Propanoyl-CoA
Oxaloacetate
Malate
Citrate
Citryl-CoA
Aspartate
Fumarate
Isocitrate
Succinate
Succinyl-CoA
2-Oxoglutarate
Glutamate
Mesaconate
Methylaspartate
(R)-Methyl-malonyl-CoA
(S)-Methyl-malonyl-CoA
Succinate semialdehyde
Citramalyl-CoA
3-Methylfumaryl-CoA
Mesaconyl-CoA
β-Methylmalyl-CoA
(2S)-Methyl-succinyl-CoA
(2R)-Ethyl-malonyl-CoA
(2S)-Ethyl-malonyl-CoA
Acetoacetyl-CoA
(R)-3-Hydroxy-butanoyl-CoA
(S)-3-Hydroxybutanoyl-CoA
Crotonoyl-CoA
Butanoyl-CoA
4-Hydroxybutanoyl-CoA
4-Hydroxybutanoate

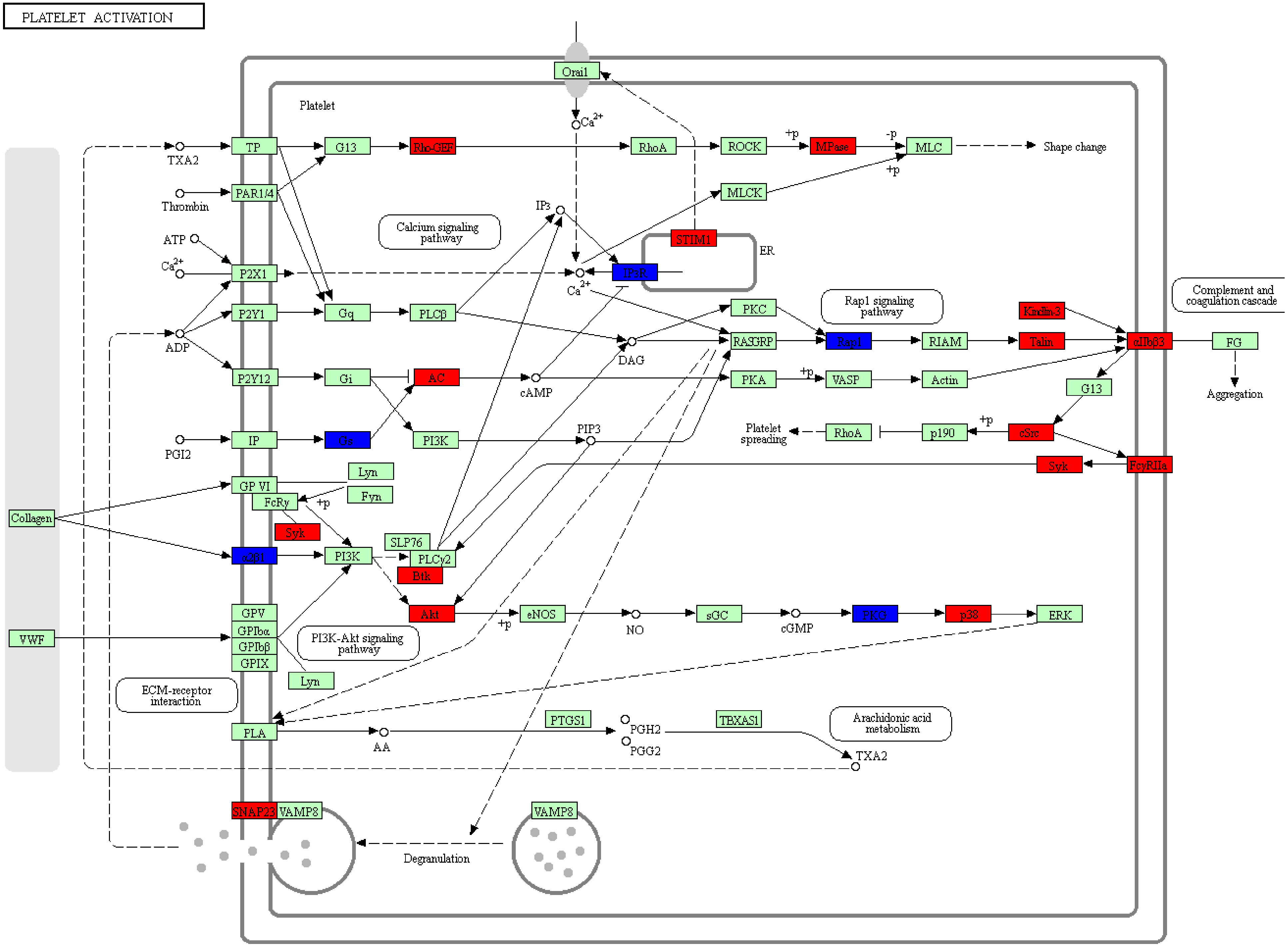
PLATELET ACTIVATION
Platelet
Orai1
Ca2+
TXA2
TP
G13
Rho-GEF
RhoA
ROCK
+p
MPase
-p
MLC
Shape change
+p
MLCK
Thrombin
PAR1/4
IP3
Calcium signaling pathway
STIM1
ER
ATP
Ca2+
P2X1
IP3R
Ca2+
Complement and coagulation cascade
P2Y1
Gq
PLCβ
PKC
Rap1 signaling pathway
Kindlin3
ADP
DAG
RASGRP
Rap1
RIAM
Talin
αIIbβ3
FG
P2Y12
Gi
AC
cAMP
PKA
+p
VASP
Actin
G13
Aggregation
PGI2
IP
Gs
PI3K
PIP3
Platelet spreading
RhoA
p190
+p
cSrc
Lyn
Syk
FcγRIIa
GP VI
Fyn
FcRγ
+p
Collagen
Syk
SLP76
α2β1
PI3K
PLCγ2
Btk
GPV
Akt
eNOS
+p
NO
sGC
cGMP
PKG
p38
ERK
VWF
GPIbα
GPIbβ
GPIX
PI3K-Akt signaling pathway
Lyn
ECM-receptor interaction
PTGS1
PGH2
TBXAS1
Arachidonic acid metabolism
PLA
AA
PGG2
TXA2
SNAP23
VAMP8
VAMP8
Degranulation

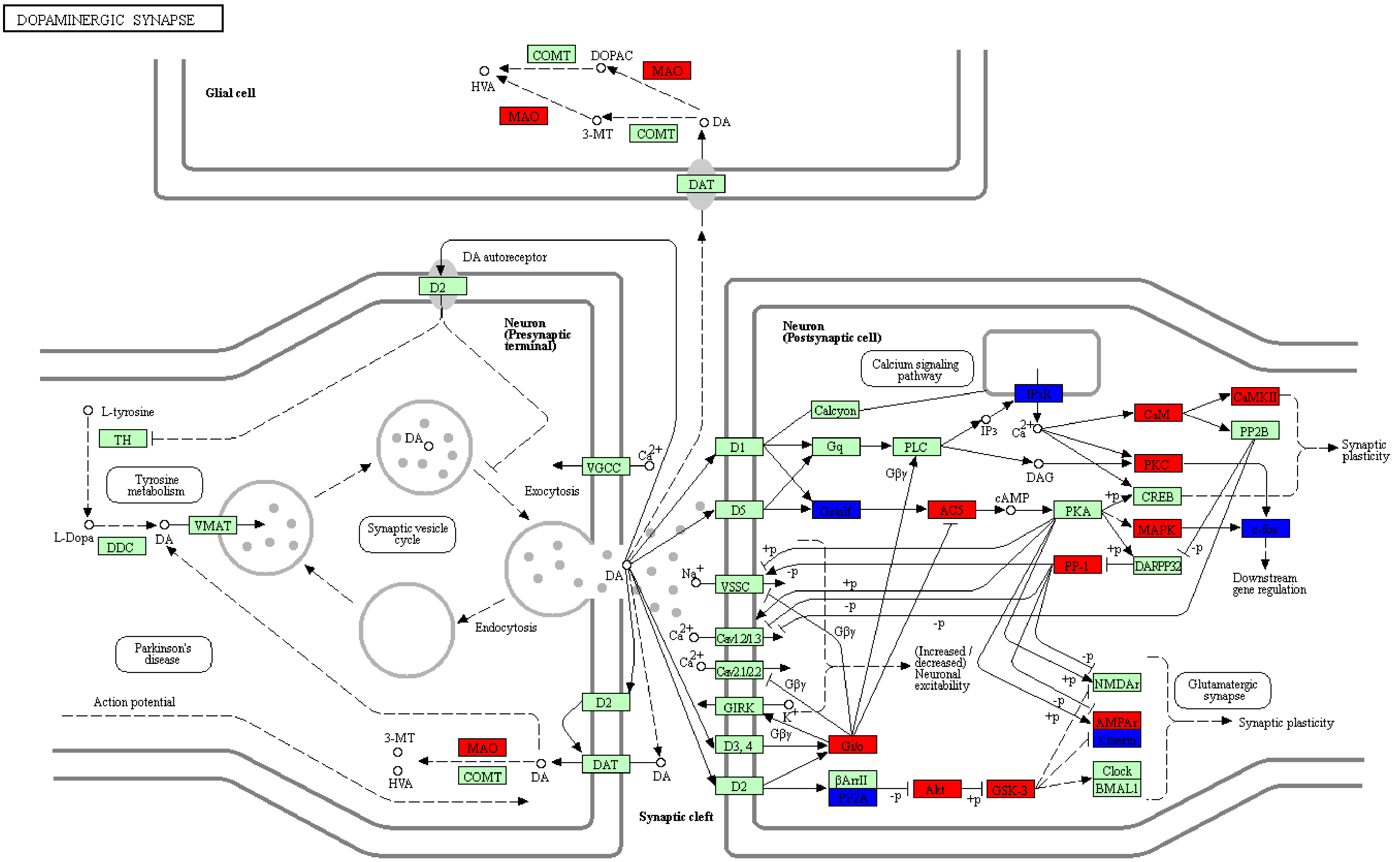
DOPAMINERGIC SYNAPSE
Glial cell
COMT
DOPAC
MAO
HVA
MAO
3-MT
COMT
DA
DAT
DA autoreceptor
D2
Neuron
(Presynaptic terminal)
L-tyrosine
TH
Tyrosine metabolism
L-Dopa
DDC
DA
VMAT
DA
Synaptic vesicle cycle
Exocytosis
VGCC
Endocytosis
Parkinson's disease
Action potential
3-MT
HVA
MAO
COMT
DA
D2
DAT
DA
Synaptic cleft
Neuron
(Postsynaptic cell)
Calcium signaling pathway
Calcyon
IP3R
CaM
CaMKII
PP2B
Synaptic plasticity
D1
Gq
PLC
IP3
DAG
PKC
CREB
D5
Gs/olf
AC5
cAMP
PKA
MAPK
c-fos
Downstream gene regulation
PP-1
DARPP32
VSSC
Cav1.2/1.3
Cav2.1/2.2
(Increased / decreased) Neuronal excitability
GIRK
D3, 4
Gi/o
D2
βArrII
PP2A
Akt
GSK-3
NMDAr
AMPAr
Kinesin
Clock
BMAL1
Glutamatergic synapse
Synaptic plasticity

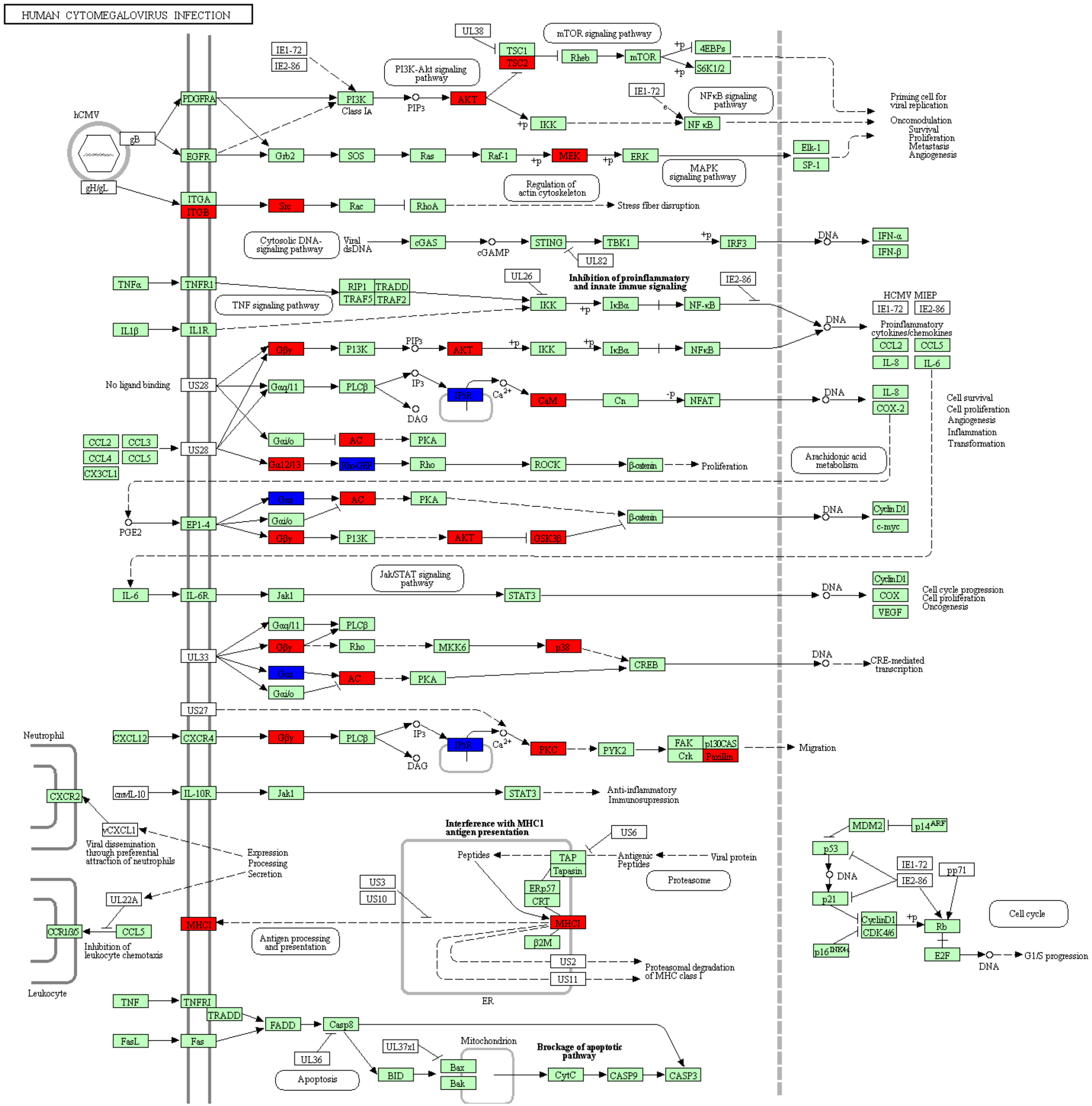
HUMAN CYTOMEGALOVIRUS INFECTION
hCMV
gB
gH/gL
PDGFRA
EGFR
ITGA
ITGB
IE1-72
IE2-86
PI3K
Class IA
PIP3
AKT
PI3K-Akt signaling pathway
UL38
TSC1
TSC2
Rheb
mTOR
mTOR signaling pathway
4EBPs
S6K1/2
IKK
NF κB
NFκB signaling pathway
Priming cell for viral replication
Oncomodulation
Survival
Proliferation
Metastasis
Angiogenesis
Grb2
SOS
Ras
Raf-1
MEK
ERK
Elk-1
SP-1
MAPK signaling pathway
Regulation of actin cytoskeleton
Src
Rac
RhoA
Stress fiber disruption
Cytosolic DNA-signaling pathway
Viral dsDNA
cGAS
cGAMP
STING
UL82
TBK1
IRF3
DNA
IFN-α
IFN-β
TNFα
TNFR1
RIP1
TRADD
TRAF5
TRAF2
TNF signaling pathway
UL26
Inhibition of proinflammatory and innate immune signaling
IκBα
NF-κB
HCMV MIEP
Proinflammatory cytokines/chemokines
CCL2
CCL5
IL-8
IL-6
IL1β
IL1R
Gβγ
No ligand binding
US28
Gαq/11
PLCβ
IP3
DAG
IP3R
Ca2+
CaM
Cn
NFAT
COX-2
Cell survival
Cell proliferation
Angiogenesis
Inflammation
Transformation
CCL3
CCL4
CX3CL1
Gαi/o
AC
PKA
Gα12/13
p115-RhoGEF
Rho
ROCK
β-catenin
Proliferation
Arachidonic acid metabolism
PGE2
EP1-4
Gαs
GSK3β
Cyclin D1
c-myc
Jak/STAT signaling pathway
IL-6R
Jak1
STAT3
COX
VEGF
Cell cycle progression
Cell proliferation
Oncogenesis
UL33
MKK6
p38
CREB
CRE-mediated transcription
US27
Neutrophil
CXCL12
CXCR4
PKC
PYK2
FAK
p130CAS
Crk
Paxillin
Migration
CXCR2
cmvIL-10
IL-10R
Anti-inflammatory
Immunosuppression
vCXCL1
Viral dissemination through preferential attraction of neutrophils
Expression
Processing
Secretion
UL22A
CCR1/3/5
Inhibition of leukocyte chemotaxis
Leukocyte
MHC1
Antigen processing and presentation
Interference with MHC1 antigen presentation
Peptides
TAP
Tapasin
US6
Antigenic Peptides
Viral protein
Proteasome
US3
US10
ERp57
CRT
β2M
US2
US11
Proteasomal degradation of MHC class I
ER
MDM2
p14ARF
p53
p21
pp71
CyclinD1
CDK4/6
p16INK4
Rb
E2F
G1/S progression
Cell cycle
TNF
TNFRI
FADD
Casp8
FasL
Fas
UL36
Apoptosis
BID
UL37x1
Mitochondrion
Bax
Bak
Brockage of apoptotic pathway
CytC
CASP9
CASP3

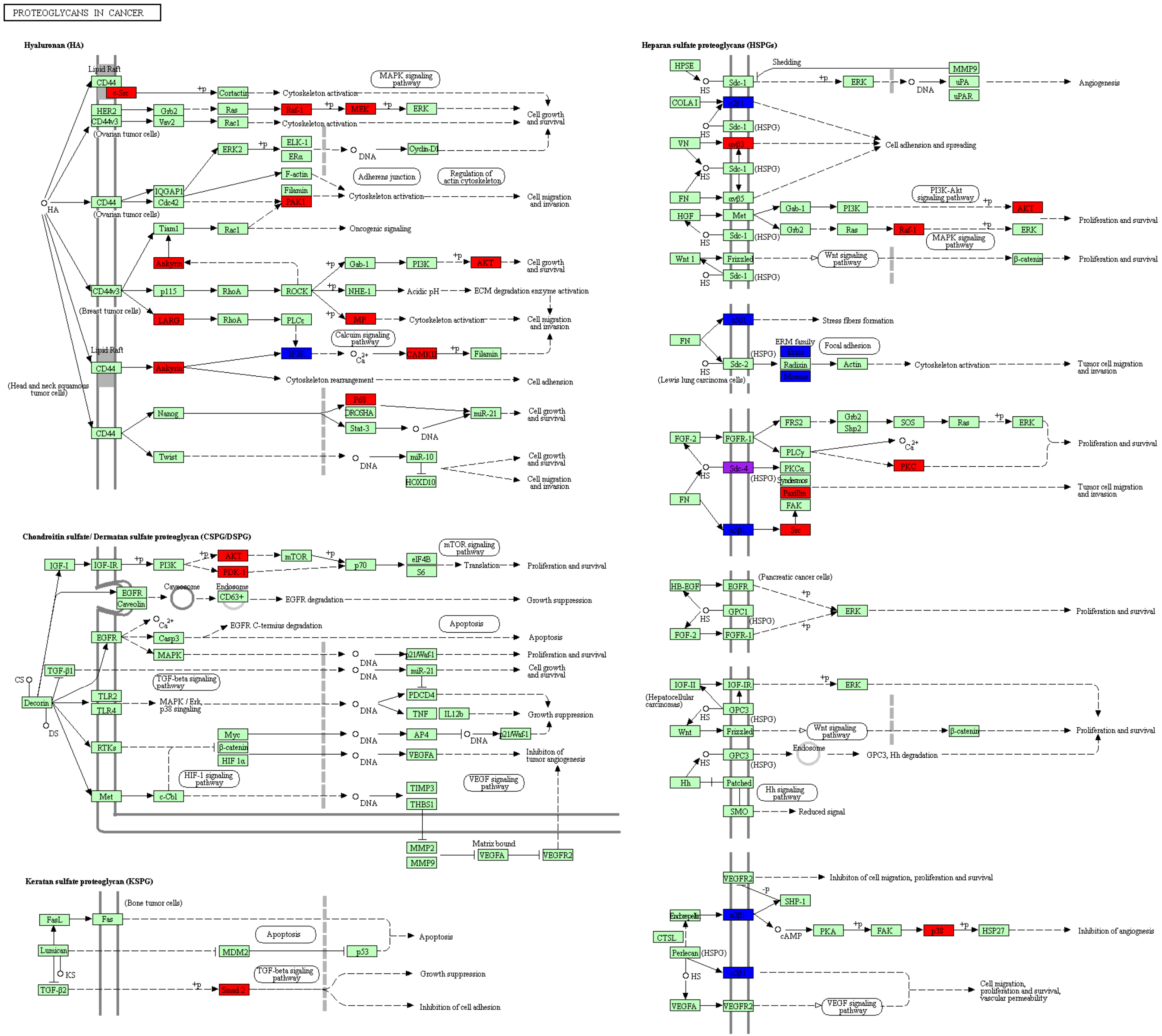

PROTEOGLYCANS IN CANCER
Hyaluronan (HA)
Lipid Raft
CD44
c-Src
Cortactin
Cytoskeleton activation
MAPK signaling pathway
HER2
CD44v3
Grb2
Vav2
Ras
Rac1
Raf-1
MEK
ERK
Cell growth and survival
(Ovarian tumor cells)
ERK2
ELK-1
ERα
DNA
Cyclin-D1
F-actin
Adherens junction
Regulation of actin cytoskeleton
HA
IQGAP1
Cdc42
Filamin
PAK1
Cell migration and invasion
Tiam1
Oncogenic signaling
Ankyrin
Gab-1
PI3K
AKT
p115
RhoA
ROCK
NHE-1
Acidic pH
ECM degradation enzyme activation
(Breast tumor cells)
LARG
PLCε
MP
Calcium signaling pathway
IP3R
Ca2+
CAMKII
Cell adhesion
Cytoskeleton rearrangement
(Head and neck squamous tumor cells)
P68
DROSHA
Stat-3
miR-21
Nanog
Twist
miR-10
HOXD10
Chondroitin sulfate/ Dermatan sulfate proteoglycan (CSPG/DSPG)
IGF-I
IGF-IR
PDK-1
mTOR
p70
eIF4B
S6
mTOR signaling pathway
Translation
Proliferation and survival
EGFR
Caveolin
Caveosome
Endosome
CD63+
EGFR degradation
Growth suppression
EGFR C-termius degradation
Apoptosis
Casp3
MAPK
p21/Waf-1
TGF-β1
TGF-beta signaling pathway
CS
Decorin
DS
TLR2
TLR4
MAPK / Erk, p38 singaling
PDCD4
TNF
IL12b
Myc
AP4
RTKs
β-catenin
HIF 1α
VEGFA
Inhibiton of tumor angiogenesis
HIF-1 signaling pathway
VEGF signaling pathway
Met
c-Cbl
TIMP3
THBS1
MMP2
MMP9
Matrix bound
VEGFR2
Keratan sulfate proteoglycan (KSPG)
(Bone tumor cells)
FasL
Fas
Lumican
KS
MDM2
p53
TGF-β2
Smad 2
Inhibition of cell adhesion
Heparan sulfate proteoglycans (HSPGs)
HPSE
HS
Sdc-1
Shedding
MMP9
uPA
uPAR
Angiogenesis
COLA I
α2β1
(HSPG)
VN
αvβ3
Cell adhesion and spreading
FN
αvβ5
PI3K-Akt signaling pathway
HGF
Met
Gab-1
Grb2
Raf-1
Proliferation and survival
Wnt 1
Frizzled
Wnt signaling pathway
β-catenin
α5β1
Stress fibers formation
ERM family
Ezrin
Radixin
Moesin
Focal adhesion
Actin
Sdc-2
Cytoskeleton activation
Tumor cell migration and invasion
(Lewis lung carcinoma cells)
FGF-2
FGFR-1
FRS2
Shp2
SOS
PLCγ
PKC
Sdc-4
PKCα
Syndesmos
Paxillin
FAK
Src
HB-EGF
(Pancreatic cancer cells)
GPC1
IGF-II
(Hepatocellular carcinomas)
GPC3
Wnt
GPC3, Hh degradation
Hh
Patched
Hh signaling pathway
SMO
Reduced signal
Inhibiton of cell migration, proliferation and survival
SHP-1
Endorepellin
cAMP
PKA
p38
HSP27
Inhibition of angiogenesis
CTSL
Perlecan
Cell migration, proliferation and survival, vascular permeability

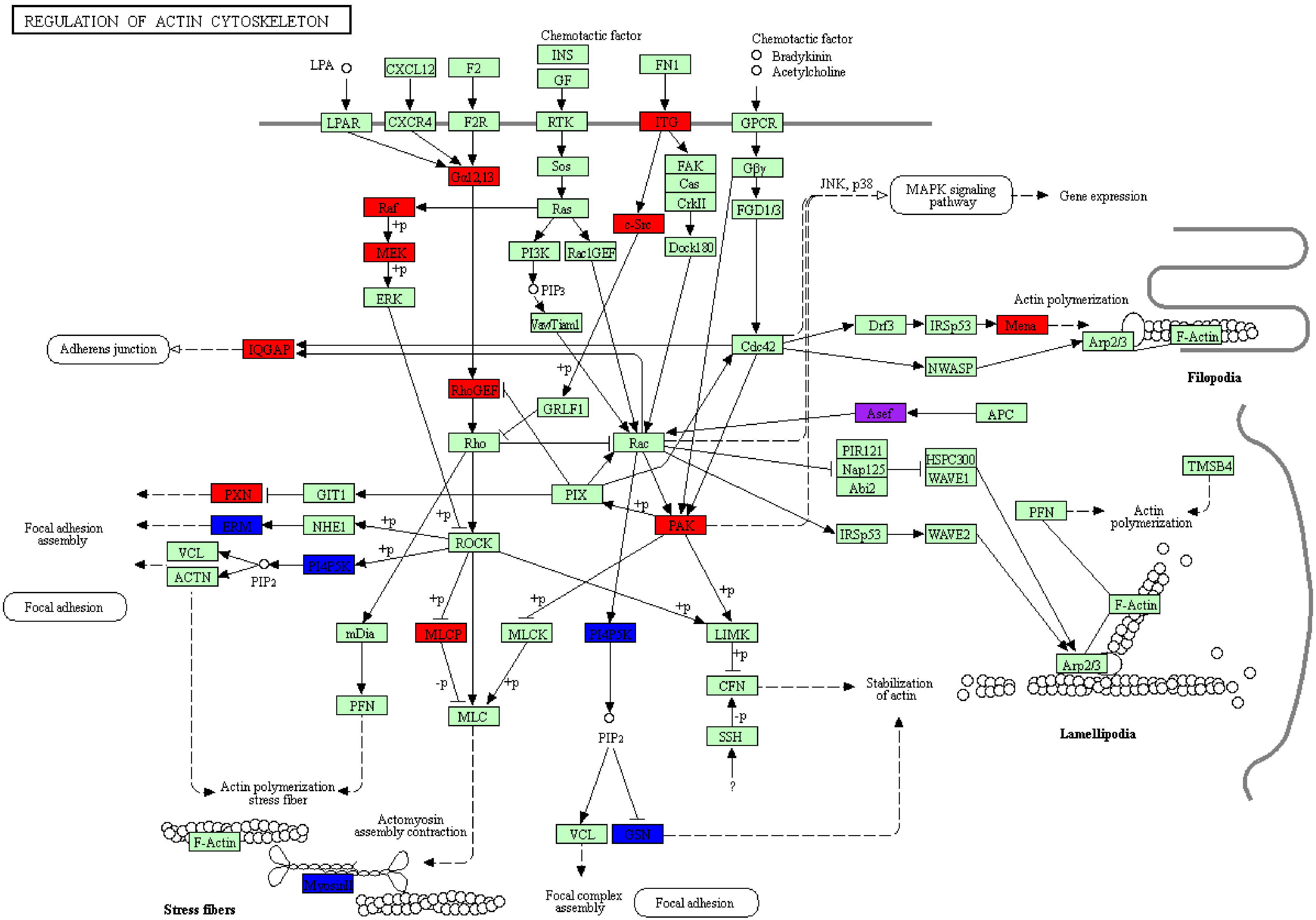
REGULATION OF ACTIN CYTOSKELETON
LPA
CXCL12
F2
Chemotactic factor
INS
GF
FN1
Chemotactic factor
Bradykinin
Acetylcholine
LPAR
CXCR4
F2R
RTK
ITG
GPCR
Gα12,13
Sos
FAK
Cas
CrkII
Gβγ
Raf
Ras
c-Src
FGD1/3
JNK, p38
MAPK signaling pathway
Gene expression
MEK
PI3K
Rac1GEF
Dock180
ERK
PIP3
VavTiam1
Actin polymerization
Drf3
IRSp53
Mena
Arp2/3
F-Actin
Adherens junction
IQGAP
Cdc42
NWASP
Filopodia
RhoGEF
GRLF1
Asef
APC
Rho
Rac
PIR121
Nap125
Abi2
HSPC300
WAVE1
TMSB4
PXN
GIT1
PIX
PFN
Actin polymerization
Focal adhesion assembly
ERM
NHE1
ROCK
PAK
IRSp53
WAVE2
VCL
ACTN
PI4P5K
PIP2
Focal adhesion
F-Actin
mDia
MLCP
MLCK
PI4P5K
LIMK
Arp2/3
CFN
Stabilization of actin
PFN
MLC
SSH
PIP2
Lamellipodia
Actin polymerization stress fiber
Actomyosin assembly contraction
VCL
GSN
F-Actin
MyosinII
Stress fibers
Focal complex assembly
Focal adhesion

cGMP-PKG SIGNALING PATHWAY

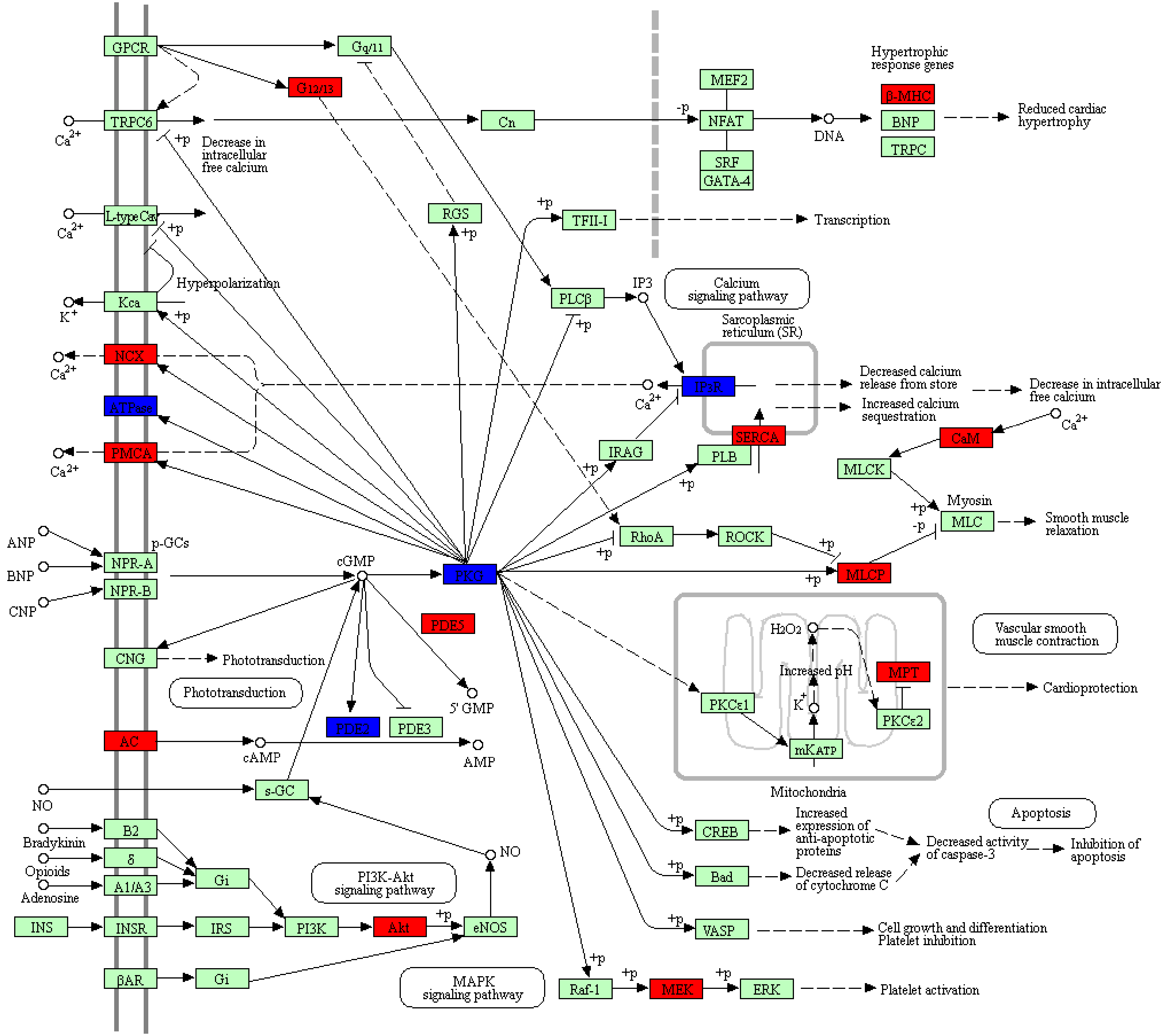

RETROGRADE ENDOCANNABINOID SIGNALING

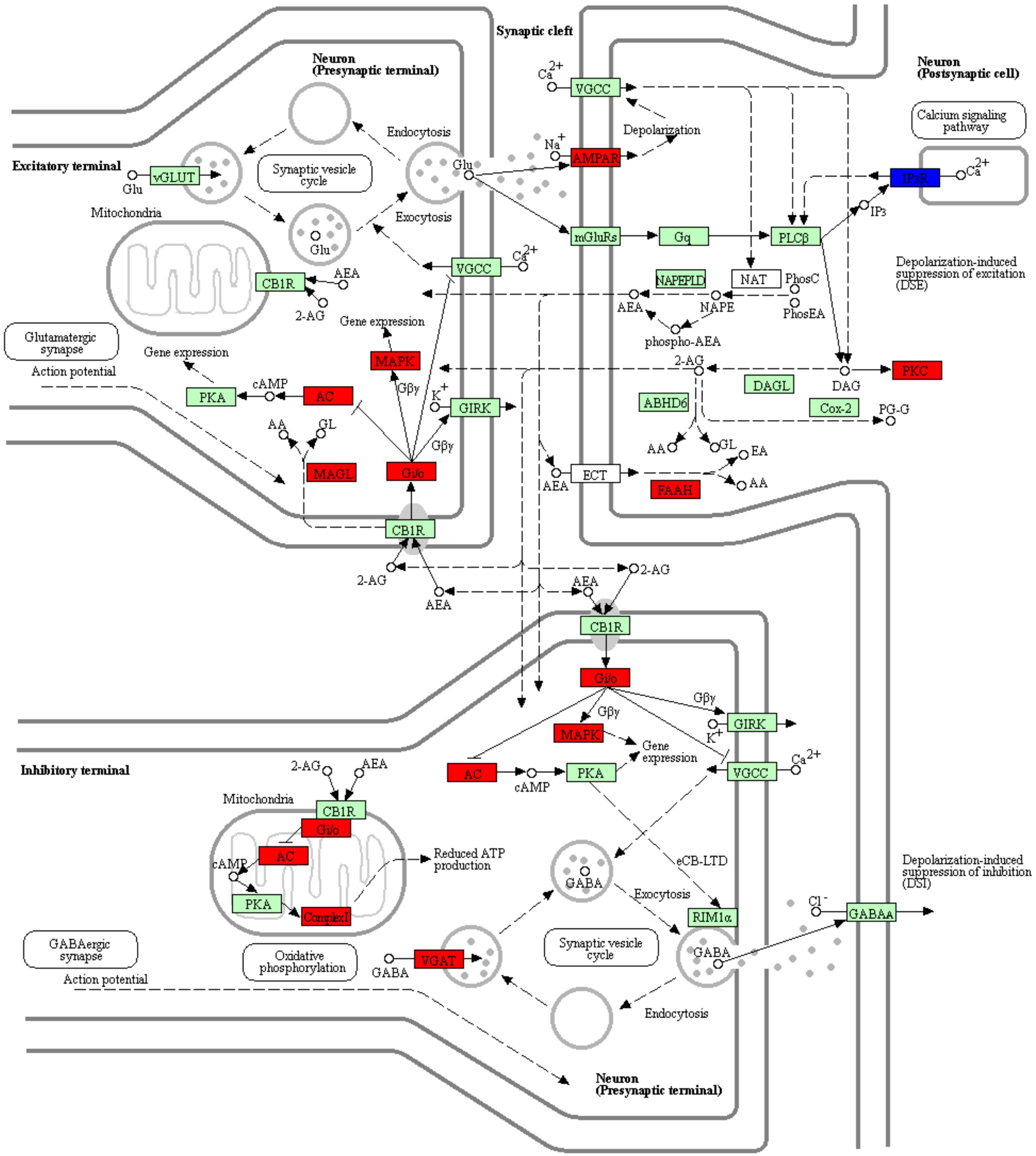

BIOSYNTHESIS OF AMINO ACIDS

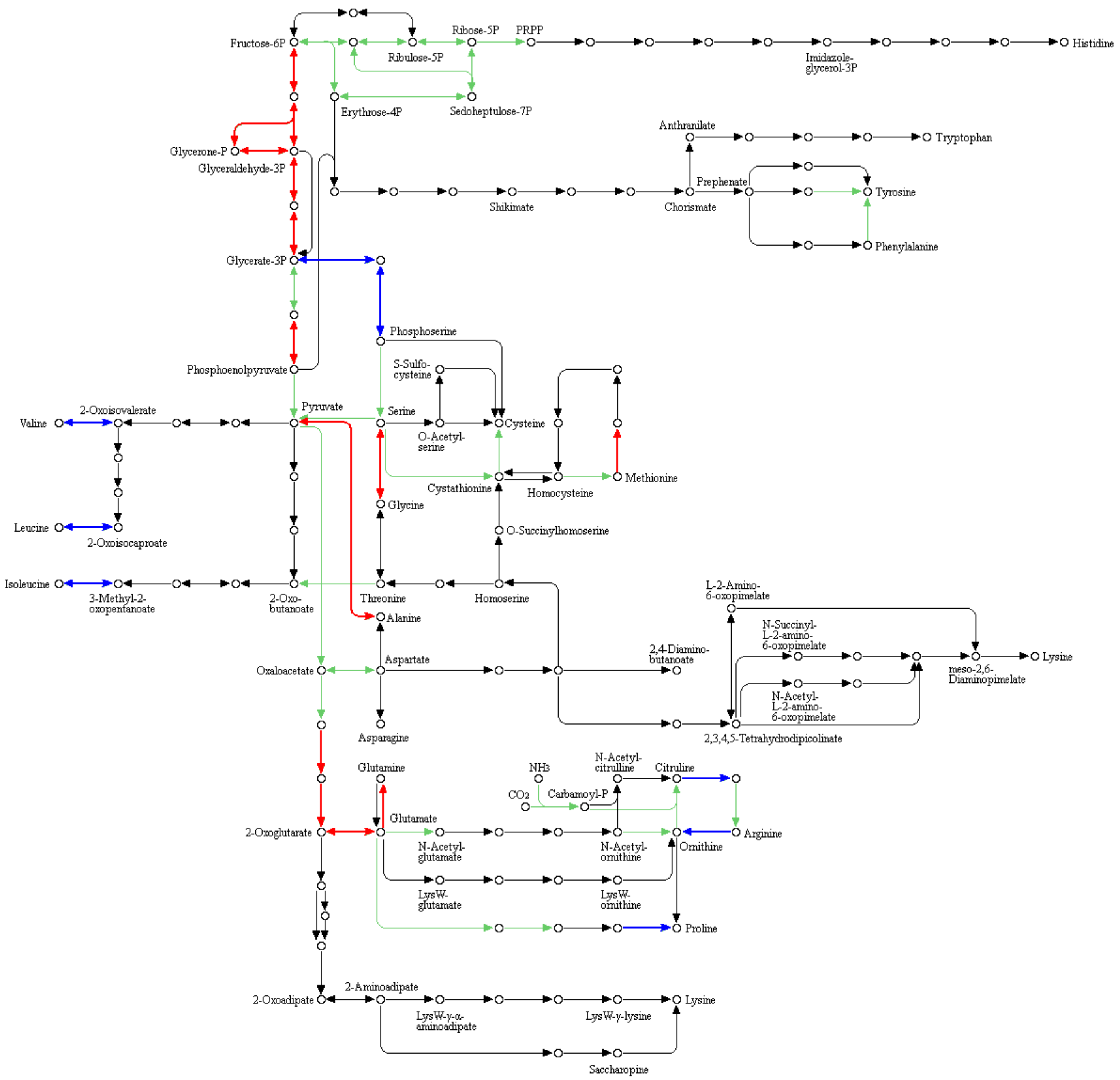

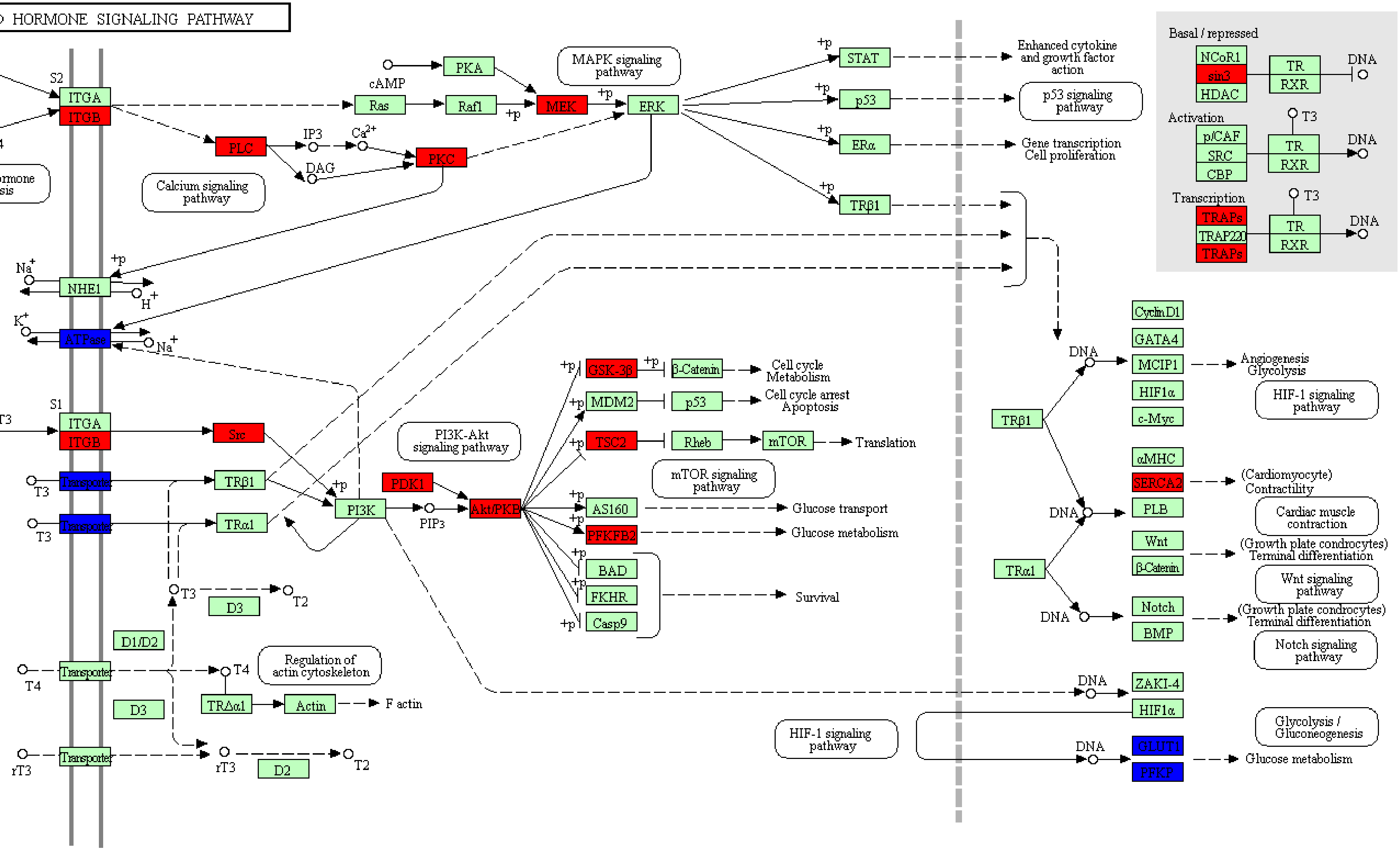
HORMONE SIGNALING PATHWAY
S2
ITGA
ITGB
cAMP
PKA
Ras
Raf1
MEK
ERK
+p
MAPK signaling pathway
PLC
IP3
Ca2+
DAG
PKC
Calcium signaling pathway
STAT
p53
ERα
TRβ1
Enhanced cytokine and growth factor action
p53 signaling pathway
Gene transcription Cell proliferation
Basal / repressed
NCoR1
sin3
HDAC
TR
RXR
DNA
Activation
T3
p/CAF
SRC
CBP
Transcription
TRAPs
TRAP220
Na+
NHE1
H+
K+
ATPase
S1
Src
Transporter
TRβ1
TRα1
T3
T4
rT3
PI3K
PIP3
PDK1
Akt/PKB
PI3K-Akt signaling pathway
GSK-3β
β-Catenin
Cell cycle Metabolism
MDM2
p53
Cell cycle arrest Apoptosis
TSC2
Rheb
mTOR
Translation
mTOR signaling pathway
AS160
Glucose transport
PFKFB2
Glucose metabolism
BAD
FKHR
Casp9
Survival
D3
T2
D1/D2
Regulation of actin cytoskeleton
TRΔα1
Actin
F actin
D2
CyclinD1
GATA4
MCIP1
HIF1α
c-Myc
Angiogenesis Glycolysis
HIF-1 signaling pathway
αMHC
SERCA2
PLB
(Cardiomyocyte) Contractility
Cardiac muscle contraction
Wnt
β-Catenin
(Growth plate condrocytes) Terminal differentiation
Wnt signaling pathway
Notch
BMP
Notch signaling pathway
ZAKI-4
HIF1α
GLUT1
PFKP
Glycolysis / Gluconeogenesis
Glucose metabolism

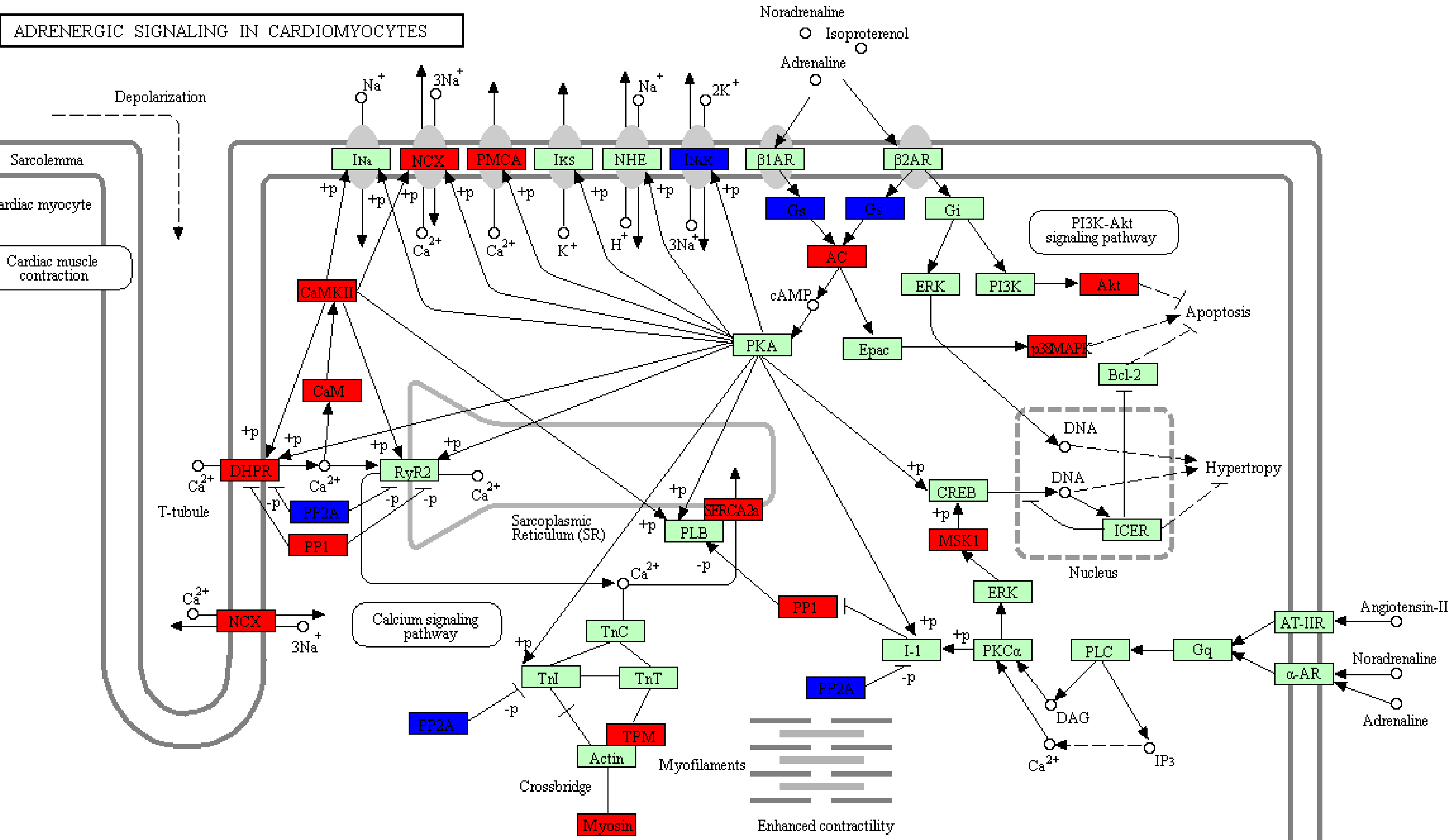
ADRENERGIC SIGNALING IN CARDIOMYOCYTES
Noradrenaline
Isoproterenol
Adrenaline
Depolarization
Sarcolemma
ardiac myocyte
Cardiac muscle contraction
Na+
3Na+
Na+
2K+
INa
NCX
PMCA
IKs
NHE
INaK
β1AR
β2AR
Ca2+
Ca2+
K+
H+
3Na+
Gs
Gs
Gi
AC
PI3K-Akt signaling pathway
cAMP
ERK
PI3K
Akt
Apoptosis
CaMKII
PKA
Epac
p38MAPK
Bcl-2
CaM
DNA
DNA
Hypertropy
DHPR
RyR2
CREB
ICER
MSK1
Nucleus
PP2A
PP1
SERCA2a
PLB
Sarcoplasmic Reticulum (SR)
T-tubule
ERK
Angiotensin-II
AT-IIR
NCX
Calcium signaling pathway
TnC
PP1
I-1
PKCα
PLC
Gq
Noradrenaline
α-AR
Adrenaline
TnI
TnT
PP2A
PP2A
DAG
TPM
Actin
Myofilaments
IP3
Crossbridge
Myosin
Enhanced contractility

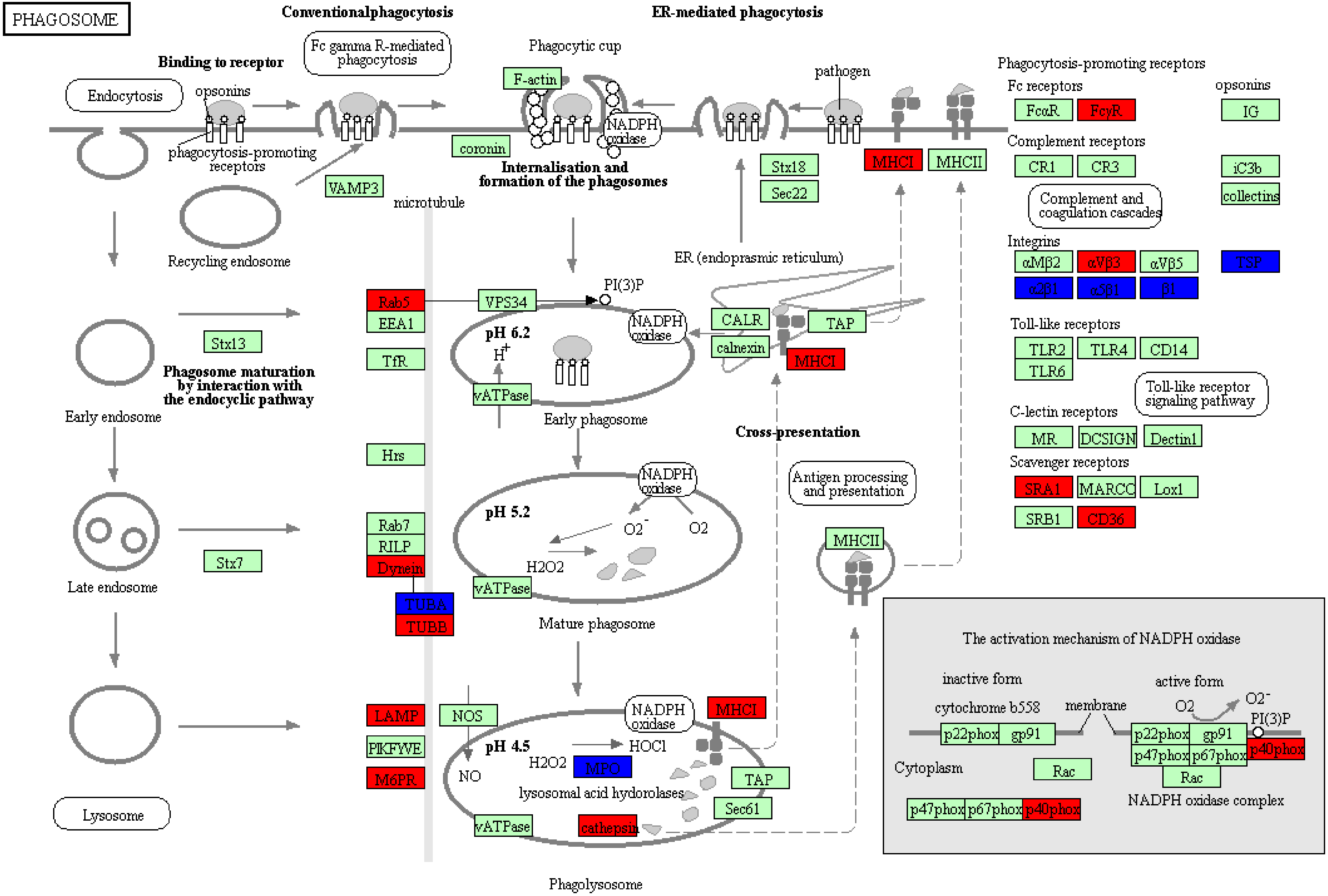
PHAGOSOME
Conventionalphagocytosis
ER-mediated phagocytosis
Binding to receptor
Fc gamma R-mediated phagocytosis
Phagocytic cup
Endocytosis
opsonins
phagocytosis-promoting receptors
F-actin
NADPH oxidase
pathogen
coronin
VAMP3
microtubule
Internalisation and formation of the phagosomes
Stx18
Sec22
MHCI
MHCII
Recycling endosome
ER (endoprasmic reticulum)
PI(3)P
Rab5
EEA1
VPS34
CALR
calnexin
TAP
Stx13
TfR
pH 6.2
H+
vATPase
Early phagosome
Phagosome maturation by interaction with the endocyclic pathway
Early endosome
Hrs
Cross-presentation
Antigen processing and presentation
pH 5.2
O2-
O2
H2O2
Rab7
RILP
Dynein
Stx7
Late endosome
TUBA
TUBB
Mature phagosome
LAMP
PIKFYVE
M6PR
NOS
NO
pH 4.5
H2O2
HOCl
MPO
lysosomal acid hydrolases
cathepsin
Sec61
Lysosome
Phagolysosome
Phagocytosis-promoting receptors
Fc receptors
FcαR
FcγR
opsonins
IG
Complement receptors
CR1
CR3
iC3b
collectins
Complement and coagulation cascades
Integrins
αMβ2
αVβ3
αVβ5
TSP
α2β1
α5β1
β1
Toll-like receptors
TLR2
TLR4
CD14
TLR6
Toll-like receptor signaling pathway
C-lectin receptors
MR
DCSIGN
Dectin1
Scavenger receptors
SRA1
MARCO
Lox1
SRB1
CD36
The activation mechanism of NADPH oxidase
inactive form
active form
cytochrome b558
membrane
p22phox
gp91
O2
O2-
PI(3)P
p47phox
p67phox
p40phox
Cytoplasm
Rac
NADPH oxidase complex

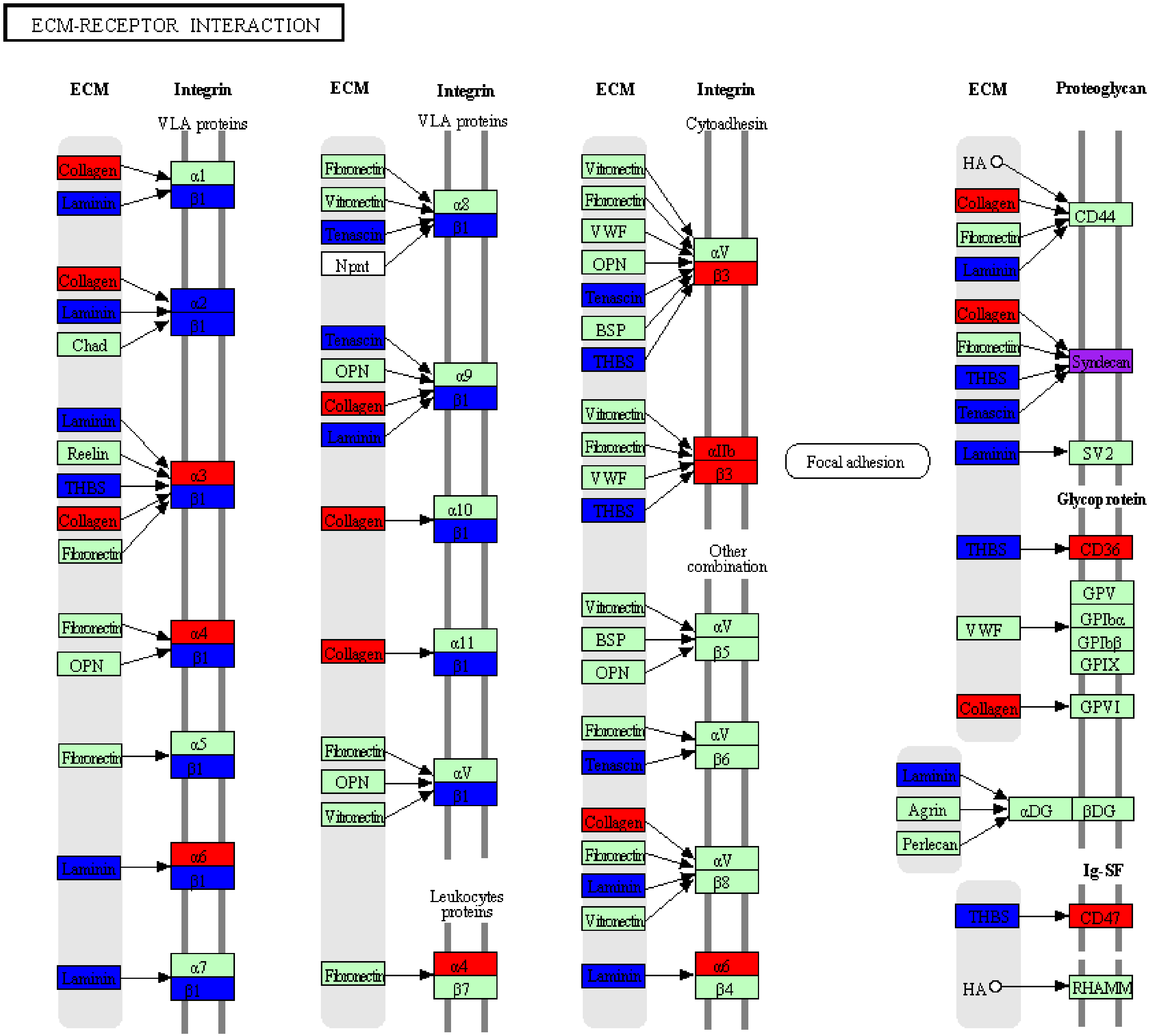
ECM-RECEPTOR INTERACTION
ECM
Integrin
VLA proteins
Collagen
Laminin
α1
β1
Collagen
Laminin
Chad
α2
β1
Laminin
Reelin
THBS
Collagen
Fibronectin
α3
β1
Fibronectin
OPN
α4
β1
Fibronectin
α5
β1
Laminin
α6
β1
Laminin
α7
β1
ECM
Integrin
VLA proteins
Fibronectin
Vitronectin
Tenascin
Npnt
α8
β1
Tenascin
OPN
Collagen
Laminin
α9
β1
Collagen
α10
β1
Collagen
α11
β1
Fibronectin
OPN
Vitronectin
αV
β1
Leukocytes proteins
Fibronectin
α4
β7
ECM
Integrin
Cytoadhesin
Vitronectin
Fibronectin
VWF
OPN
Tenascin
BSP
THBS
αV
β3
Vitronectin
Fibronectin
VWF
THBS
αIIb
β3
Other combination
Vitronectin
BSP
OPN
αV
β5
Fibronectin
Tenascin
αV
β6
Collagen
Fibronectin
Laminin
Vitronectin
αV
β8
Laminin
α6
β4
Focal adhesion
ECM
Proteoglycan
HA
Collagen
Fibronectin
Laminin
CD44
Collagen
Fibronectin
THBS
Tenascin
Syndecan
Laminin
SV2
Glycoprotein
THBS
CD36
VWF
GPV
GPIbα
GPIbβ
GPIX
Collagen
GPVI
Laminin
Agrin
Perlecan
αDG
βDG
Ig-SF
THBS
CD47
HA
RHAMM

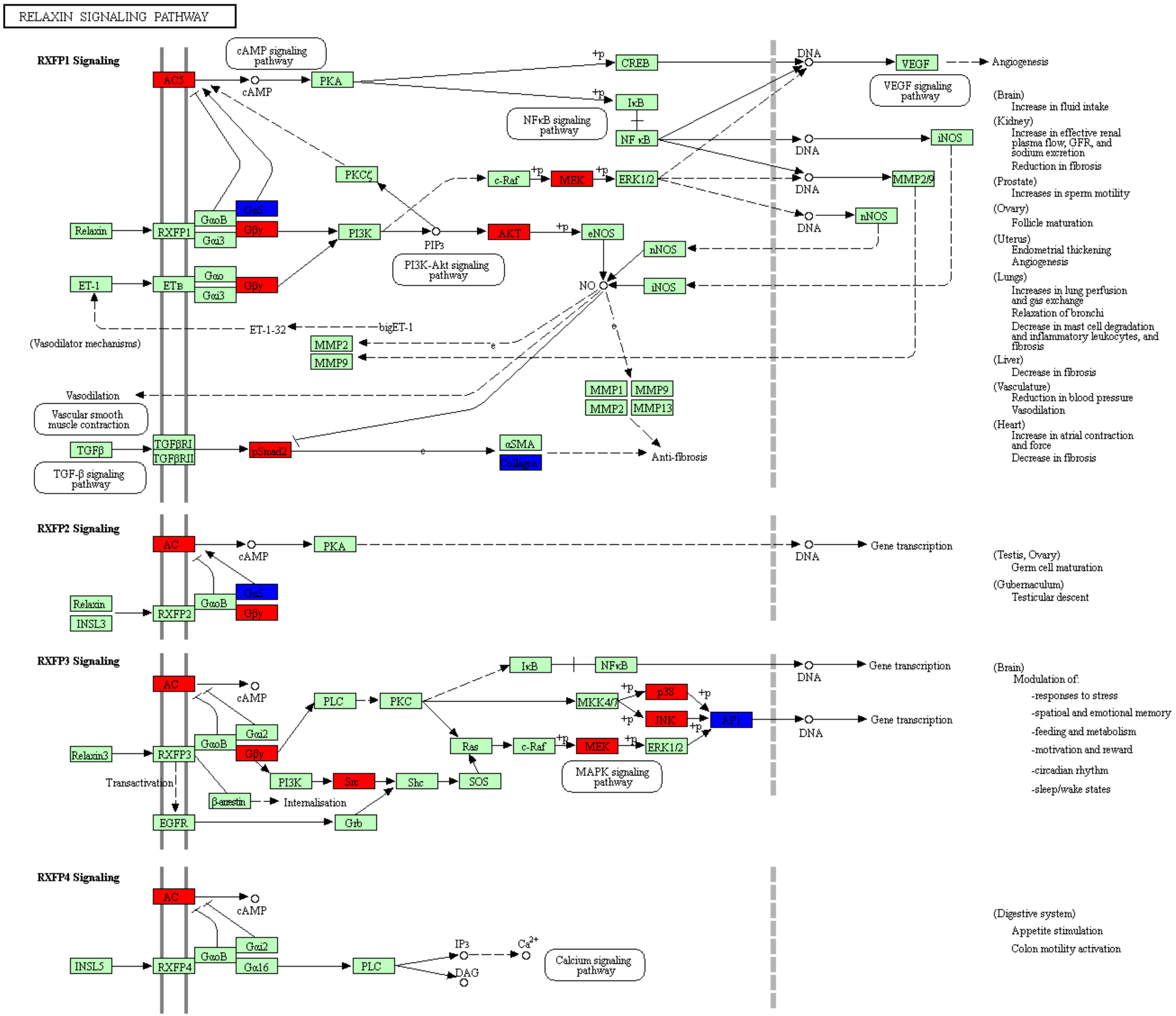

RELAXIN SIGNALING PATHWAY
RXFP1 Signaling
RXFP2 Signaling
RXFP3 Signaling
RXFP4 Signaling
cAMP signaling pathway
VEGF signaling pathway
NFκB signaling pathway
PI3K-Akt signaling pathway
TGF-β signaling pathway
MAPK signaling pathway
Calcium signaling pathway
Angiogenesis
(Vasodilator mechanisms)
Vasodilation
Vascular smooth muscle contraction
Anti-fibrosis
Gene transcription
Transactivation
Internalisation
(Brain) Increase in fluid intake
(Kidney) Increase in effective renal plasma flow, GFR, and sodium excretion
Reduction in fibrosis
(Prostate) Increases in sperm motility
(Ovary) Follicle maturation
(Uterus) Endometrial thickening
Angiogenesis
(Lungs) Increases in lung perfusion and gas exchange
Relaxation of bronchi
Decrease in mast cell degradation and inflammatory leukocytes, and fibrosis
(Liver) Decrease in fibrosis
(Vasculature) Reduction in blood pressure
Vasodilation
(Heart) Increase in atrial contraction and force
Decrease in fibrosis
(Testis, Ovary) Germ cell maturation
(Gubernaculum) Testicular descent
(Brain) Modulation of:
-responses to stress
-spatial and emotional memory
-feeding and metabolism
-motivation and reward
-circadian rhythm
-sleep/wake states
(Digestive system)
Appetite stimulation
Colon motility activation

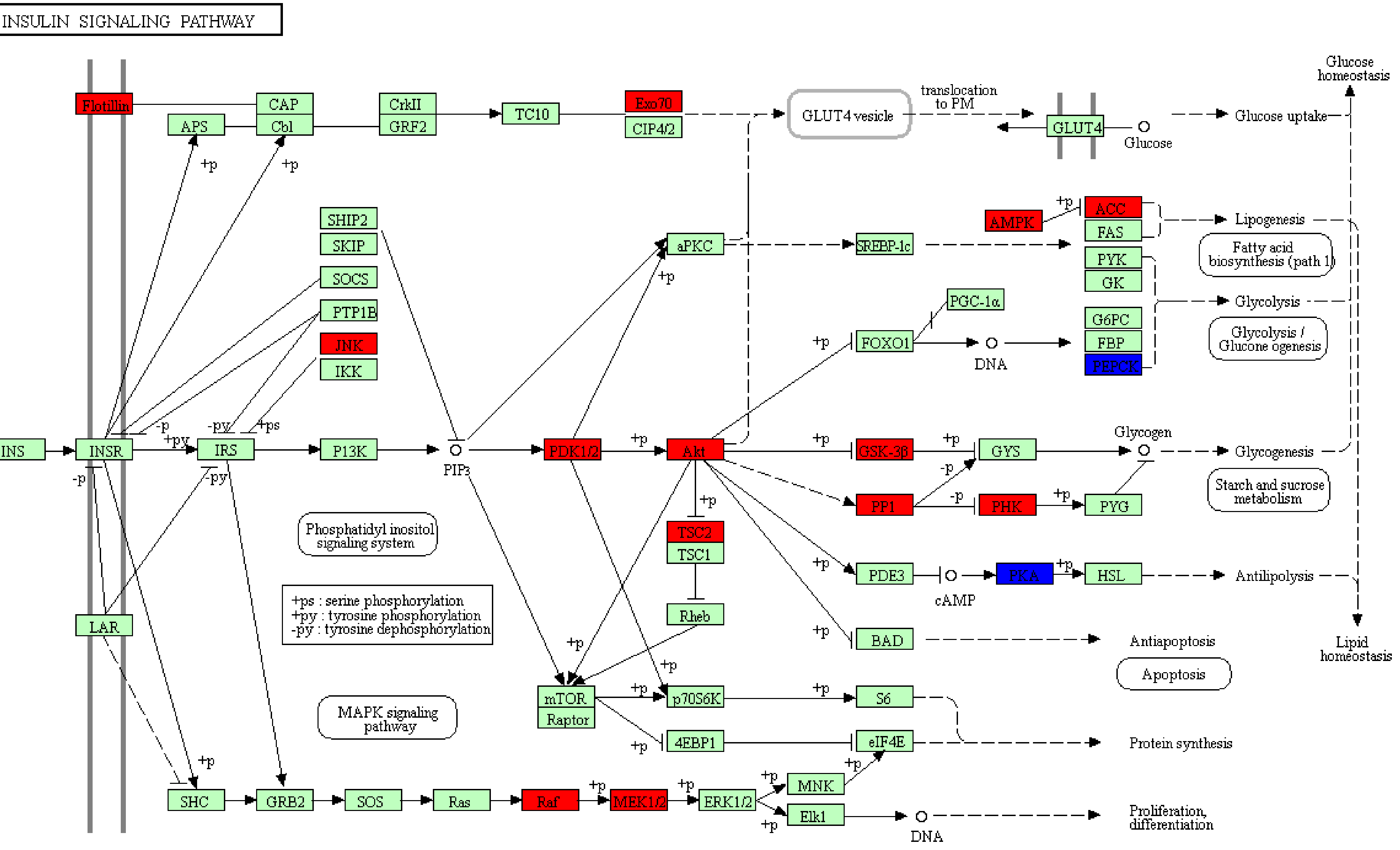
INSULIN SIGNALING PATHWAY
Flotillin
CAP
Cbl
APS
CrkII
GRF2
TC10
Exo70
CIP4/2
GLUT4 vesicle
translocation to PM
GLUT4
Glucose
Glucose homeostasis
Glucose uptake
SHIP2
SKIP
SOCS
PTP1B
JNK
IKK
aPKC
SREBP-1c
AMPK
ACC
FAS
PYK
GK
G6PC
FBP
PEPCK
PGC-1α
FOXO1
DNA
Lipogenesis
Fatty acid biosynthesis (path 1)
Glycolysis
Glycolysis / Gluconeogenesis
INS
INSR
IRS
P13K
PIP3
PDK1/2
Akt
GSK-3β
GYS
Glycogen
Glycogenesis
Starch and sucrose metabolism
PP1
PHK
PYG
TSC2
TSC1
Rheb
PDE3
cAMP
PKA
HSL
Antilipolysis
Lipid homeostasis
BAD
Antiapoptosis
Apoptosis
Phosphatidyl inositol signaling system
+ps : serine phosphorylation
+py : tyrosine phosphorylation
-py : tyrosine dephosphorylation
LAR
mTOR
Raptor
p70S6K
S6
4EBP1
eIF4E
Protein synthesis
MAPK signaling pathway
SHC
GRB2
SOS
Ras
Raf
MEK1/2
ERK1/2
MNK
Elk1
Proliferation, differentiation
+p
-p
+py
-py
+ps

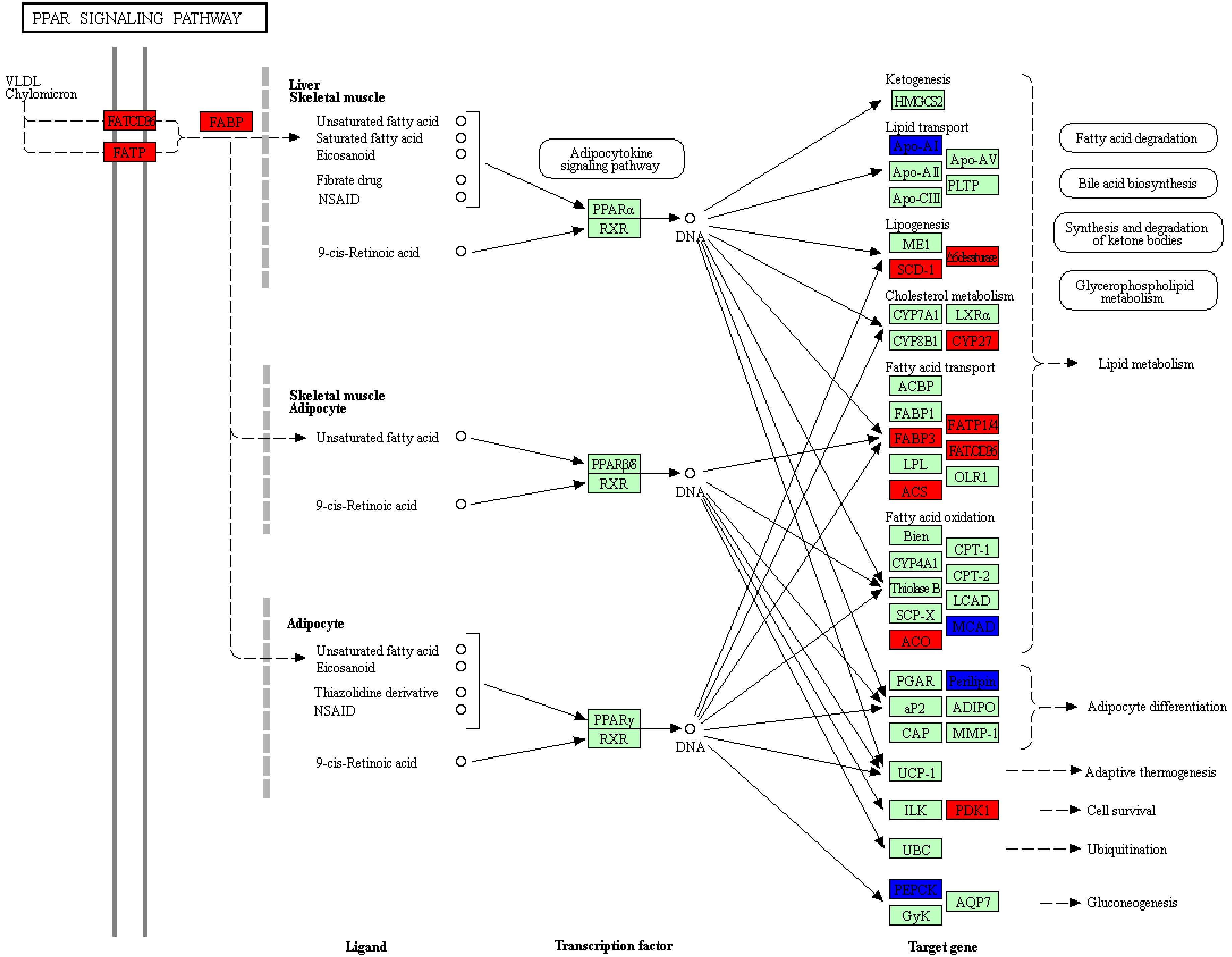
PPAR SIGNALING PATHWAY
VLDL
Chylomicron
FAT/CD36
FATP
FABP
Liver
Skeletal muscle
Unsaturated fatty acid
Saturated fatty acid
Eicosanoid
Fibrate drug
NSAID
9-cis-Retinoic acid
Adipocytokine signaling pathway
PPARα
RXR
DNA
Skeletal muscle
Adipocyte
Unsaturated fatty acid
9-cis-Retinoic acid
PPARβ/δ
RXR
DNA
Adipocyte
Unsaturated fatty acid
Eicosanoid
Thiazolidine derivative
NSAID
9-cis-Retinoic acid
PPARγ
RXR
DNA
Ketogenesis
HMGCS2
Lipid transport
Apo-AI
Apo-AV
Apo-AII
PLTP
Apo-CIII
Lipogenesis
ME1
Δ9-desaturase
SCD-1
Cholesterol metabolism
CYP7A1
LXRα
CYP8B1
CYP27
Fatty acid transport
ACBP
FABP1
FATP1/4
FABP3
FAT/CD36
LPL
OLR1
ACS
Fatty acid oxidation
Bien
CPT-1
CYP4A1
CPT-2
Thiolase B
LCAD
SCP-X
MCAD
ACO
PGAR
Perilipin
aP2
ADIPO
CAP
MMP-1
UCP-1
ILK
PDK1
UBC
PEPCK
AQP7
GyK
Fatty acid degradation
Bile acid biosynthesis
Synthesis and degradation of ketone bodies
Glycerophospholipid metabolism
Lipid metabolism
Adipocyte differentiation
Adaptive thermogenesis
Cell survival
Ubiquitination
Gluconeogenesis
Ligand
Transcription factor
Target gene

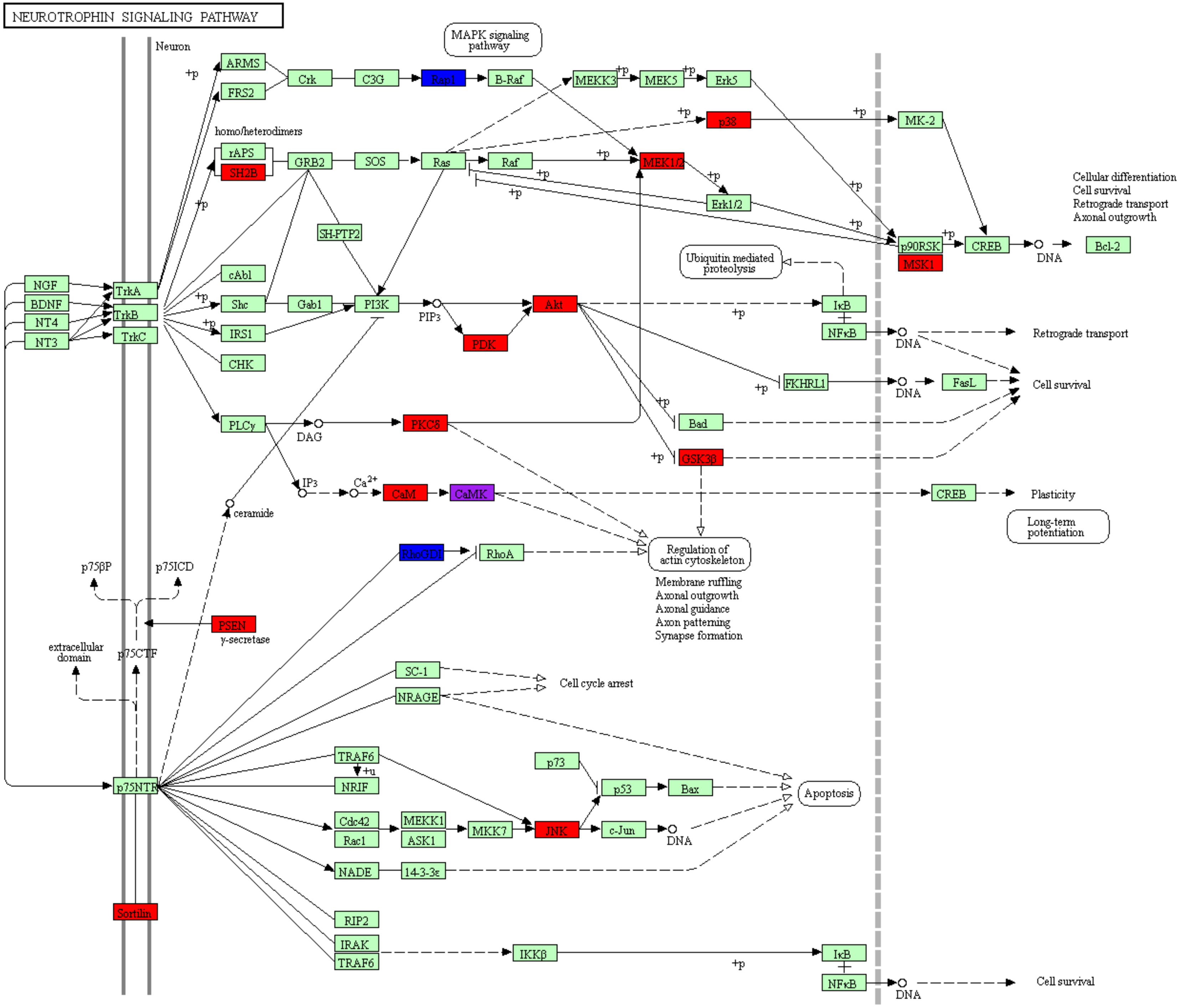
NEUROTROPHIN SIGNALING PATHWAY
Neuron
MAPK signaling pathway
ARMS
FRS2
Crk
C3G
Rap1
B-Raf
MEKK3
MEK5
Erk5
p38
MK-2
homo/heterodimers
rAPS
SH2B
GRB2
SOS
Ras
Raf
MEK1/2
Erk1/2
p90RSK
MSK1
CREB
DNA
Bcl-2
Cellular differentiation
Cell survival
Retrograde transport
Axonal outgrowth
SH-PTP2
Ubiquitin mediated proteolysis
NGF
BDNF
NT4
NT3
TrkA
TrkB
TrkC
cAbl
Shc
Gab1
PI3K
PIP3
PDK
Akt
IκB
NFκB
Retrograde transport
IRS1
CHK
FKHRL1
FasL
Cell survival
PLCγ
DAG
PKCδ
Bad
GSK3β
IP3
Ca2+
CaM
CaMK
CREB
Plasticity
Long-term potentiation
ceramide
RhoGDI
RhoA
Regulation of actin cytoskeleton
Membrane ruffling
Axonal outgrowth
Axonal guidance
Axon patterning
Synapse formation
p75βP
p75ICD
PSEN
γ-secretase
extracellular domain
p75CTF
SC-1
NRAGE
Cell cycle arrest
TRAF6
NRIF
p75NTR
p73
p53
Bax
Apoptosis
Cdc42
Rac1
MEKK1
ASK1
MKK7
JNK
c-Jun
NADE
14-3-3ε
Sortilin
RIP2
IRAK
TRAF6
IKKβ
IκB
NFκB
Cell survival

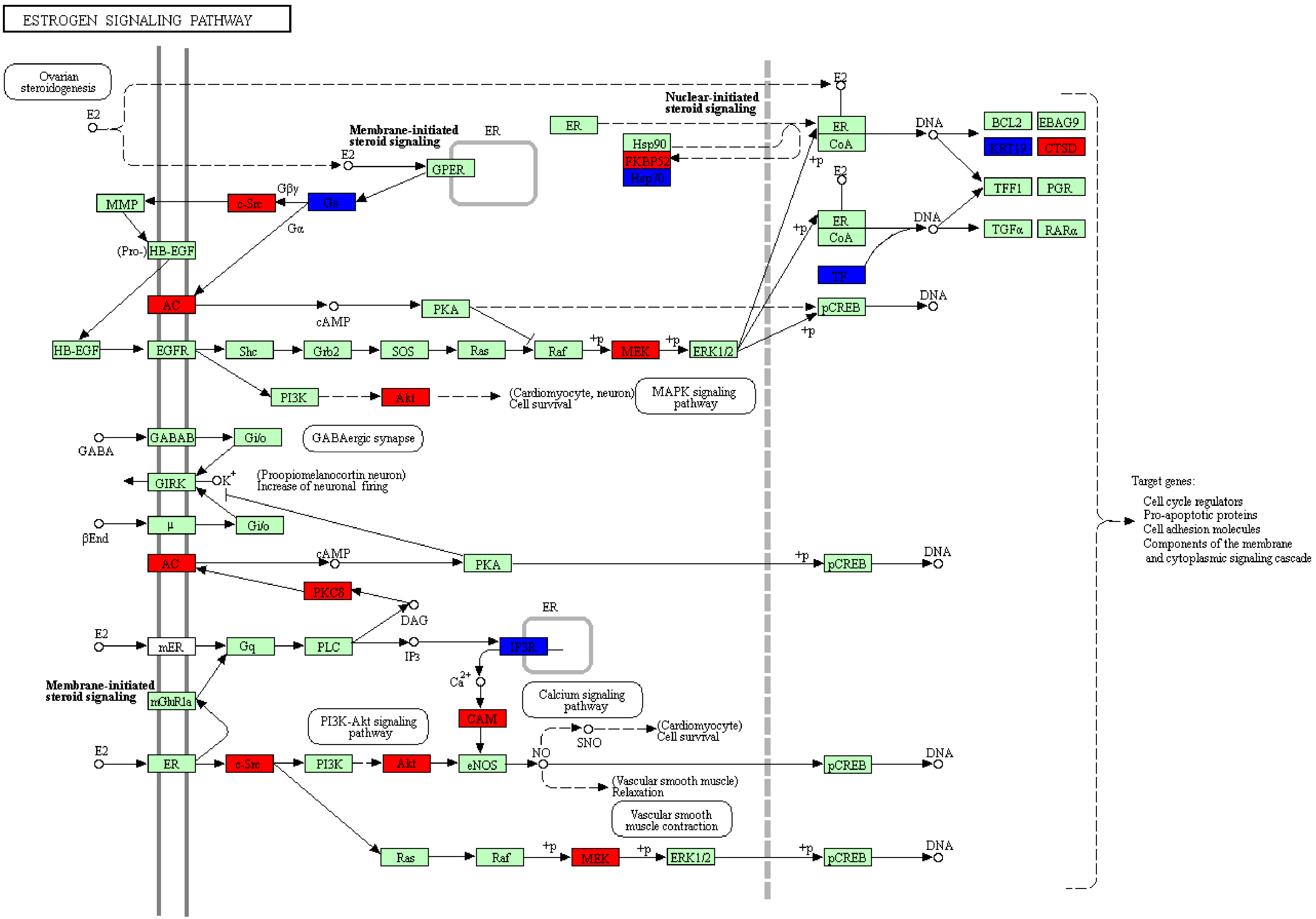
ESTROGEN SIGNALING PATHWAY
Ovarian steroidogenesis
E2
Membrane-initiated steroid signaling
ER
GPER
Gβγ
Gα
MMP
c-Src
Gs
(Pro-)HB-EGF
HB-EGF
AC
cAMP
PKA
EGFR
Shc
Grb2
SOS
Ras
Raf
+p
MEK
ERK1/2
PI3K
Akt
(Cardiomyocyte, neuron)
Cell survival
MAPK signaling pathway
GABA
GABAB
Gi/o
GABAergic synapse
GIRK
K+
(Proopiomelanocortin neuron)
Increase of neuronal firing
βEnd
μ
PKCδ
DAG
mER
Gq
PLC
IP3
IP3R
Ca2+
CAM
Calcium signaling pathway
mGluR1a
PI3K-Akt signaling pathway
eNOS
NO
SNO
(Cardiomyocyte)
Cell survival
(Vascular smooth muscle)
Relaxation
Vascular smooth muscle contraction
Nuclear-initiated steroid signaling
Hsp90
FKBP52
Hsp70
CoA
TF
DNA
pCREB
BCL2
EBAG9
KRT19
CTSD
TFF1
PGR
TGFα
RARα
Target genes:
Cell cycle regulators
Pro-apoptotic proteins
Cell adhesion molecules
Components of the membrane and cytoplasmic signaling cascade

DILATED CARDIOMYOPATHY (DCM)

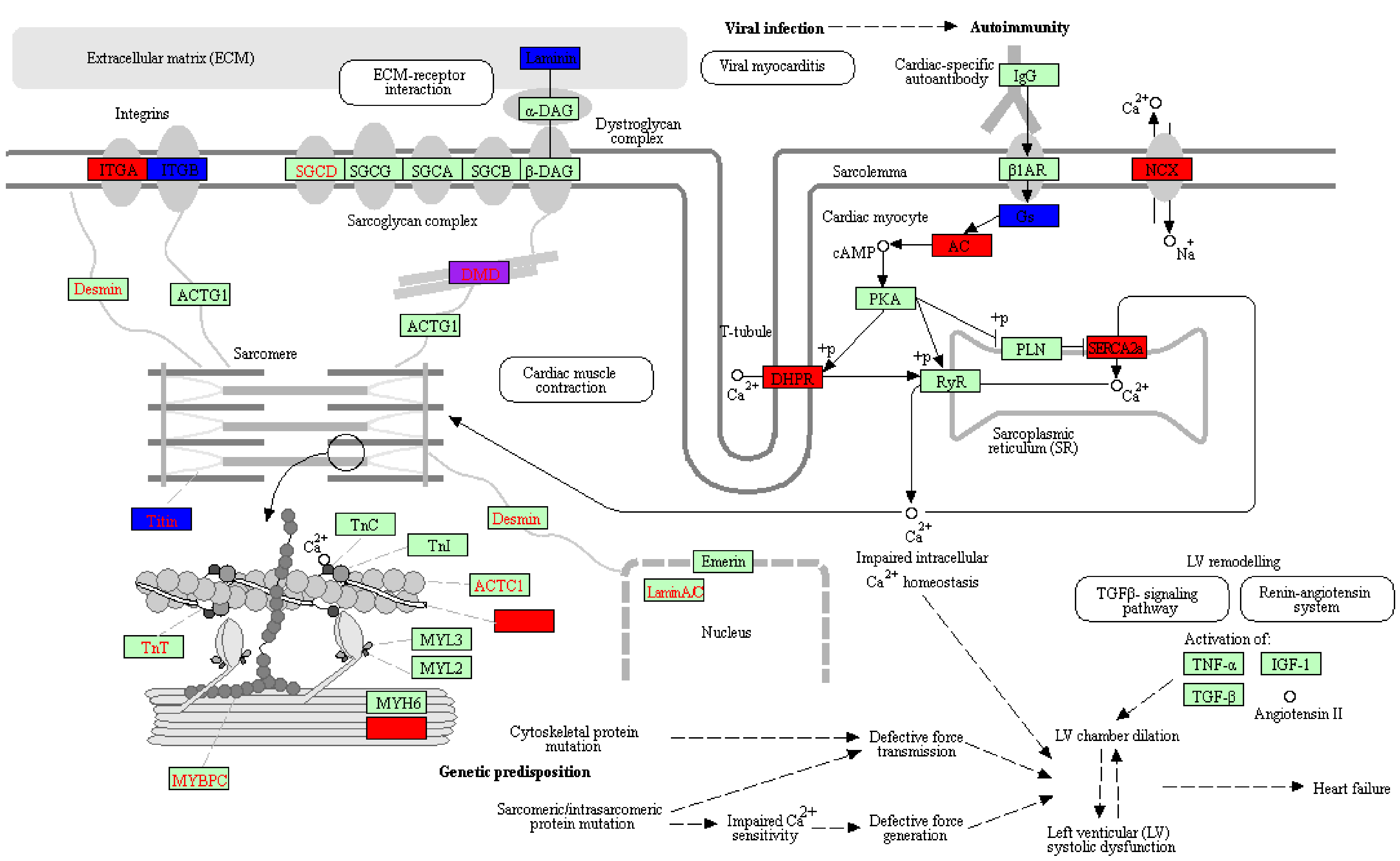

HYPERTROPHIC CARDIOMYOPATHY (HCM)

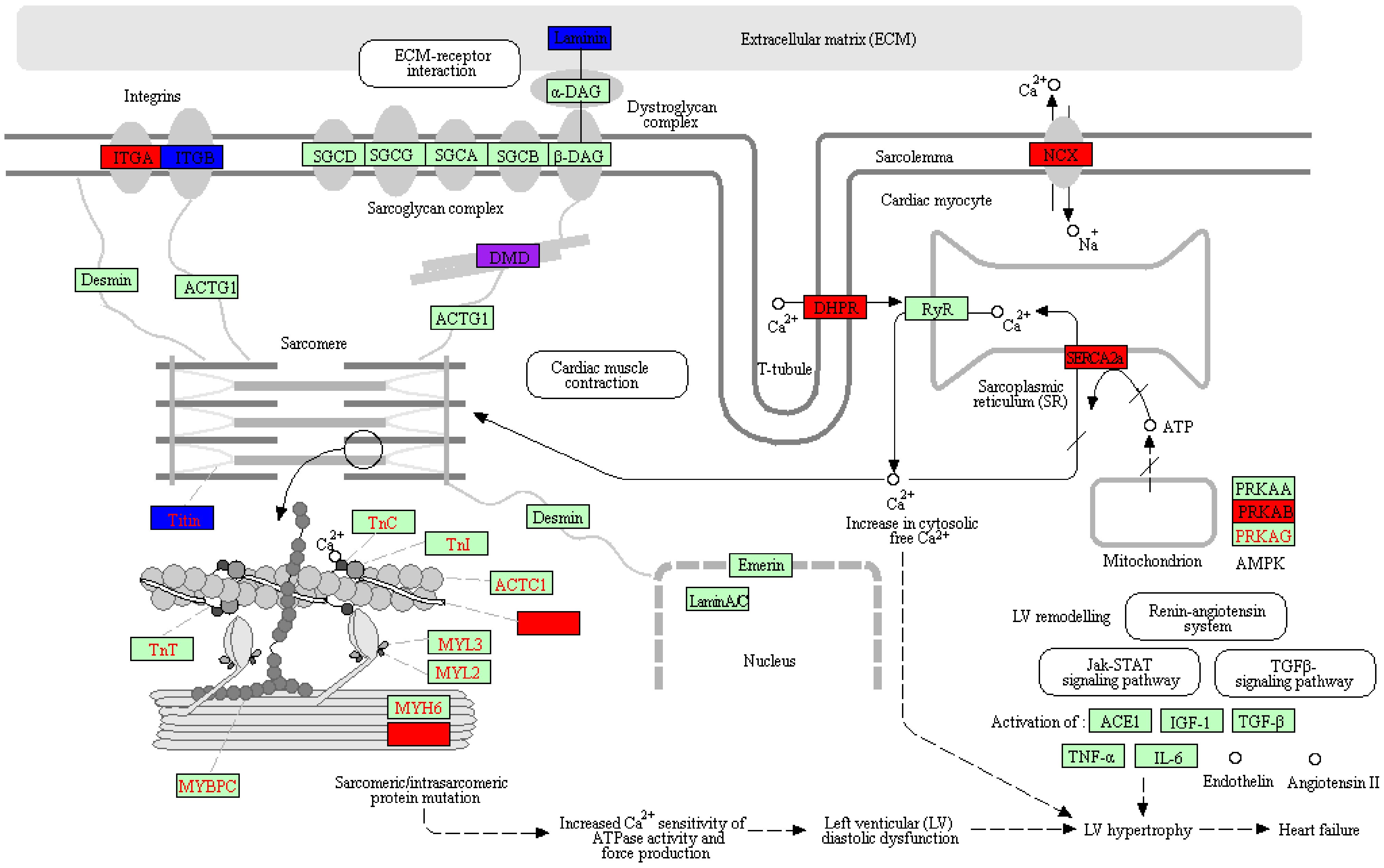

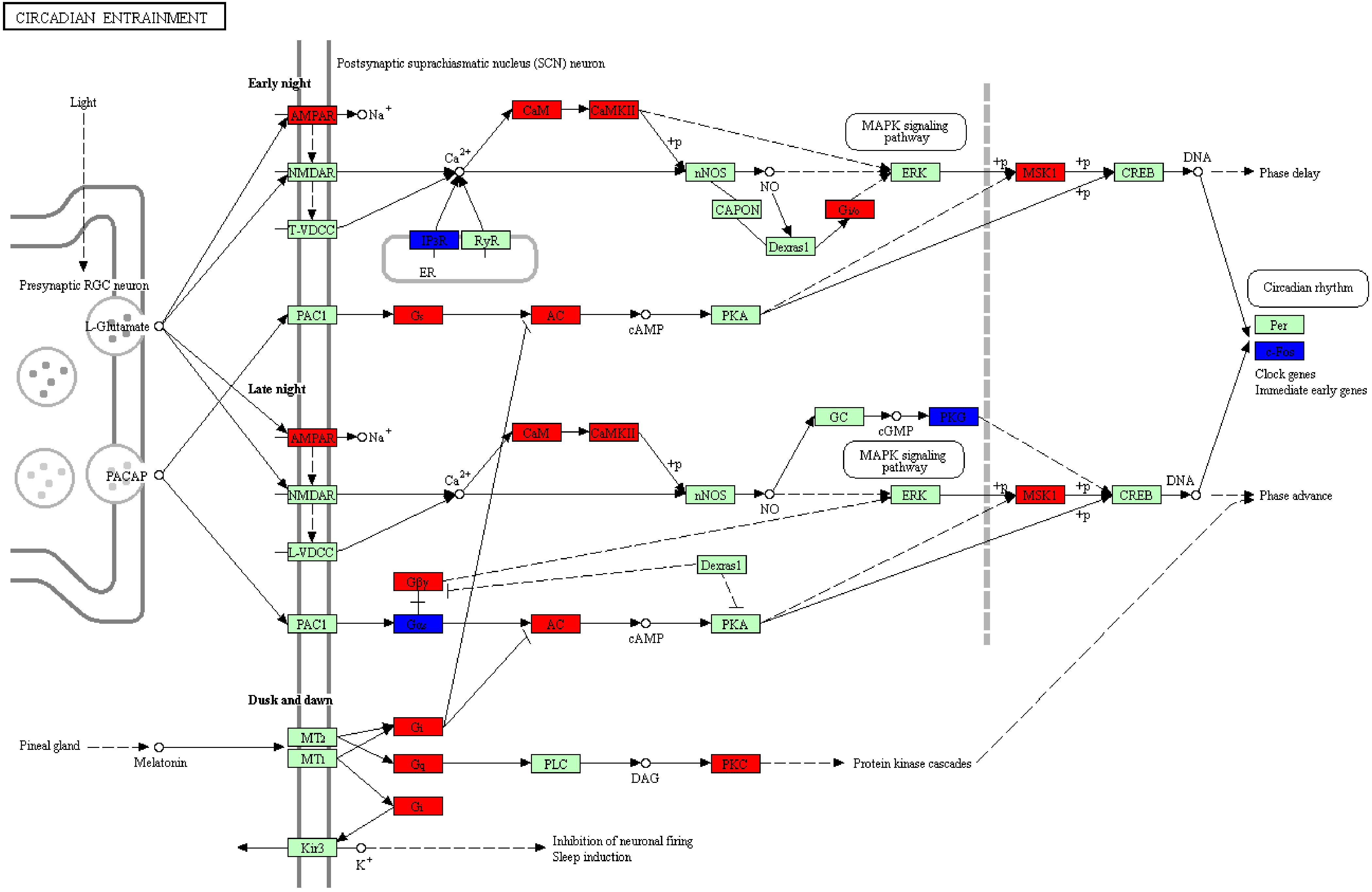

CIRCADIAN ENTRAINMENT
Postsynaptic suprachiasmatic nucleus (SCN) neuron
Early night
Light
AMPAR
Na+
CaM
CaMKII
MAPK signaling pathway
NMDAR
Ca2+
+p
nNOS
NO
ERK
MSK1
CREB
DNA
Phase delay
T-VDCC
CAPON
Gi/o
IP3R
RyR
Dexras1
ER
Presynaptic RGC neuron
Circadian rhythm
L-Glutamate
PAC1
Gs
AC
cAMP
PKA
Per
c-Fos
Clock genes
Immediate early genes
Late night
GC
cGMP
PKG
PACAP
Phase advance
L-VDCC
Gβγ
Gαs
Dusk and dawn
Pineal gland
Melatonin
MT2
MT1
Gi
Gq
PLC
DAG
PKC
Protein kinase cascades
Kir3
K+
Inhibition of neuronal firing
Sleep induction

GLUCAGON SIGNALING PATHWAY

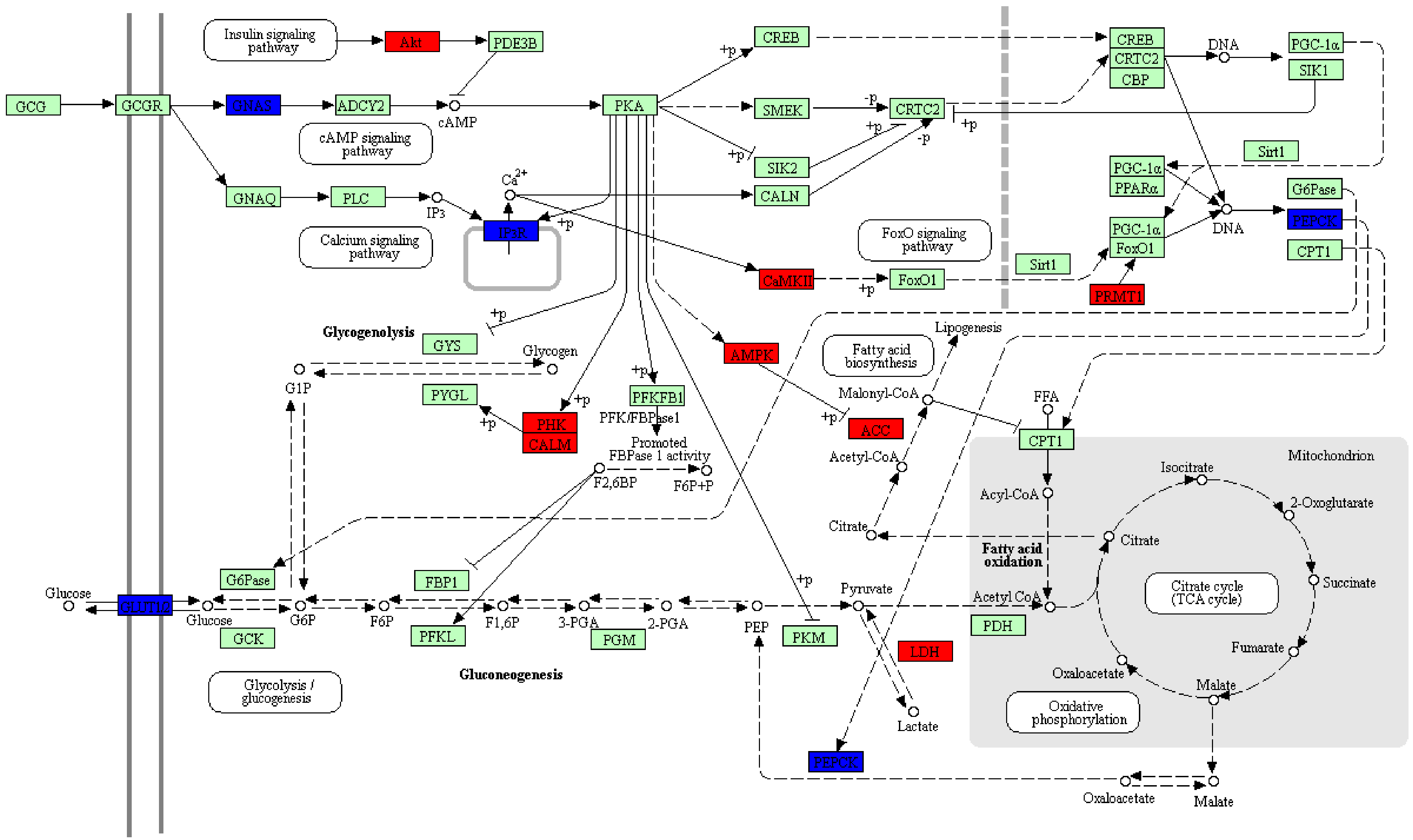

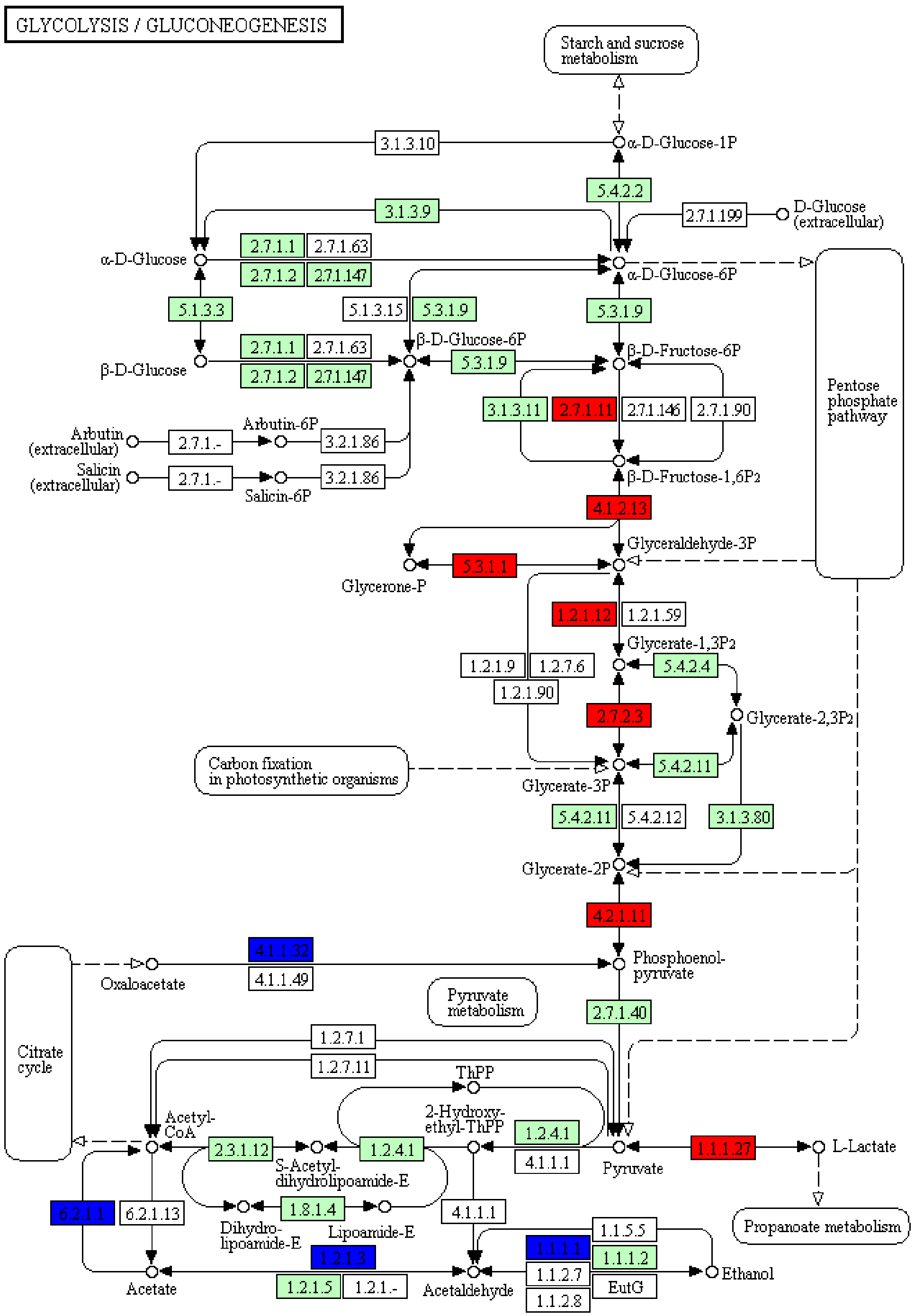
GLYCOLYSIS / GLUCONEOGENESIS
Starch and sucrose metabolism
α-D-Glucose-1P
3.1.3.10
5.4.2.2
3.1.3.9
2.7.1.199
D-Glucose (extracellular)
α-D-Glucose
2.7.1.1
2.7.1.63
2.7.1.2
2.7.1.147
α-D-Glucose-6P
5.1.3.3
5.1.3.15
5.3.1.9
β-D-Glucose-6P
β-D-Fructose-6P
β-D-Glucose
Pentose phosphate pathway
3.1.3.11
2.7.1.11
2.7.1.146
2.7.1.90
Arbutin (extracellular)
2.7.1.-
Arbutin-6P
3.2.1.86
Salicin (extracellular)
Salicin-6P
β-D-Fructose-1,6P2
4.1.2.13
Glyceraldehyde-3P
Glycerone-P
5.3.1.1
1.2.1.12
1.2.1.59
Glycerate-1,3P2
1.2.1.9
1.2.7.6
5.4.2.4
1.2.1.90
2.7.2.3
Glycerate-2,3P2
Carbon fixation in photosynthetic organisms
5.4.2.11
Glycerate-3P
5.4.2.12
3.1.3.80
Glycerate-2P
4.2.1.11
4.1.1.32
Oxaloacetate
4.1.1.49
Phosphoenol-pyruvate
Citrate cycle
Pyruvate metabolism
2.7.1.40
1.2.7.1
1.2.7.11
ThPP
Acetyl-CoA
2-Hydroxy-ethyl-ThPP
1.2.4.1
2.3.1.12
4.1.1.1
Pyruvate
1.1.1.27
L-Lactate
S-Acetyl-dihydrolipoamide-E
6.2.1.1
6.2.1.13
1.8.1.4
Dihydro-lipoamide-E
Lipoamide-E
Propanoate metabolism
1.1.5.5
1.1.1.1
1.2.1.3
1.1.1.2
Acetate
1.2.1.5
1.2.1.-
Acetaldehyde
1.1.2.7
1.1.2.8
EutG
Ethanol

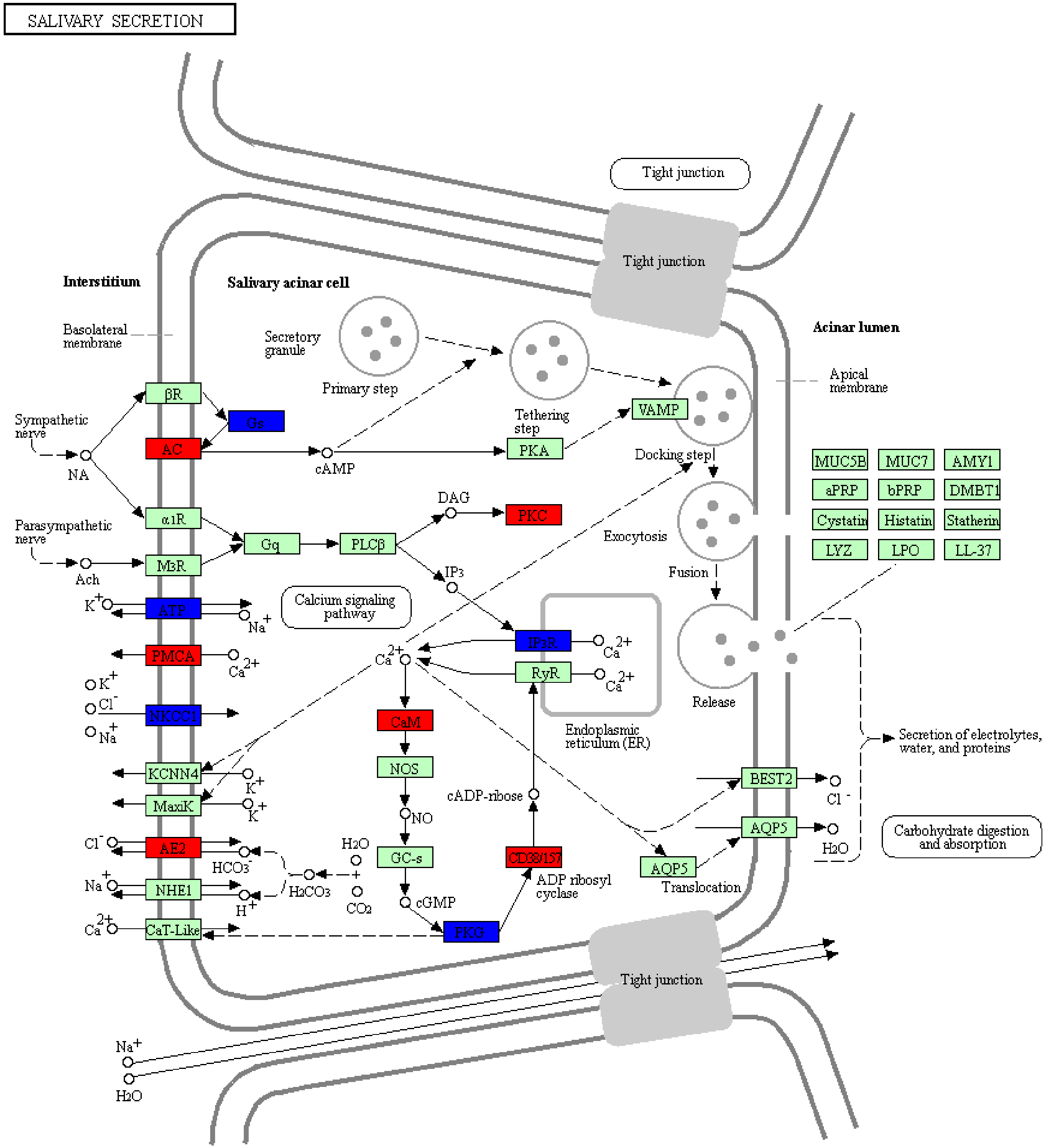

SALIVARY SECRETION
Tight junction
Tight junction
Interstitium
Salivary acinar cell
Acinar lumen
Basolateral membrane
Secretory granule
Primary step
Tethering step
Docking step
Apical membrane
Sympathetic nerve
NA
βR
Gs
AC
cAMP
PKA
VAMP
MUC5B
MUC7
AMY1
aPRP
bPRP
DMBT1
Cystatin
Histatin
Statherin
LYZ
LPO
LL-37
Parasympathetic nerve
α1R
M3R
Ach
Gq
PLCβ
DAG
PKC
IP3
Exocytosis
Fusion
K+
ATP
Na+
Calcium signaling pathway
PMCA
Ca2+
IP3R
RyR
Endoplasmic reticulum (ER)
Release
K+
Cl-
Na+
NKCC1
CaM
NOS
NO
GC-s
cGMP
PKG
cADP-ribose
CD38/157
ADP ribosyl cyclase
KCNN4
MaxiK
Cl-
AE2
HCO3-
H2CO3
H2O
CO2
Na+
NHE1
H+
CaT-Like
BEST2
AQP5
AQP5
Translocation
Cl-
H2O
Secretion of electrolytes, water, and proteins
Carbohydrate digestion and absorption
Tight junction
Na+
H2O

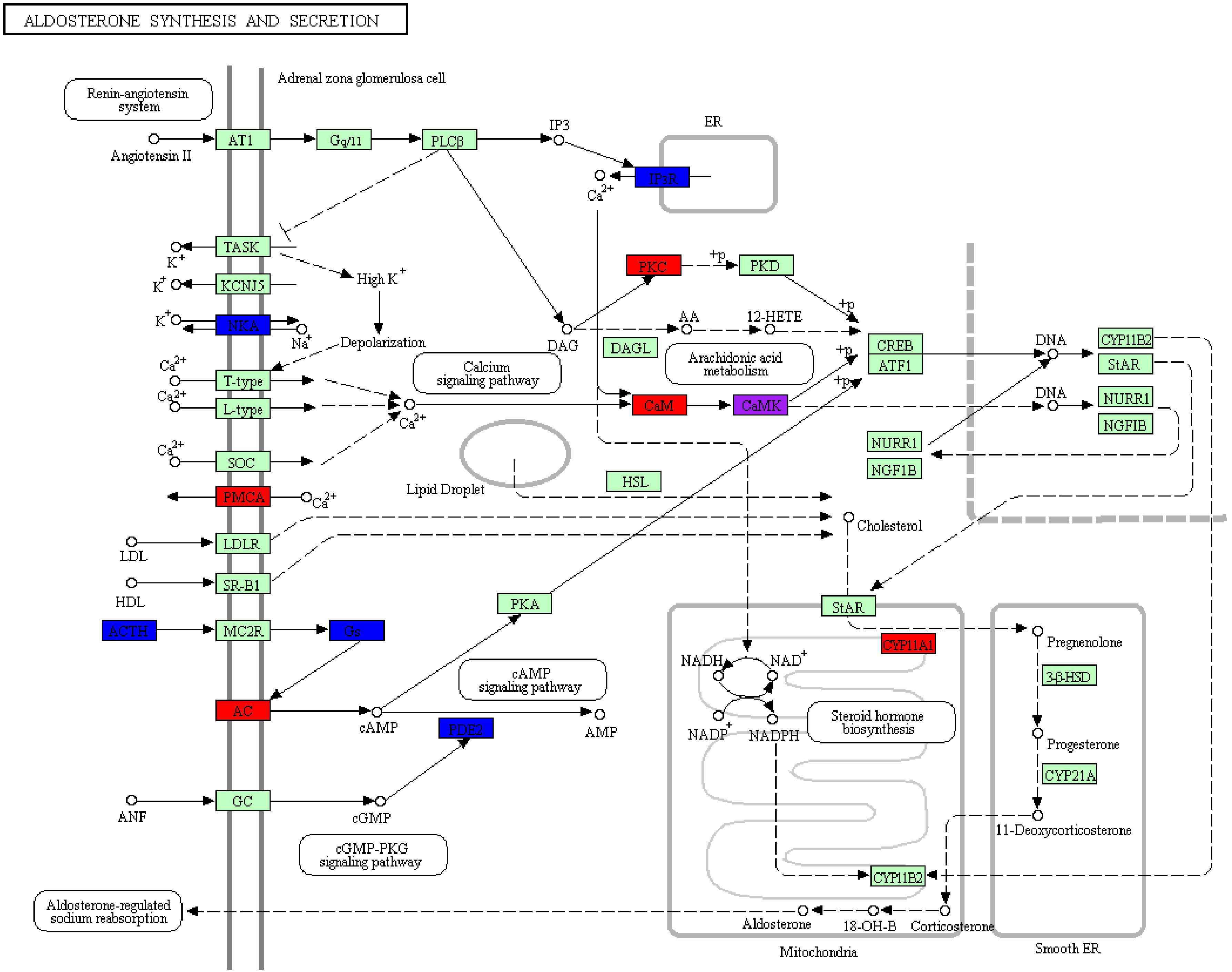

ALDOSTERONE SYNTHESIS AND SECRETION
Adrenal zona glomerulosa cell
Renin-angiotensin system
Angiotensin II
AT1
Gq/11
PLCβ
IP3
ER
IP3R
Ca2+
TASK
K+
High K+
KCNJ5
K+
NKA
K+
Na+
Depolarization
DAG
PKC
+p
PKD
+p
AA
12-HETE
DAGL
Arachidonic acid metabolism
Calcium signaling pathway
Ca2+
T-type
Ca2+
L-type
Ca2+
CaM
CaMK
+p
+p
CREB
ATF1
DNA
CYP11B2
StAR
DNA
NURR1
NGFIB
NURR1
NGF1B
Ca2+
SOC
Lipid Droplet
HSL
PMCA
Ca2+
LDLR
LDL
Cholesterol
SR-B1
HDL
PKA
StAR
ACTH
MC2R
Gs
CYP11A1
Pregnenolone
NADH
NAD+
3β-HSD
cAMP signaling pathway
AC
cAMP
PDE2
AMP
NADP+
NADPH
Steroid hormone biosynthesis
Progesterone
CYP21A
ANF
GC
cGMP
11-Deoxycorticosterone
cGMP-PKG signaling pathway
CYP11B2
Aldosterone-regulated sodium reabsorption
Aldosterone
18-OH-B
Corticosterone
Mitochondria
Smooth ER

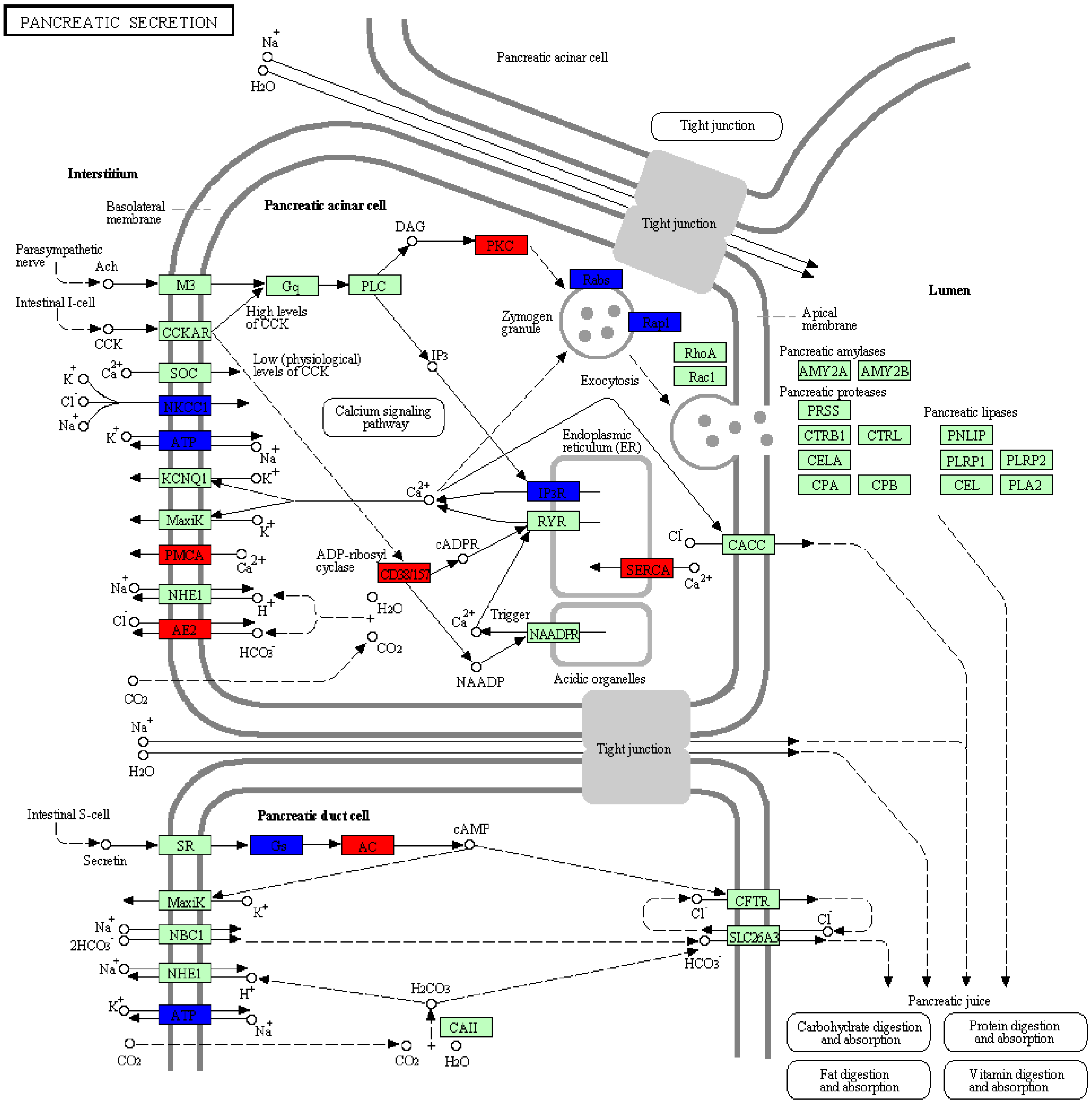

PANCREATIC SECRETION
Pancreatic acinar cell
Tight junction
Interstitium
Basolateral membrane
Pancreatic acinar cell
Parasympathetic nerve
Ach
M3
Gq
PLC
DAG
PKC
Rabs
Rap1
Intestinal I-cell
CCK
CCKAR
High levels of CCK
Low (physiological) levels of CCK
Zymogen granule
IP3
RhoA
Rac1
Exocytosis
SOC
NKCC1
ATP
KCNQ1
MaxiK
PMCA
NHE1
AE2
Calcium signaling pathway
Endoplasmic reticulum (ER)
IP3R
RYR
cADPR
ADP-ribosyl cyclase
CD38/157
SERCA
CACC
Trigger
NAADPR
NAADP
Acidic organelles
H2O
CO2
HCO3-
Lumen
Apical membrane
Pancreatic amylases
AMY2A
AMY2B
Pancreatic proteases
PRSS
CTRB1
CTRL
CELA
CPA
CPB
Pancreatic lipases
PNLIP
PLRP1
PLRP2
CEL
PLA2
Na+
H2O
Tight junction
Intestinal S-cell
Secretin
Pancreatic duct cell
SR
Gs
AC
cAMP
MaxiK
NBC1
NHE1
ATP
CFTR
SLC26A3
H2CO3
CAII
Pancreatic juice
Carbohydrate digestion and absorption
Protein digestion and absorption
Fat digestion and absorption
Vitamin digestion and absorption

INFLAMMATORY MEDIATOR REGULATION OF TRP CHANNELS

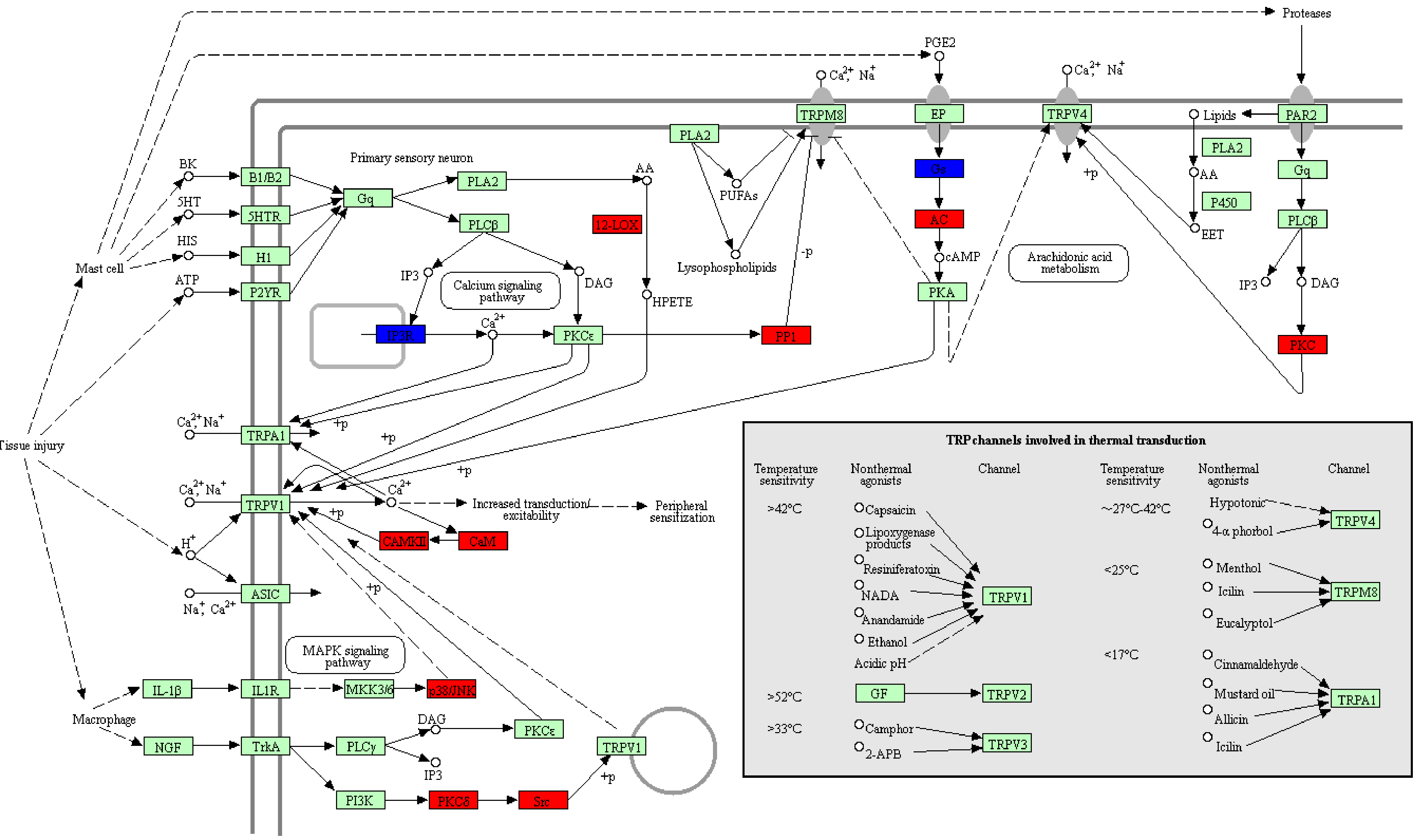

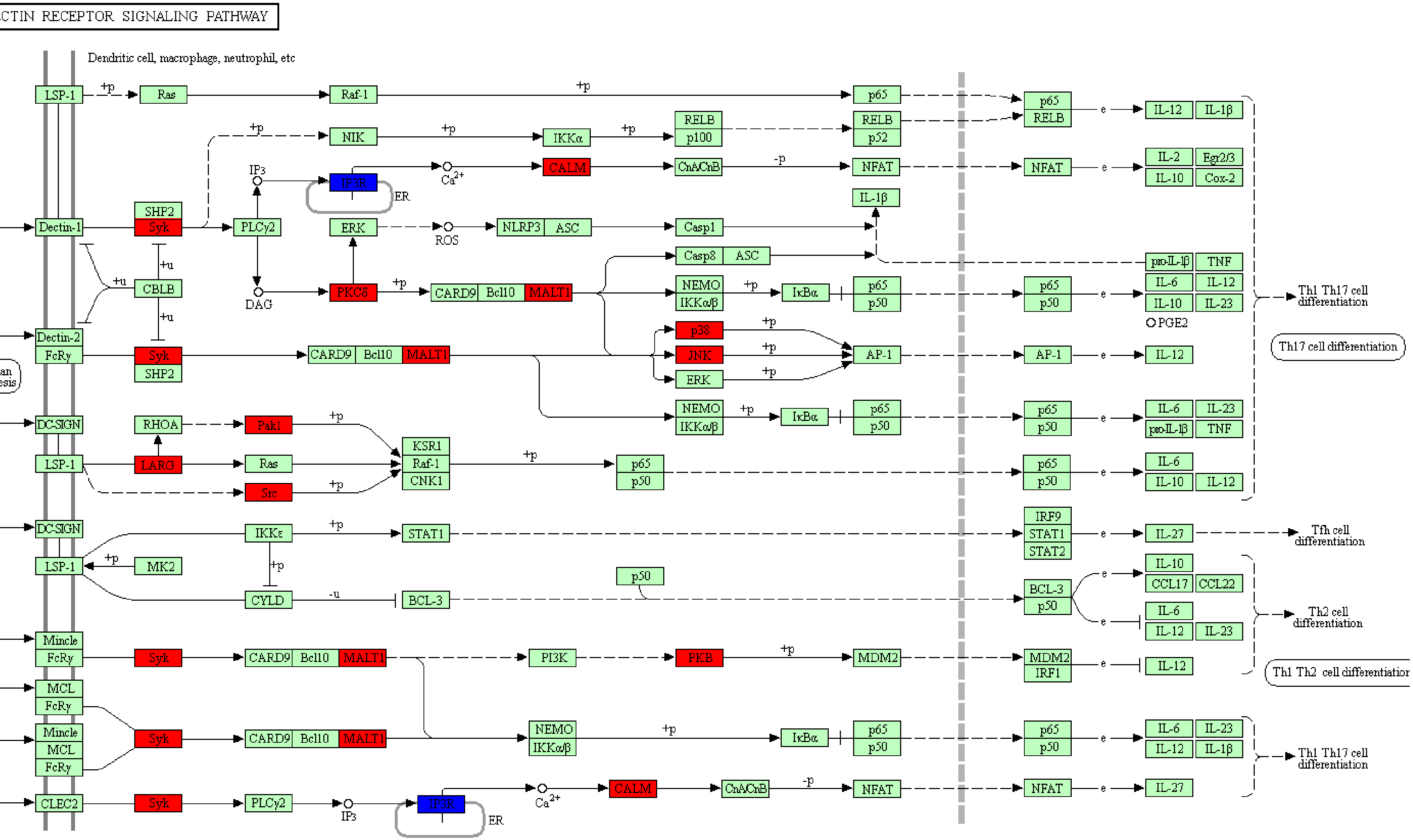

CTIN RECEPTOR SIGNALING PATHWAY
Dendritic cell, macrophage, neutrophil, etc
LSP-1
Ras
Raf-1
+p
p65
RELB
p65 RELB
IL-12
IL-1β
NIK
IKKα
RELB p100
RELB p52
IP3
IP3R
ER
Ca2+
CALM
CnA/CnB
-p
NFAT
IL-2
Egr2/3
IL-10
Cox-2
IL-1β
Dectin-1
SHP2
Syk
PLCγ2
ERK
ROS
NLRP3
ASC
Casp1
Casp8
ASC
pro-IL-1β
TNF
+u
CBLB
DAG
PKCδ
CARD9
Bcl10
MALT1
NEMO IKKα/β
IκBα
p65 p50
IL-6
IL-23
PGE2
Th1 Th17 cell differentiation
Dectin-2
FcRγ
p38
JNK
AP-1
Th17 cell differentiation
an
esis
DC-SIGN
RHOA
Pak1
KSR1 Raf-1 CNK1
LARG
Src
IRF9 STAT1 STAT2
IKKε
STAT1
IL-27
Tfh cell differentiation
MK2
CYLD
-u
BCL-3
p50
BCL-3 p50
CCL17
CCL22
Th2 cell differentiation
Mincle
PI3K
PKB
MDM2
MDM2 IRF1
Th1 Th2 cell differentiatior
MCL
CLEC2
e

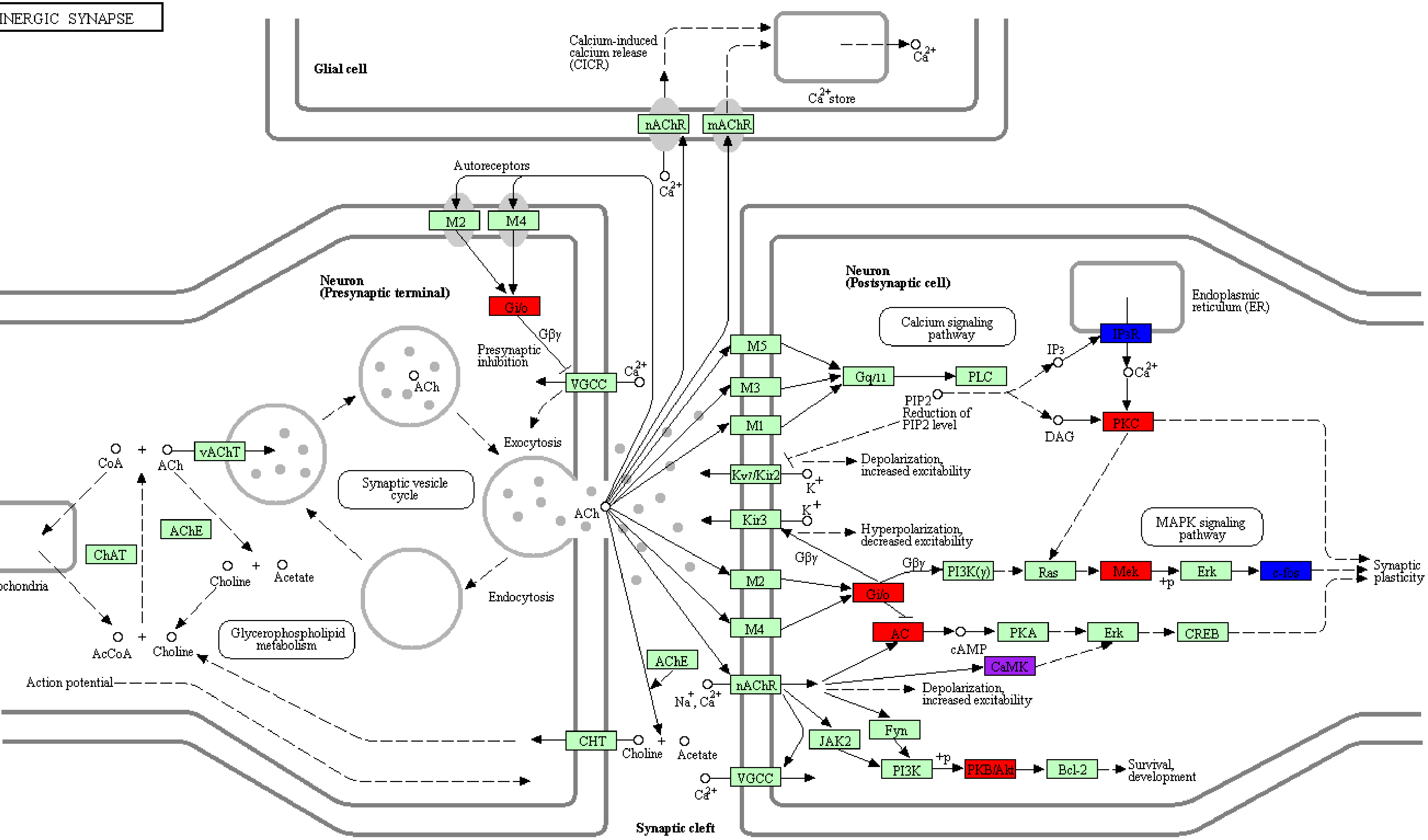
INERGIC SYNAPSE
Glial cell
Calcium-induced calcium release (CICR)
Ca2+ store
Ca2+
nAChR
mAChR
Autoreceptors
M2
M4
Neuron (Presynaptic terminal)
Gi/o
Gβγ
Presynaptic inhibition
VGCC
ACh
Exocytosis
CoA
vAChT
Synaptic vesicle cycle
AChE
ChAT
Choline
Acetate
Endocytosis
AcCoA
Glycerophospholipid metabolism
chondria
Action potential
CHT
Na+, Ca2+
Synaptic cleft
Neuron (Postsynaptic cell)
Calcium signaling pathway
Endoplasmic reticulum (ER)
IP3R
IP3
M5
M3
M1
Gq/11
PLC
PIP2
Reduction of PIP2 level
DAG
PKC
Kv7/Kir2
K+
Depolarization, increased excitability
Kir3
Hyperpolarization, decreased excitability
MAPK signaling pathway
PI3K(γ)
Ras
Mek
Erk
c-fos
+p
Synaptic plasticity
AC
cAMP
PKA
CREB
CaMK
JAK2
Fyn
PI3K
PKB/Akt
Bcl-2
Survival, development

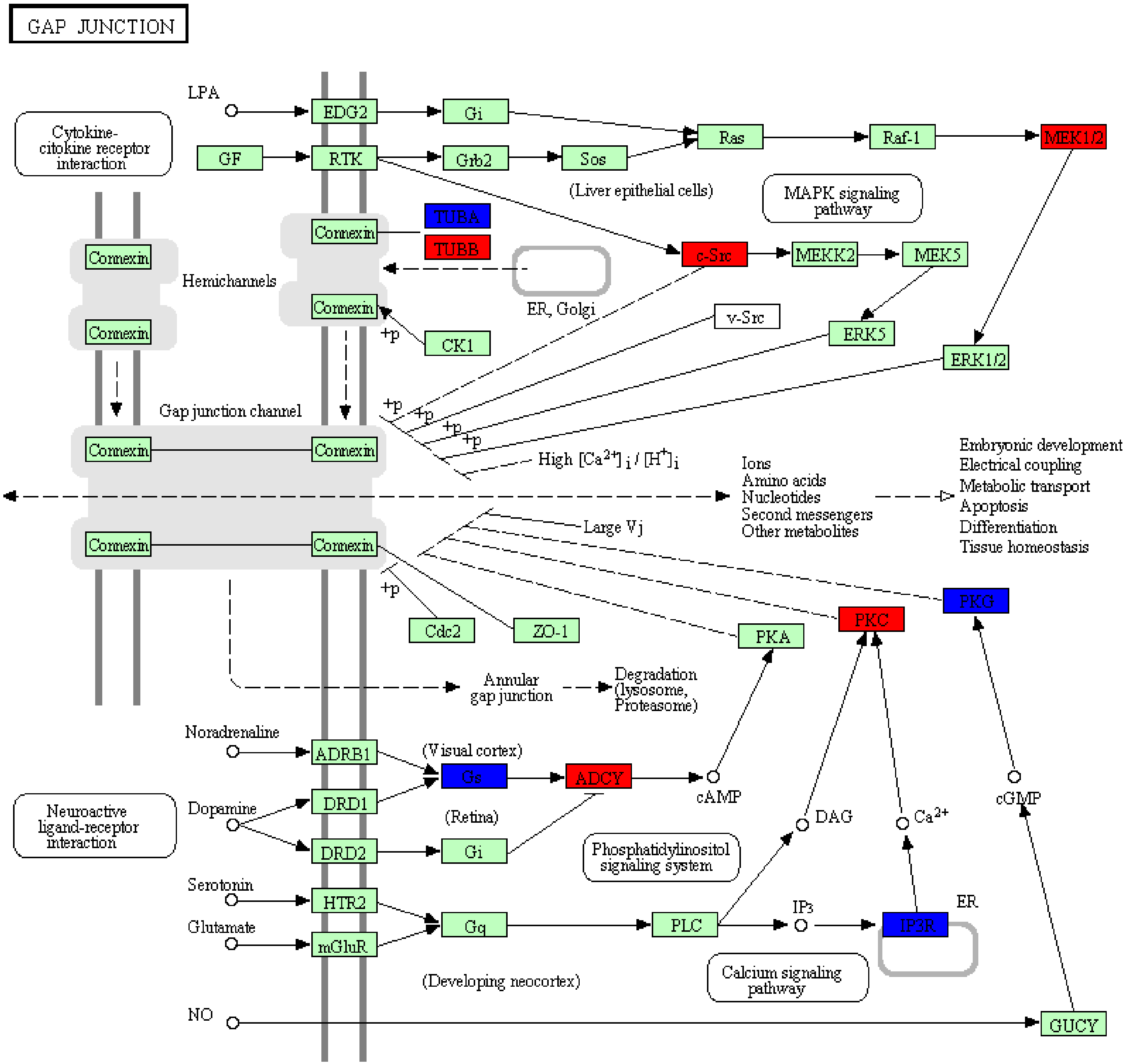
GAP JUNCTION
LPA
EDG2
Gi
Ras
Raf-1
MEK1/2
Cytokine-citokine receptor interaction
GF
RTK
Grb2
Sos
(Liver epithelial cells)
MAPK signaling pathway
Connexin
TUBA
TUBB
c-Src
MEKK2
MEK5
Hemichannels
ER, Golgi
v-Src
ERK5
ERK1/2
CK1
+p
Gap junction channel
High [Ca2+]i / [H+]i
Ions
Amino acids
Nucleotides
Second messengers
Other metabolites
Embryonic development
Electrical coupling
Metabolic transport
Apoptosis
Differentiation
Tissue homeostasis
Large Vj
PKG
PKC
PKA
Cdc2
ZO-1
Annular gap junction
Degradation (lysosome, Proteasome)
Noradrenaline
ADRB1
(Visual cortex)
Gs
ADCY
cAMP
Neuroactive ligand-receptor interaction
Dopamine
DRD1
DRD2
(Retina)
DAG
Ca2+
cGMP
Phosphatidylinositol signaling system
Serotonin
HTR2
Glutamate
mGluR
Gq
PLC
IP3
IP3R
ER
Calcium signaling pathway
(Developing neocortex)
NO
GUCY

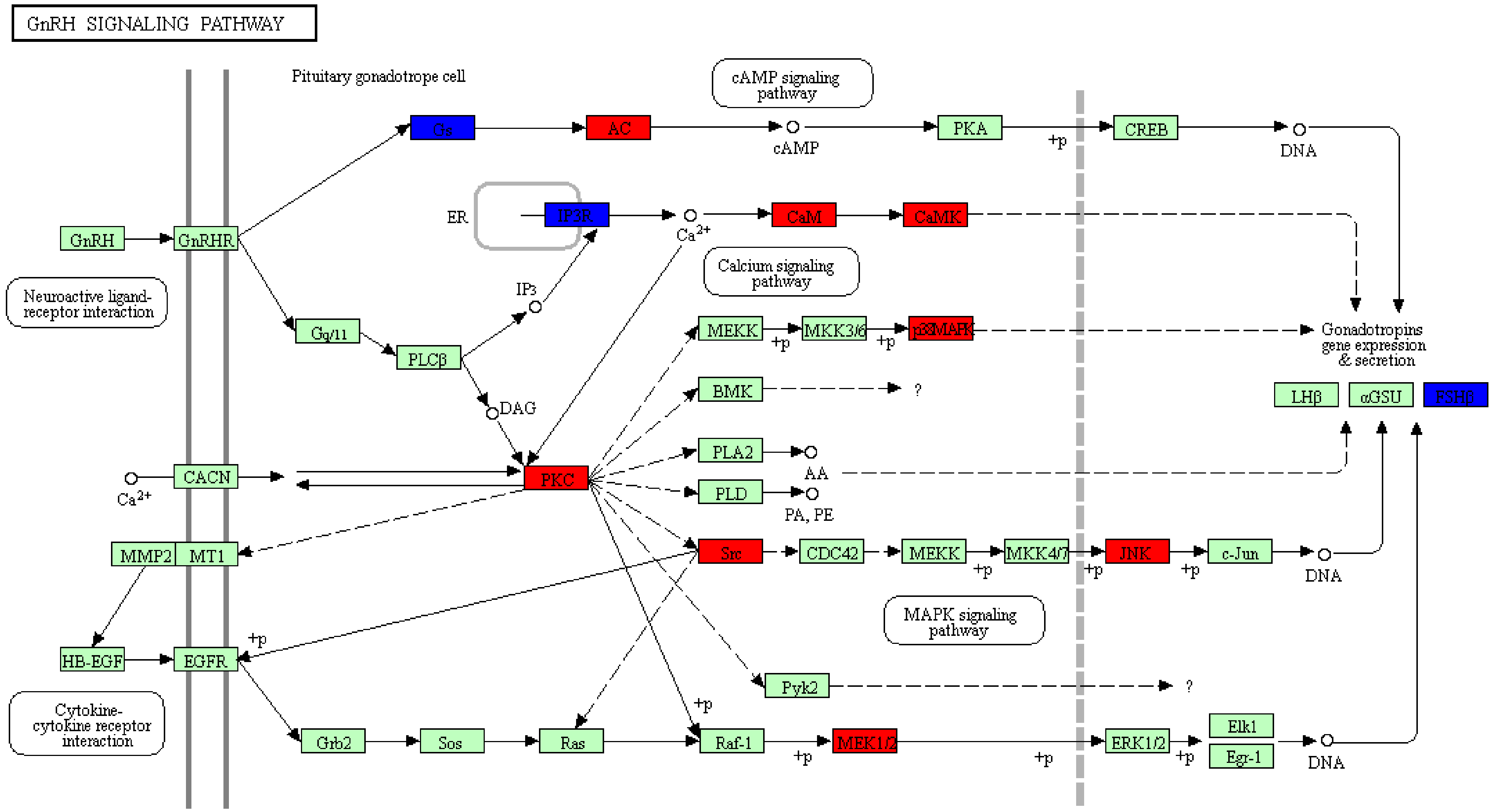
GnRH SIGNALING PATHWAY
Pituitary gonadotrope cell
cAMP signaling pathway
Gs
AC
cAMP
PKA
+p
CREB
DNA
ER
IP3R
Ca2+
CaM
CaMK
GnRH
GnRHR
Calcium signaling pathway
Neuroactive ligand-receptor interaction
IP3
Gq/11
PLCβ
MEKK
MKK3/6
p38MAPK
Gonadotropins gene expression & secretion
BMK
?
DAG
LHβ
αGSU
FSHβ
PLA2
AA
CACN
PKC
PLD
PA, PE
Src
CDC42
MEKK
MKK4/7
JNK
c-Jun
DNA
MMP2
MT1
MAPK signaling pathway
HB-EGF
EGFR
Pyk2
Cytokine-cytokine receptor interaction
Grb2
Sos
Ras
Raf-1
MEK1/2
ERK1/2
Elk1
Egr-1
DNA

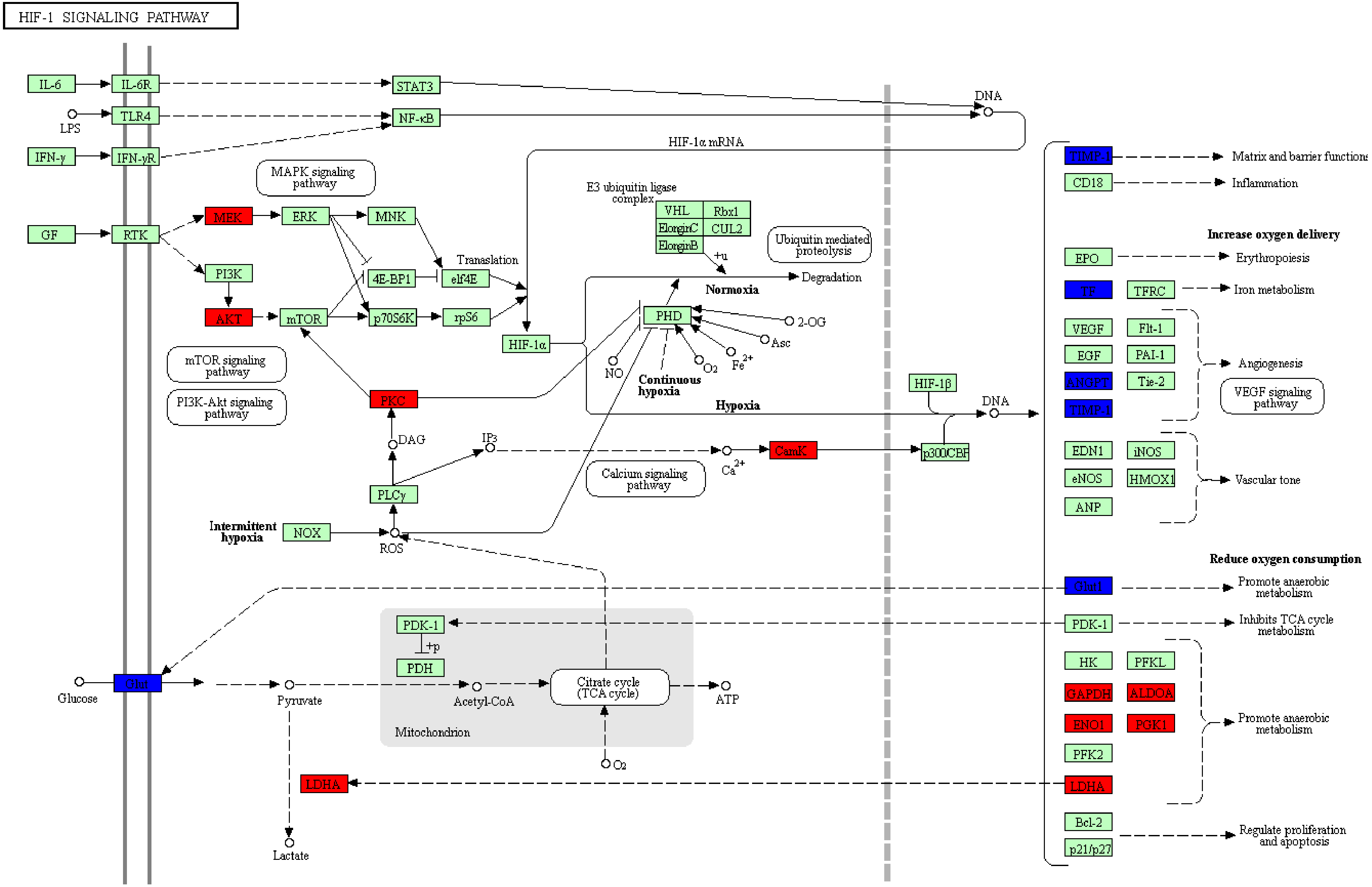
HIF-1 SIGNALING PATHWAY
IL-6
IL-6R
STAT3
LPS
TLR4
NF-κB
IFN-γ
IFN-γR
DNA
HIF-1α mRNA
MAPK signaling pathway
GF
RTK
MEK
ERK
MNK
PI3K
4E-BP1
eIF4E
Translation
AKT
mTOR
p70S6K
rpS6
HIF-1α
E3 ubiquitin ligase complex
VHL
Rbx1
ElonginC
CUL2
ElonginB
+u
Ubiquitin mediated proteolysis
Degradation
Normoxia
PHD
2-OG
Asc
NO
O2
Fe2+
Continuous hypoxia
Hypoxia
mTOR signaling pathway
PI3K-Akt signaling pathway
PKC
DAG
IP3
PLCγ
CamK
Ca2+
Calcium signaling pathway
HIF-1β
DNA
p300/CBP
Intermittent hypoxia
NOX
ROS
Glucose
Glut
Pyruvate
PDK-1
+p
PDH
Acetyl-CoA
Citrate cycle (TCA cycle)
ATP
Mitochondrion
O2
LDHA
Lactate
TIMP-1
Matrix and barrier functions
CD18
Inflammation
Increase oxygen delivery
EPO
Erythropoiesis
TF
TFRC
Iron metabolism
VEGF
Flt-1
EGF
PAI-1
ANGPT
Tie-2
Angiogenesis
TIMP-1
VEGF signaling pathway
EDN1
iNOS
eNOS
HMOX1
ANP
Vascular tone
Reduce oxygen consumption
Glut1
Promote anaerobic metabolism
PDK-1
Inhibits TCA cycle metabolism
HK
PFKL
GAPDH
ALDOA
ENO1
PGK1
PFK2
LDHA
Promote anaerobic metabolism
Bcl-2
p21/p27
Regulate proliferation and apoptosis

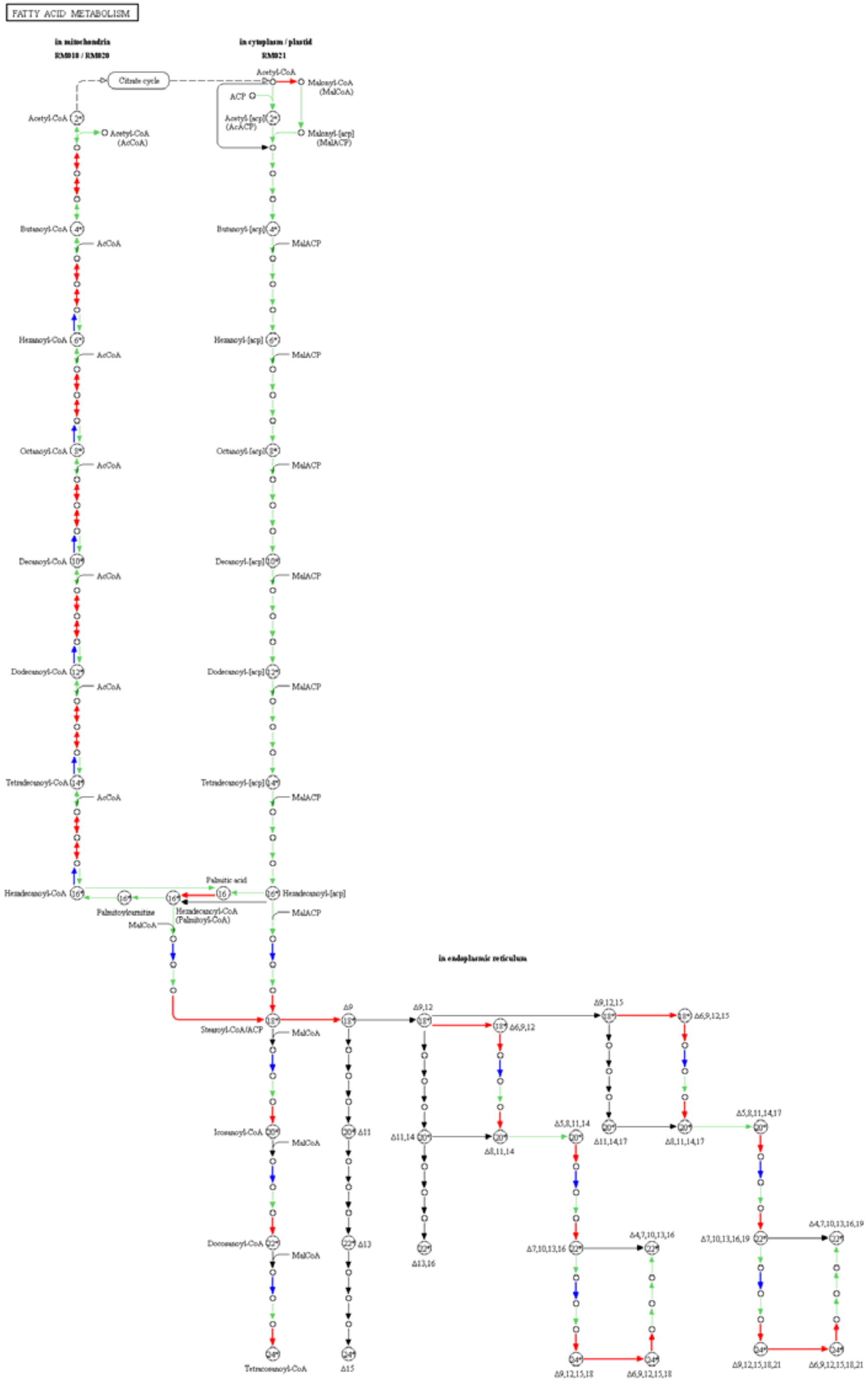
FATTY ACID METABOLISM
in mitochondria
RM018 / RM020
in cytoplasm / plastid
RM021
Citrate cycle
Acetyl-CoA
Malonyl-CoA (MalCoA)
ACP
Acetyl-[acp] (AcACP)
Malonyl-[acp] (MalACP)
Acetyl-CoA (AcCoA)
Butanoyl-CoA
Butanoyl-[acp]
AcCoA
MalACP
Hexanoyl-CoA
Hexanoyl-[acp]
Octanoyl-CoA
Octanoyl-[acp]
Decanoyl-CoA
Decanoyl-[acp]
Dodecanoyl-CoA
Dodecanoyl-[acp]
Tetradecanoyl-CoA
Tetradecanoyl-[acp]
Palmitic acid
Hexadecanoyl-CoA
Hexadecanoyl-[acp]
Palmitoylcarnitine
Hexadecanoyl-CoA (Palmitoyl-CoA)
MalCoA
in endoplasmic reticulum
Stearoyl-CoA/ACP
Δ9
Δ9,12
Δ6,9,12
Δ9,12,15
Δ6,9,12,15
Icosanoyl-CoA
Δ11
Δ11,14
Δ8,11,14
Δ5,8,11,14
Δ11,14,17
Δ8,11,14,17
Δ5,8,11,14,17
Docosanoyl-CoA
Δ13
Δ13,16
Δ7,10,13,16
Δ4,7,10,13,16
Δ7,10,13,16,19
Δ4,7,10,13,16,19
Tetracosanoyl-CoA
Δ15
Δ9,12,15,18
Δ6,9,12,15,18
Δ9,12,15,18,21
Δ6,9,12,15,18,21

ARRHYTHMOGENIC RIGHT VENTRICULAR CARDIOMYOPATHY (ARVC)

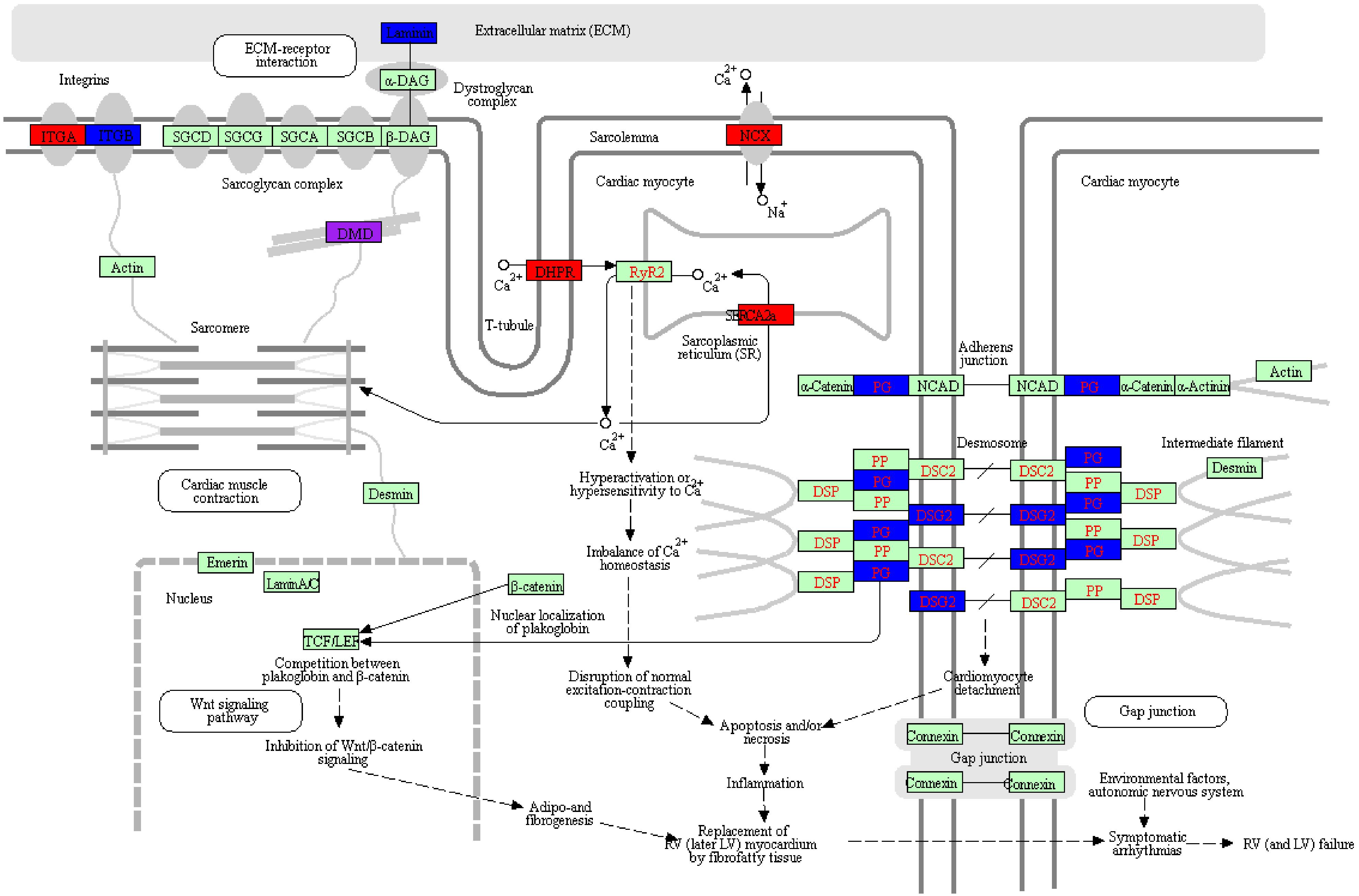

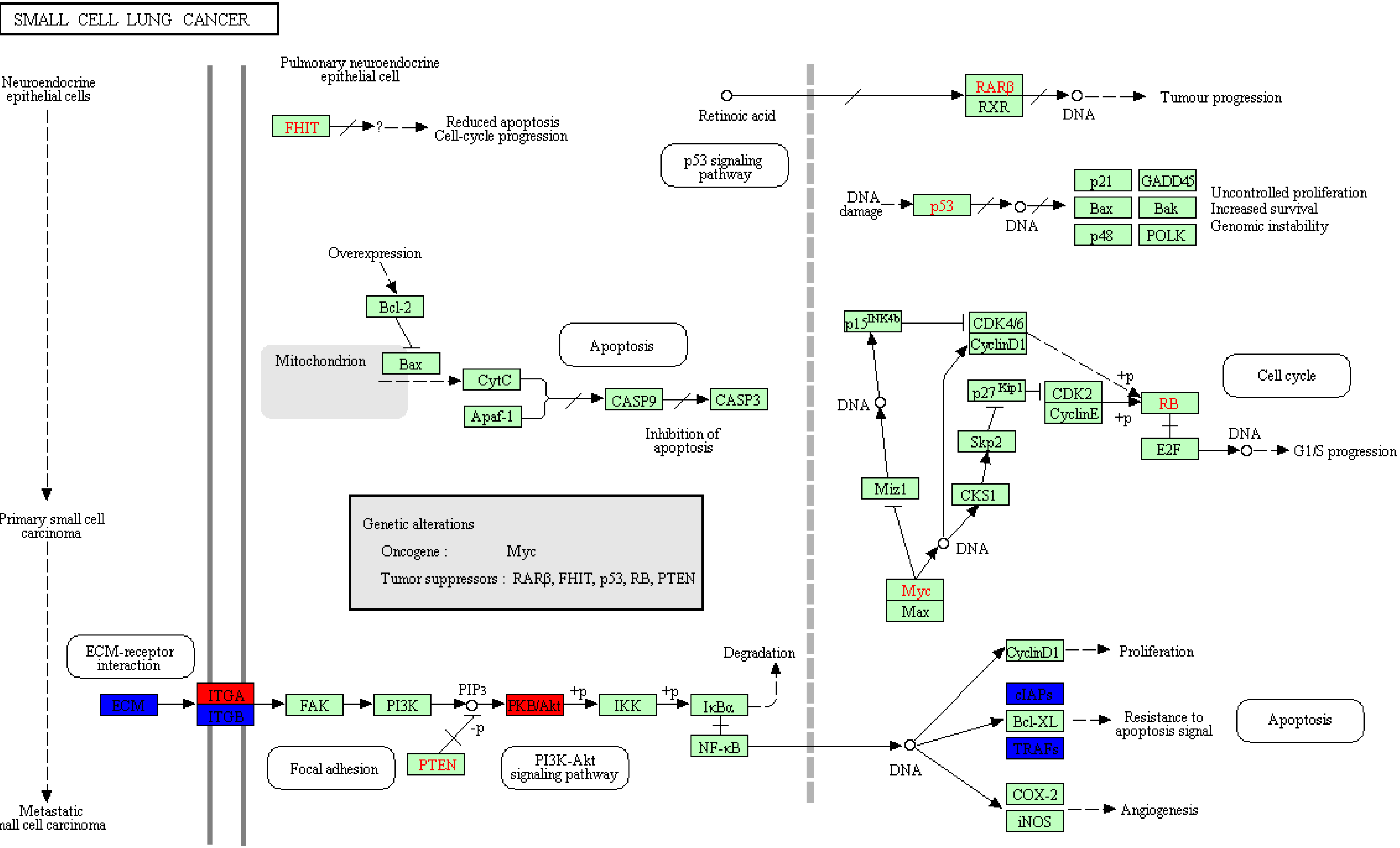

SMALL CELL LUNG CANCER
Neuroendocrine epithelial cells
Primary small cell carcinoma
Metastatic
nall cell carcinoma
Pulmonary neuroendocrine epithelial cell
FHIT
?
Reduced apoptosis
Cell-cycle progression
p53 signaling pathway
Retinoic acid
RARβ
RXR
DNA
Tumour progression
DNA damage
p53
DNA
p21
GADD45
Bax
Bak
p48
POLK
Uncontrolled proliferation
Increased survival
Genomic instability
Overexpression
Bcl-2
Mitochondrion
Bax
CytC
Apaf-1
CASP9
CASP3
Apoptosis
Inhibition of apoptosis
p15INK4b
CDK4/6
CyclinD1
DNA
p27Kip1
CDK2
CyclinE
+p
+p
RB
E2F
Cell cycle
DNA
G1/S progression
Skp2
Miz1
CKS1
DNA
Myc
Max
Genetic alterations
Oncogene : Myc
Tumor suppressors : RARβ, FHIT, p53, RB, PTEN
ECM-receptor interaction
ECM
ITGA
ITGB
FAK
PI3K
PIP3
PKB/Akt
+p
IKK
+p
IκBα
NF-κB
Degradation
-p
PTEN
Focal adhesion
PI3K-Akt signaling pathway
DNA
CyclinD1
Proliferation
cIAPs
Bcl-XL
TRAFs
Resistance to apoptosis signal
Apoptosis
COX-2
iNOS
Angiogenesis

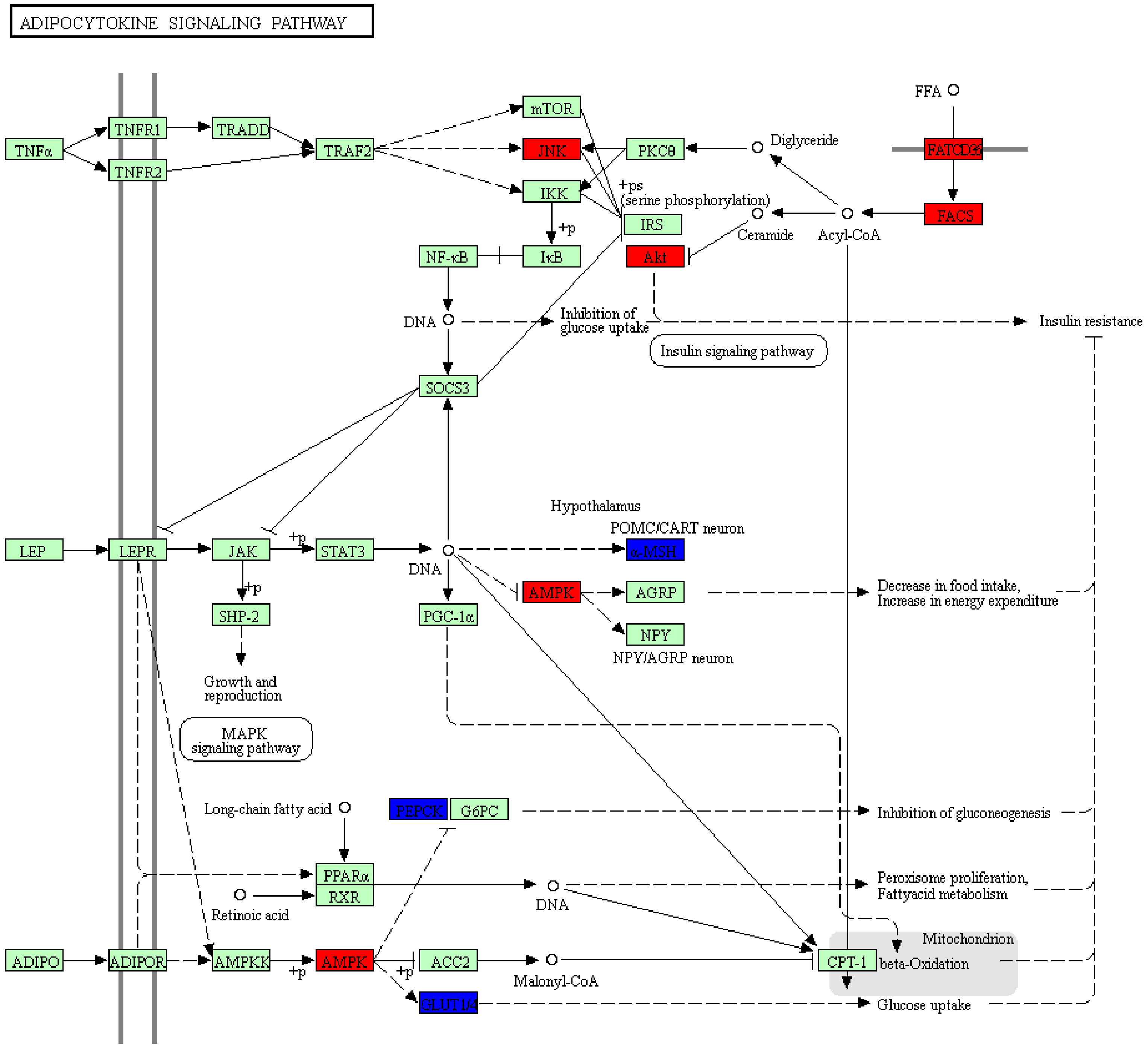
ADIPOCYTOKINE SIGNALING PATHWAY
TNFα
TNFR1
TNFR2
TRADD
TRAF2
mTOR
JNK
IKK
PKCθ
Diglyceride
FFA
FATCD36
FACS
Acyl-CoA
Ceramide
+ps
(serine phosphorylation)
IRS
Akt
+p
NF-κB
IκB
DNA
Inhibition of glucose uptake
Insulin resistance
Insulin signaling pathway
SOCS3
Hypothalamus
POMC/CART neuron
α-MSH
LEP
LEPR
JAK
STAT3
SHP-2
AMPK
AGRP
NPY
NPY/AGRP neuron
PGC-1α
Decrease in food intake,
Increase in energy expenditure
Growth and reproduction
MAPK signaling pathway
Long-chain fatty acid
PEPCK
G6PC
Inhibition of gluconeogenesis
PPARα
RXR
Retinoic acid
Peroxisome proliferation,
Fattyacid metabolism
Mitochondrion
ADIPO
ADIPOR
AMPKK
ACC2
Malonyl-CoA
CPT-1
beta-Oxidation
GLUT1/4
Glucose uptake

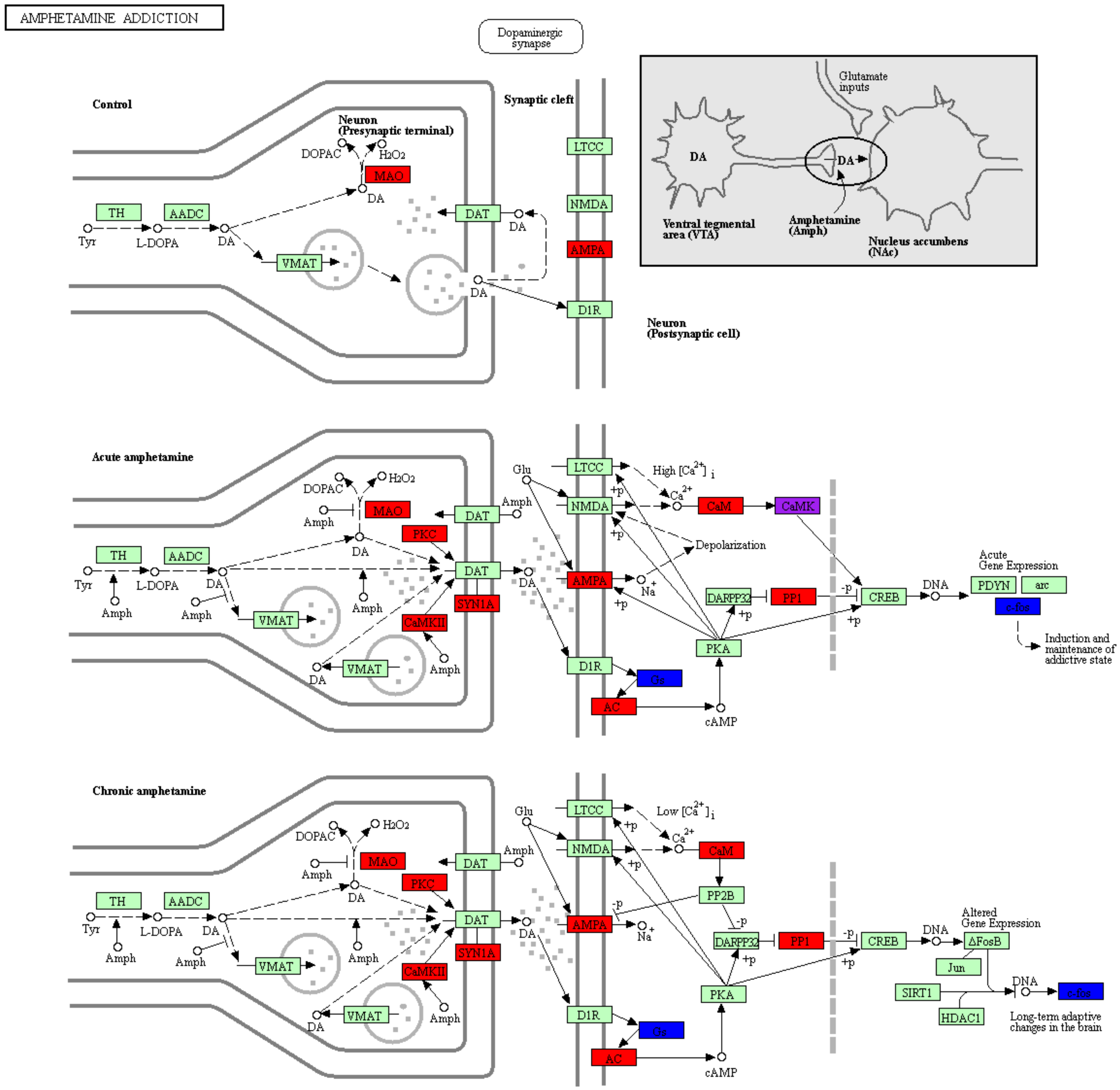

AMPHETAMINE ADDICTION
Dopaminergic synapse
Control
Neuron (Presynaptic terminal)
Synaptic cleft
Neuron (Postsynaptic cell)
Acute amphetamine
Chronic amphetamine
TH
AADC
Tyr
L-DOPA
DA
DOPAC
H2O2
MAO
VMAT
DAT
LTCC
NMDA
AMPA
D1R
Amph
PKC
SYN1A
CaMKII
Glu
High [Ca2+]i
Low [Ca2+]i
Ca2+
CaM
CaMK
Depolarization
Na+
DARPP32
PP1
PP2B
PKA
Gs
AC
cAMP
CREB
DNA
Acute Gene Expression
PDYN
arc
c-fos
Induction and maintenance of addictive state
Altered Gene Expression
ΔFosB
Jun
SIRT1
HDAC1
Long-term adaptive changes in the brain
Glutamate inputs
Ventral tegmental area (VTA)
Amphetamine (Amph)
Nucleus accumbens (NAc)

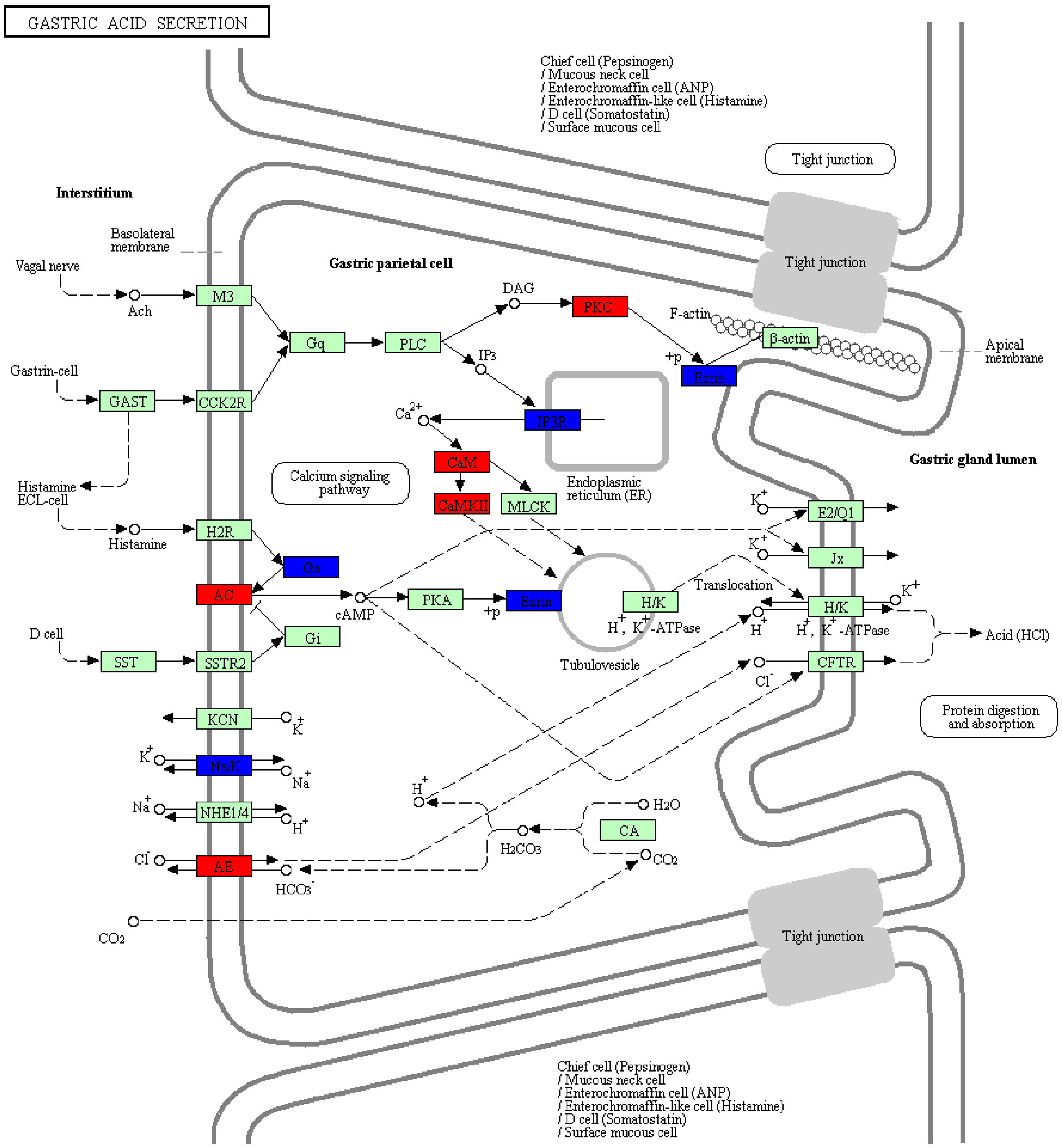
GASTRIC ACID SECRETION
Chief cell (Pepsinogen)
/ Mucous neck cell
/ Enterochromaffin cell (ANP)
/ Enterochromaffin-like cell (Histamine)
/ D cell (Somatostatin)
/ Surface mucous cell
Tight junction
Interstitium
Basolateral membrane
Vagal nerve
Ach
M3
Gastric parietal cell
Gq
PLC
DAG
PKC
F-actin
β-actin
+p
Ezrin
Apical membrane
IP3
Gastrin-cell
GAST
CCK2R
Ca2+
IP3R
CaM
CaMKII
MLCK
Endoplasmic reticulum (ER)
Gastric gland lumen
Calcium signaling pathway
Histamine
ECL-cell
H2R
Gs
AC
cAMP
PKA
Ezrin
H/K
H+, K+-ATPase
Tubulovesicle
Translocation
K+
E2/Q1
Jx
H/K
H+
H+, K+-ATPase
Acid (HCl)
CFTR
Cl-
D cell
SST
SSTR2
Gi
KCN
Na/K
Na+
NHE1/4
AE
HCO3-
H2O
CA
H2CO3
CO2
Protein digestion and absorption

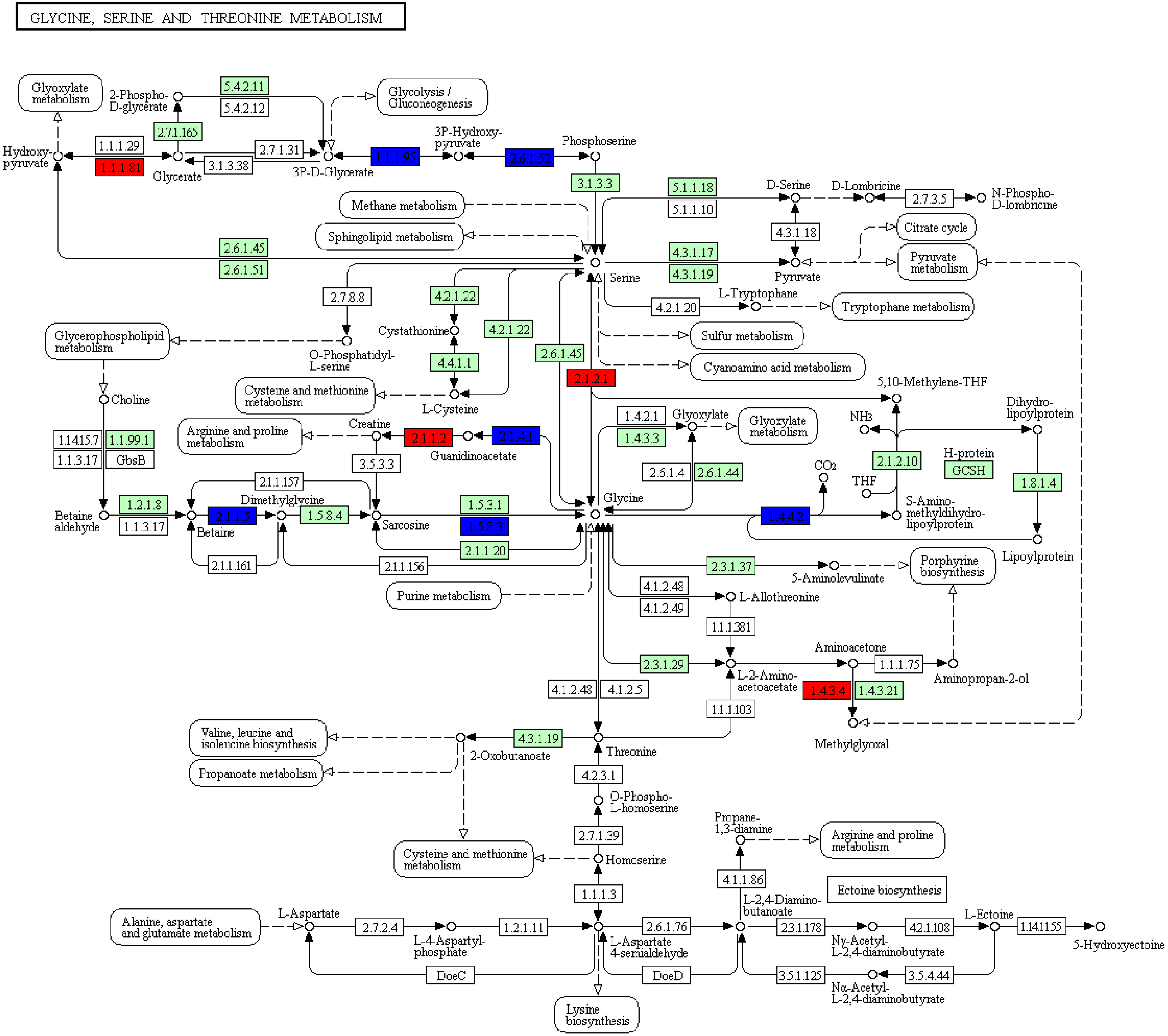
GLYCINE, SERINE AND THREONINE METABOLISM
Glyoxylate metabolism
2-Phospho-D-glycerate
5.4.2.11
5.4.2.12
Glycolysis / Gluconeogenesis
2.7.1.165
3P-Hydroxy-pyruvate
Phosphoserine
Hydroxy-pyruvate
1.1.1.29
1.1.1.81
2.7.1.31
3.1.3.38
1.1.1.95
2.6.1.52
Glycerate
3P-D-Glycerate
3.1.3.3
5.1.1.18
5.1.1.10
D-Serine
D-Lombricine
2.7.3.5
N-Phospho-D-lombricine
Methane metabolism
Sphingolipid metabolism
4.3.1.18
Citrate cycle
2.6.1.45
2.6.1.51
4.3.1.17
4.3.1.19
Pyruvate metabolism
Serine
Pyruvate
2.7.8.8
4.2.1.22
4.2.1.20
L-Tryptophane
Tryptophane metabolism
Glycerophospholipid metabolism
Cystathionine
4.2.1.22
Sulfur metabolism
O-Phosphatidyl-L-serine
2.6.1.45
4.4.1.1
Cyanoamino acid metabolism
Choline
Cysteine and methionine metabolism
L-Cysteine
2.1.2.1
5,10-Methylene-THF
1.4.2.1
Glyoxylate
Glyoxylate metabolism
Dihydro-lipoylprotein
NH3
Arginine and proline metabolism
Creatine
2.1.1.2
2.1.4.1
Guanidinoacetate
1.4.3.3
1.1.415.7
1.1.99.1
1.1.3.17
GbsB
3.5.3.3
2.6.1.4
2.6.1.44
CO2
2.1.2.10
H-protein
GCSH
THF
2.1.1.157
1.8.1.4
Dimethylglycine
Glycine
S-Amino-methyldihydro-lipoylprotein
Betaine aldehyde
1.2.1.8
1.1.3.17
2.1.1.5
1.5.8.4
Sarcosine
1.5.3.1
1.5.8.3
1.4.4.2
Betaine
Lipoylprotein
2.1.1.20
2.1.1.161
2.1.1.156
2.3.1.37
5-Aminolevulinate
Porphyrine biosynthesis
4.1.2.48
4.1.2.49
L-Allothreonine
Purine metabolism
1.1.1.381
Aminoacetone
2.3.1.29
1.1.1.75
L-2-Amino-acetoacetate
Aminopropan-2-ol
4.1.2.48
4.1.2.5
1.4.3.4
1.4.3.21
1.1.1.103
Valine, leucine and isoleucine biosynthesis
4.3.1.19
Methylglyoxal
Threonine
2-Oxobutanoate
Propanoate metabolism
4.2.3.1
O-Phospho-L-homoserine
2.7.1.39
Propane-1,3-diamine
Arginine and proline metabolism
Cysteine and methionine metabolism
Homoserine
4.1.1.86
Ectoine biosynthesis
1.1.1.3
L-2,4-Diamino-butanoate
L-Aspartate
L-Ectoine
Alanine, aspartate and glutamate metabolism
2.7.2.4
1.2.1.11
2.6.1.76
2.3.1.178
4.2.1.108
1.14.11.55
L-4-Aspartyl-phosphate
L-Aspartate 4-semialdehyde
Nγ-Acetyl-L-2,4-diaminobutyrate
5-Hydroxyectoine
DoeC
DoeD
3.5.1.125
3.5.4.44
Nα-Acetyl-L-2,4-diaminobutyrate
Lysine biosynthesis

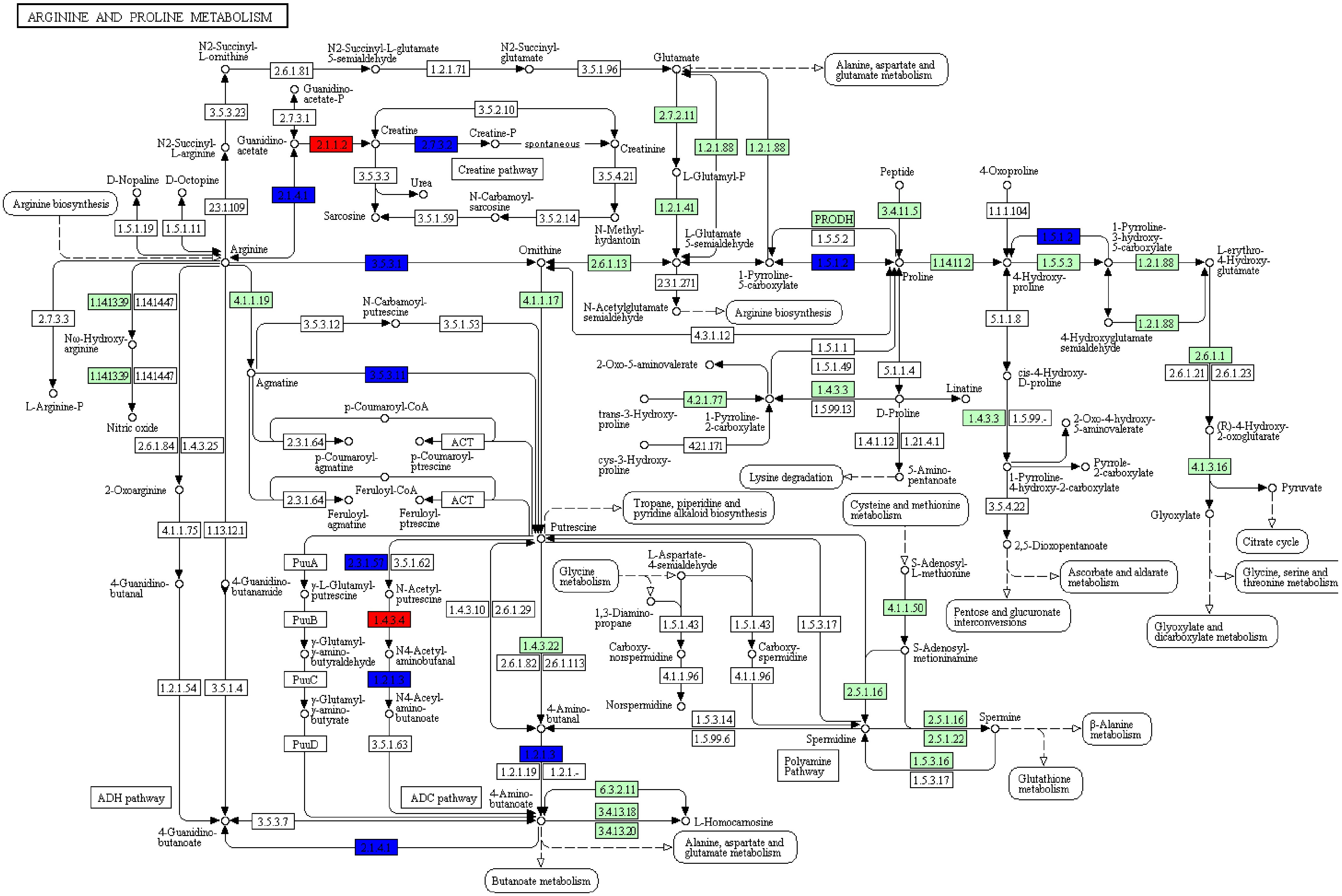
ARGININE AND PROLINE METABOLISM
N2-Succinyl-L-ornithine
2.6.1.81
N2-Succinyl-L-glutamate 5-semialdehyde
1.2.1.71
N2-Succinyl-glutamate
3.5.1.96
Glutamate
Alanine, aspartate and glutamate metabolism
Guanidino-acetate-P
3.5.3.23
2.7.3.1
3.5.2.10
N2-Succinyl-L-arginine
Guanidino-acetate
2.1.1.2
Creatine
2.7.3.2
Creatine-P
spontaneous
Creatinine
2.7.2.11
1.2.1.88
1.2.1.88
Creatine pathway
3.5.3.3
Urea
3.5.4.21
L-Glutamyl-P
D-Nopaline
D-Octopine
N-Carbamoyl-sarcosine
Peptide
4-Oxoproline
Arginine biosynthesis
23.1.109
2.1.4.1
Sarcosine
3.5.1.59
3.5.2.14
1.2.1.41
3.4.11.5
1.1.1.104
1.5.1.19
1.5.1.11
N-Methyl-hydantoin
PRODH
L-Glutamate 5-semialdehyde
1.5.5.2
1.5.1.2
1-Pyrroline-3-hydroxy-5-carboxylate
Arginine
3.5.3.1
Ornithine
2.6.1.13
1.5.1.2
1.14.11.2
1.5.5.3
1.2.1.88
L-erythro-4-Hydroxy-glutamate
Proline
23.1.271
1-Pyrroline-5-carboxylate
4-Hydroxy-proline
1.14.13.39
1.14.14.47
4.1.1.19
4.1.1.17
N-Acetylglutamate semialdehyde
Arginine biosynthesis
2.7.3.3
N-Carbamoyl-putrescine
3.5.3.12
3.5.1.53
4.3.1.12
5.1.1.8
1.2.1.88
Nω-Hydroxy-arginine
4-Hydroxyglutamate semialdehyde
1.5.1.1
2.6.1.1
1.5.1.49
2-Oxo-5-aminovalerate
1.14.13.39
1.14.14.47
3.5.3.11
5.1.1.4
2.6.1.21
2.6.1.23
Agmatine
1.4.3.3
cis-4-Hydroxy-D-proline
4.2.1.77
Linatine
L-Arginine-P
1.5.99.13
trans-3-Hydroxy-proline
1-Pyrroline-2-carboxylate
D-Proline
p-Coumaroyl-CoA
1.4.3.3
1.5.99.-
2-Oxo-4-hydroxy-5-aminovalerate
(R)-4-Hydroxy-2-oxoglutarate
Nitric oxide
2.3.1.64
ACT
4.2.1.171
1.4.1.12
1.21.4.1
2.6.1.84
1.4.3.25
p-Coumaroyl-agmatine
p-Coumaroyl-ptrescine
cys-3-Hydroxy-proline
Pyrrole-2-carboxylate
Lysine degradation
5-Amino-pentanoate
4.1.3.16
1-Pyrroline-4-hydroxy-2-carboxylate
2-Oxoarginine
Feruloyl-CoA
Pyruvate
2.3.1.64
ACT
Tropane, piperidine and pyridine alkaloid biosynthesis
Cysteine and methionine metabolism
3.5.4.22
Feruloyl-agmatine
Feruloyl-ptrescine
Glyoxylate
Putrescine
4.1.1.75
1.13.12.1
2,5-Dioxopentanoate
Citrate cycle
PuuA
2.3.1.57
3.5.1.62
L-Aspartate-4-semialdehyde
S-Adenosyl-L-methionine
Glycine metabolism
4-Guanidino-butanal
4-Guanidino-butanamide
γ-L-Glutamyl-putrescine
N-Acetyl-putrescine
Ascorbate and aldarate metabolism
Glycine, serine and threonine metabolism
1.4.3.10
2.6.1.29
1,3-Diamino-propane
4.1.1.50
Pentose and glucuronate interconversions
PuuB
1.4.3.4
1.5.1.43
1.5.1.43
1.5.3.17
Glyoxylate and dicarboxylate metabolism
γ-Glutamyl-γ-amino-butyraldehyde
N4-Acetyl-aminobutanal
1.4.3.22
Carboxy-norspermidine
Carboxy-spermidine
S-Adenosyl-metioninamine
2.6.1.82
2.6.1.113
PuuC
1.2.1.3
4.1.1.96
4.1.1.96
1.2.1.54
3.5.1.4
2.5.1.16
γ-Glutamyl-γ-amino-butyrate
N4-Acetyl-amino-butanoate
Norspermidine
2.5.1.16
Spermine
4-Amino-butanal
1.5.3.14
β-Alanine metabolism
2.5.1.22
Spermidine
PuuD
3.5.1.63
1.5.99.6
1.2.1.3
Polyamine Pathway
1.5.3.16
1.2.1.19
1.2.1.-
1.5.3.17
Glutathione metabolism
ADH pathway
ADC pathway
4-Amino-butanoate
6.3.2.11
3.4.13.18
L-Homocarnosine
3.5.3.7
4-Guanidino-butanoate
3.4.13.20
2.1.4.1
Alanine, aspartate and glutamate metabolism
Butanoate metabolism

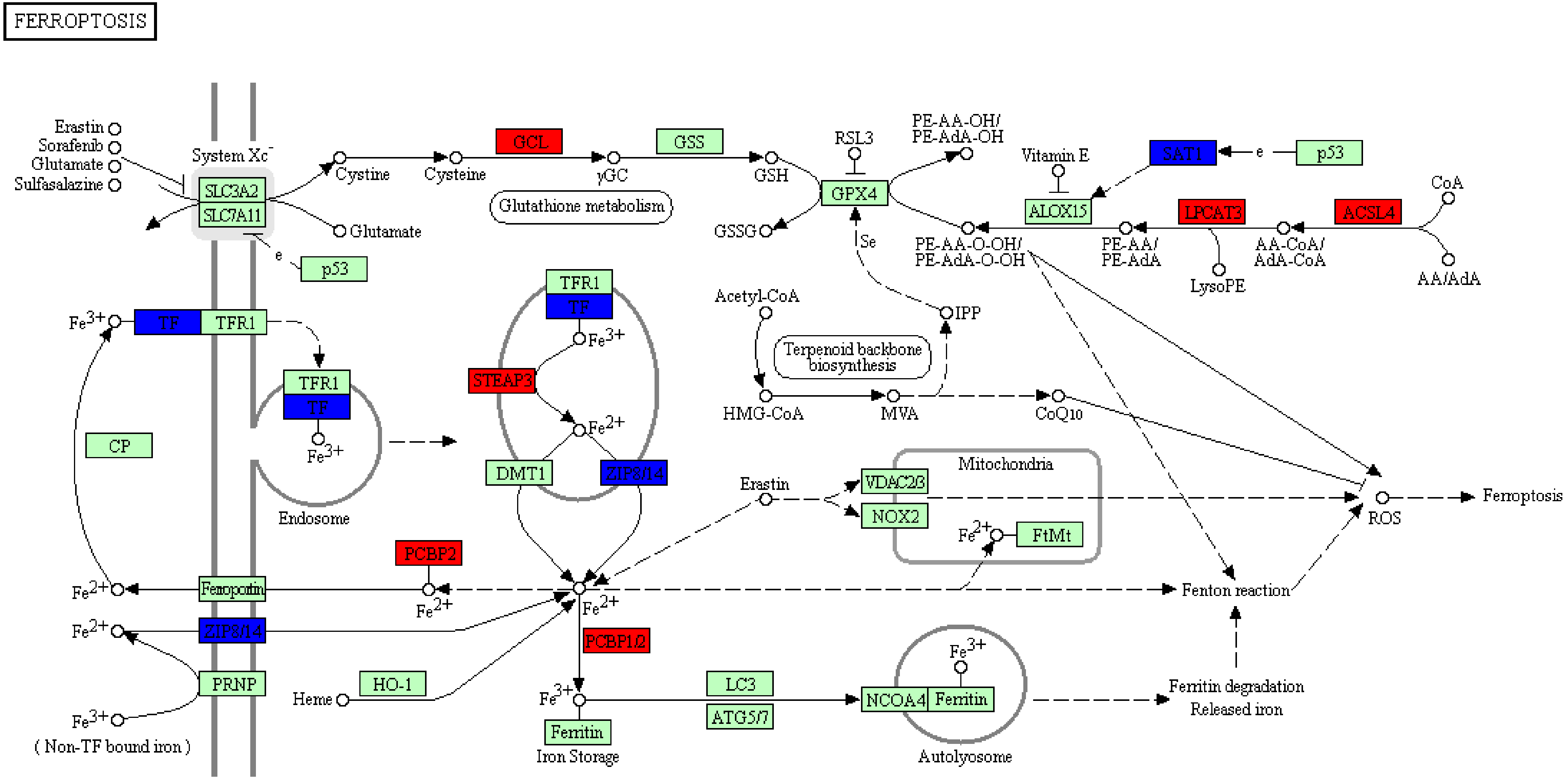
FERROPTOSIS
Erastin
Sorafenib
Glutamate
Sulfasalazine
System Xc-
SLC3A2
SLC7A11
p53
Cystine
Cysteine
Glutamate
GCL
γGC
GSS
GSH
Glutathione metabolism
GSSG
RSL3
GPX4
Se
PE-AA-OH/
PE-AdA-OH
Vitamin E
ALOX15
SAT1
p53
PE-AA-O-OH/
PE-AdA-O-OH
PE-AA/
PE-AdA
LPCAT3
LysoPE
AA-CoA/
AdA-CoA
ACSL4
CoA
AA/AdA
Acetyl-CoA
Terpenoid backbone biosynthesis
HMG-CoA
MVA
IPP
CoQ10
Fe3+
TF
TFR1
Endosome
CP
STEAP3
Fe2+
DMT1
ZIP8/14
Erastin
VDAC2/3
NOX2
Mitochondria
FtMt
ROS
Ferroptosis
PCBP2
Ferroportin
ZIP8/14
PRNP
(Non-TF bound iron)
Heme
HO-1
PCBP1/2
Ferritin
Iron Storage
LC3
ATG5/7
NCOA4
Autolysosome
Fenton reaction
Ferritin degradation
Released iron

CYSTEINE AND METHIONINE METABOLISM

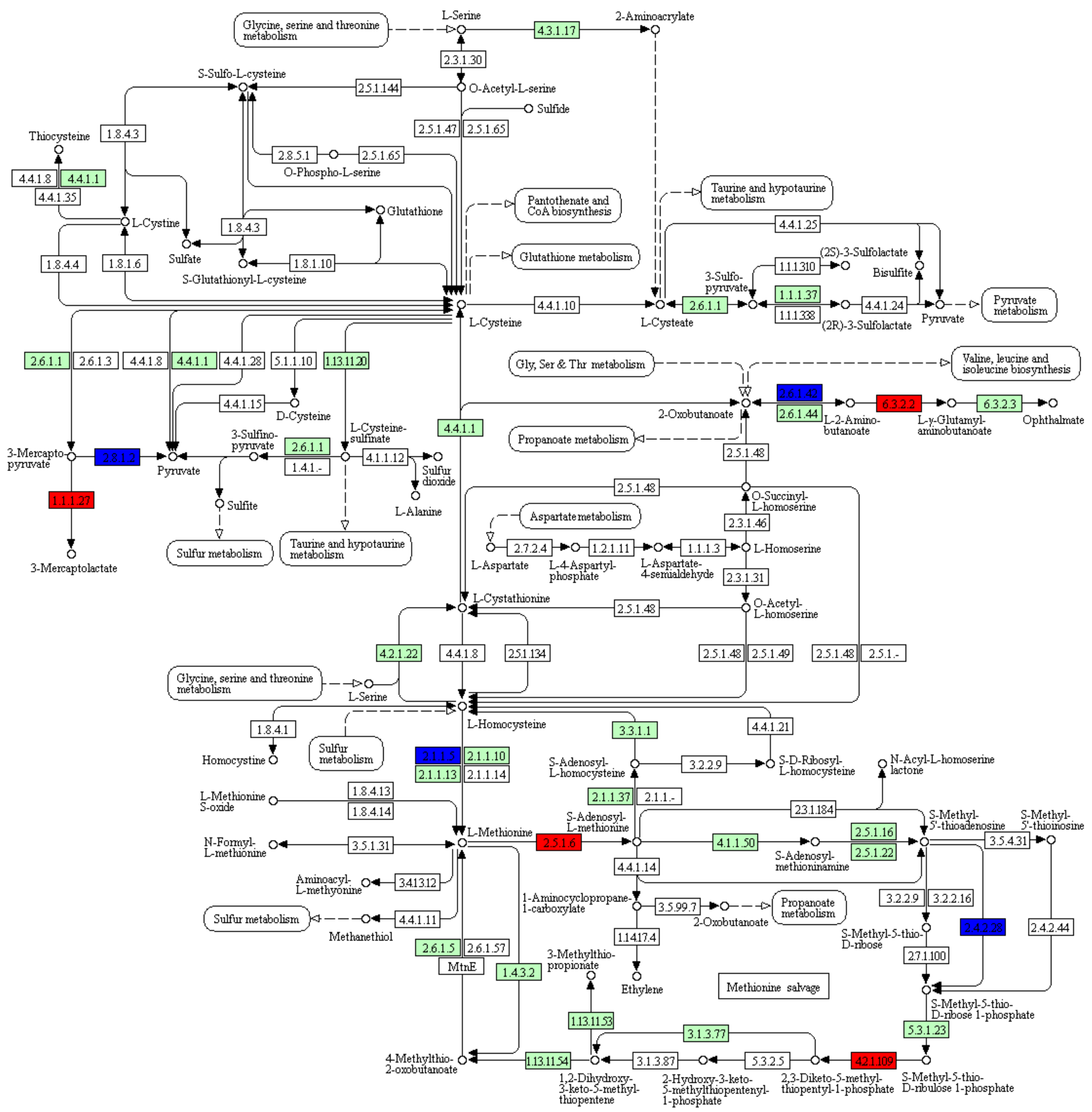

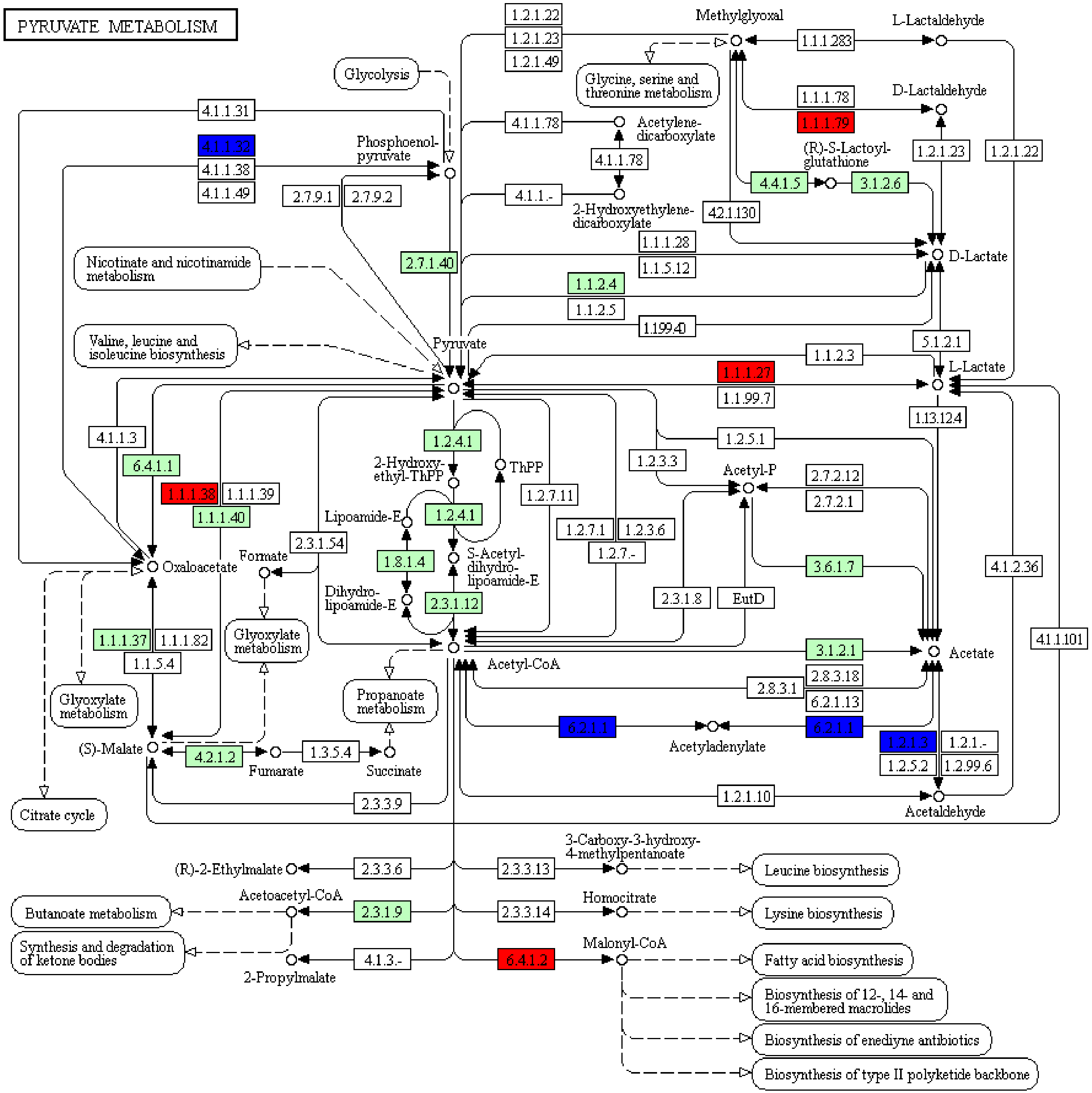
PYRUVATE METABOLISM
Glycolysis
Phosphoenol-pyruvate
Pyruvate
Methylglyoxal
L-Lactaldehyde
D-Lactaldehyde
Glycine, serine and threonine metabolism
Acetylene-dicarboxylate
2-Hydroxyethylene-dicarboxylate
(R)-S-Lactoyl-glutathione
D-Lactate
L-Lactate
Nicotinate and nicotinamide metabolism
Valine, leucine and isoleucine biosynthesis
2-Hydroxy-ethyl-ThPP
ThPP
Lipoamide-E
S-Acetyl-dihydro-lipoamide-E
Dihydro-lipoamide-E
Acetyl-P
Oxaloacetate
Formate
Glyoxylate metabolism
Acetyl-CoA
Acetate
Propanoate metabolism
Acetyladenylate
Acetaldehyde
(S)-Malate
Fumarate
Succinate
Citrate cycle
3-Carboxy-3-hydroxy-4-methylpentanoate
Leucine biosynthesis
(R)-2-Ethylmalate
Acetoacetyl-CoA
Homocitrate
Lysine biosynthesis
Butanoate metabolism
Synthesis and degradation of ketone bodies
2-Propylmalate
Malonyl-CoA
Fatty acid biosynthesis
Biosynthesis of 12-, 14- and 16-membered macrolides
Biosynthesis of enediyne antibiotics
Biosynthesis of type II polyketide backbone
1.2.1.22
1.2.1.23
1.2.1.49
4.1.1.31
4.1.1.32
4.1.1.38
4.1.1.49
2.7.9.1
2.7.9.2
4.1.1.78
4.1.1.-
1.1.1.283
1.1.1.78
1.1.1.79
4.4.1.5
3.1.2.6
4.2.1.130
1.1.1.28
1.1.5.12
2.7.1.40
1.1.2.4
1.1.2.5
1.1.99.40
5.1.2.1
1.1.2.3
1.1.1.27
1.1.99.7
1.13.12.4
1.2.5.1
1.2.3.3
2.7.2.12
2.7.2.1
3.6.1.7
4.1.2.36
4.1.1.101
1.2.4.1
1.2.7.11
1.2.7.1
1.2.3.6
1.2.7.-
1.8.1.4
2.3.1.12
2.3.1.54
2.3.1.8
EutD
4.1.1.3
6.4.1.1
1.1.1.38
1.1.1.39
1.1.1.40
1.1.1.37
1.1.1.82
1.1.5.4
3.1.2.1
2.8.3.18
2.8.3.1
6.2.1.13
6.2.1.1
1.2.1.3
1.2.1.-
1.2.5.2
1.2.99.6
1.2.1.10
4.2.1.2
1.3.5.4
2.3.3.9
2.3.3.6
2.3.3.13
2.3.1.9
2.3.3.14
4.1.3.-
6.4.1.2

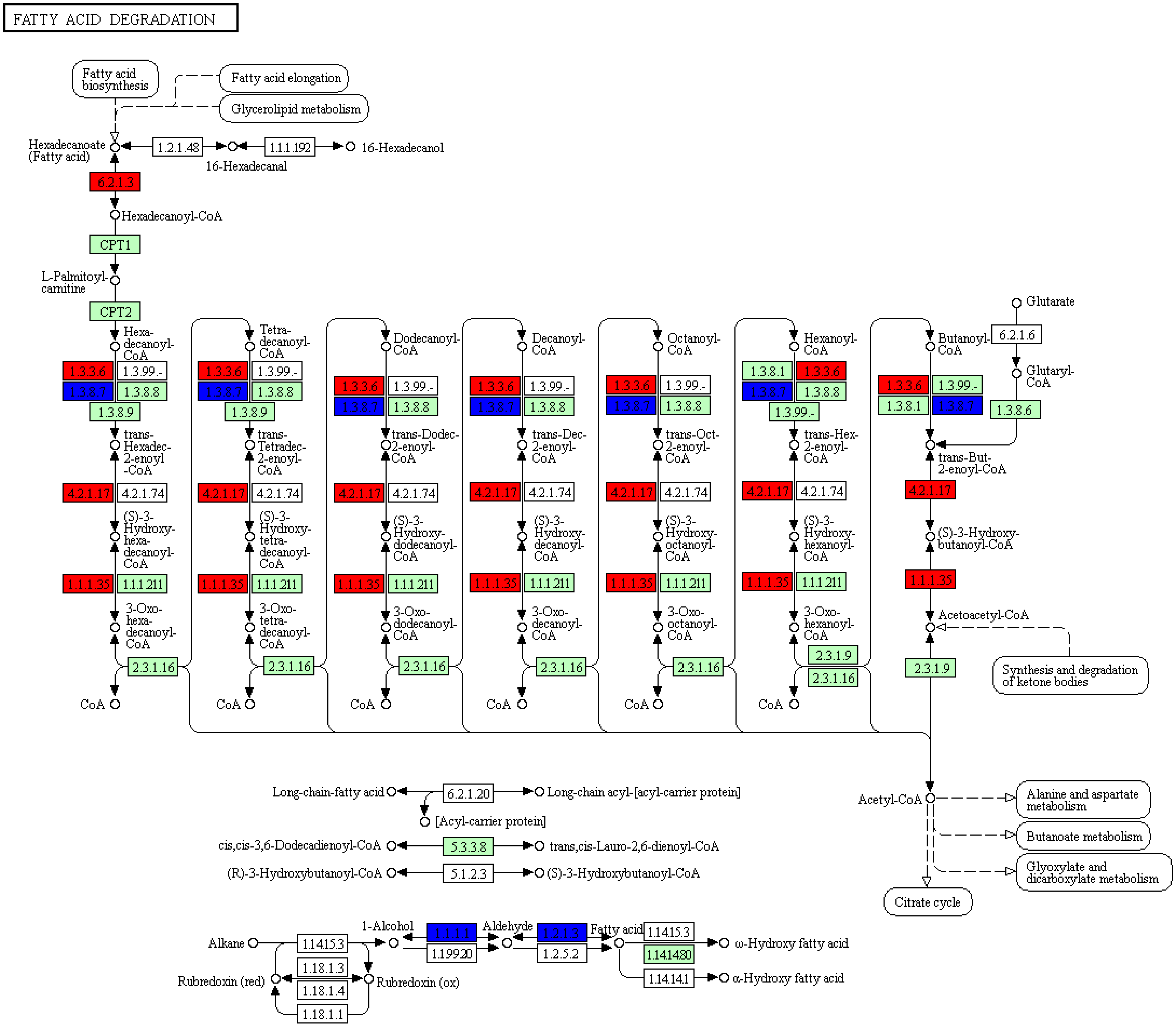
FATTY ACID DEGRADATION
Fatty acid biosynthesis
Fatty acid elongation
Glycerolipid metabolism
Hexadecanoate (Fatty acid)
1.2.1.48
16-Hexadecanal
1.1.1.192
16-Hexadecanol
6.2.1.3
Hexadecanoyl-CoA
CPT1
L-Palmitoyl-carnitine
CPT2
Hexa-decanoyl-CoA
Tetra-decanoyl-CoA
Dodecanoyl-CoA
Decanoyl-CoA
Octanoyl-CoA
Hexanoyl-CoA
Butanoyl-CoA
Glutarate
6.2.1.6
Glutaryl-CoA
1.3.8.6
1.3.3.6
1.3.99.-
1.3.8.7
1.3.8.8
1.3.8.9
1.3.8.1
trans-Hexadec-2-enoyl-CoA
trans-Tetradec-2-enoyl-CoA
trans-Dodec-2-enoyl-CoA
trans-Dec-2-enoyl-CoA
trans-Oct-2-enoyl-CoA
trans-Hex-2-enoyl-CoA
trans-But-2-enoyl-CoA
4.2.1.17
4.2.1.74
(S)-3-Hydroxy-hexadecanoyl-CoA
(S)-3-Hydroxy-tetradecanoyl-CoA
(S)-3-Hydroxy-dodecanoyl-CoA
(S)-3-Hydroxy-decanoyl-CoA
(S)-3-Hydroxy-octanoyl-CoA
(S)-3-Hydroxy-hexanoyl-CoA
(S)-3-Hydroxy-butanoyl-CoA
1.1.1.35
1.1.1.211
3-Oxo-hexadecanoyl-CoA
3-Oxo-tetradecanoyl-CoA
3-Oxo-dodecanoyl-CoA
3-Oxo-decanoyl-CoA
3-Oxo-octanoyl-CoA
3-Oxo-hexanoyl-CoA
Acetoacetyl-CoA
2.3.1.16
2.3.1.9
CoA
Synthesis and degradation of ketone bodies
Acetyl-CoA
Alanine and aspartate metabolism
Butanoate metabolism
Glyoxylate and dicarboxylate metabolism
Citrate cycle
Long-chain-fatty acid
6.2.1.20
Long-chain acyl-[acyl-carrier protein]
[Acyl-carrier protein]
cis,cis-3,6-Dodecadienoyl-CoA
5.3.3.8
trans,cis-Lauro-2,6-dienoyl-CoA
(R)-3-Hydroxybutanoyl-CoA
5.1.2.3
(S)-3-Hydroxybutanoyl-CoA
Alkane
1.14.15.3
1-Alcohol
1.1.1.1
1.1.99.20
Aldehyde
1.2.1.3
1.2.5.2
Fatty acid
1.14.14.80
ω-Hydroxy fatty acid
1.14.14.1
α-Hydroxy fatty acid
1.18.1.3
1.18.1.4
1.18.1.1
Rubredoxin (red)
Rubredoxin (ox)

BIOSYNTHESIS OF UNSATURATED FATTY ACIDS

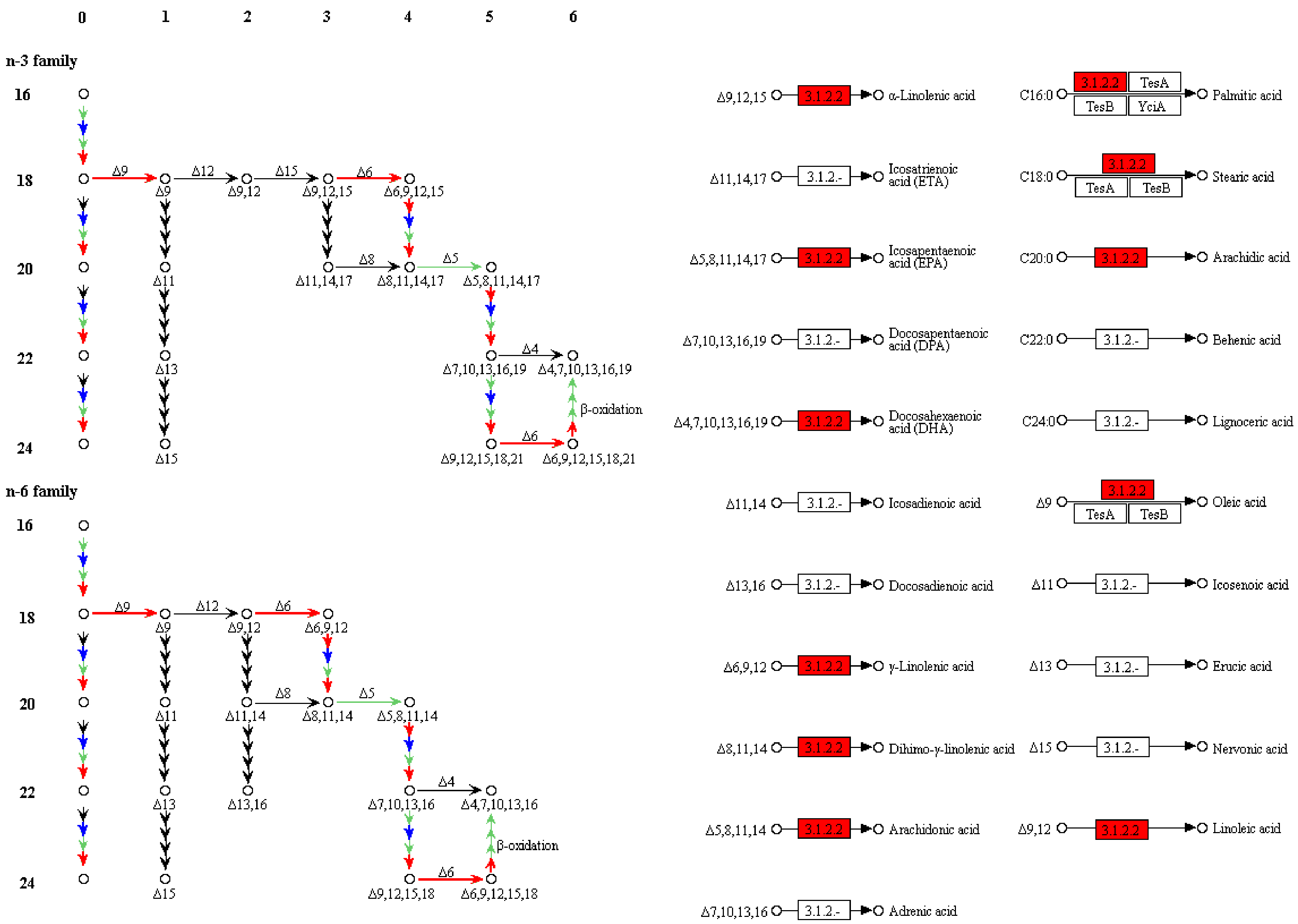

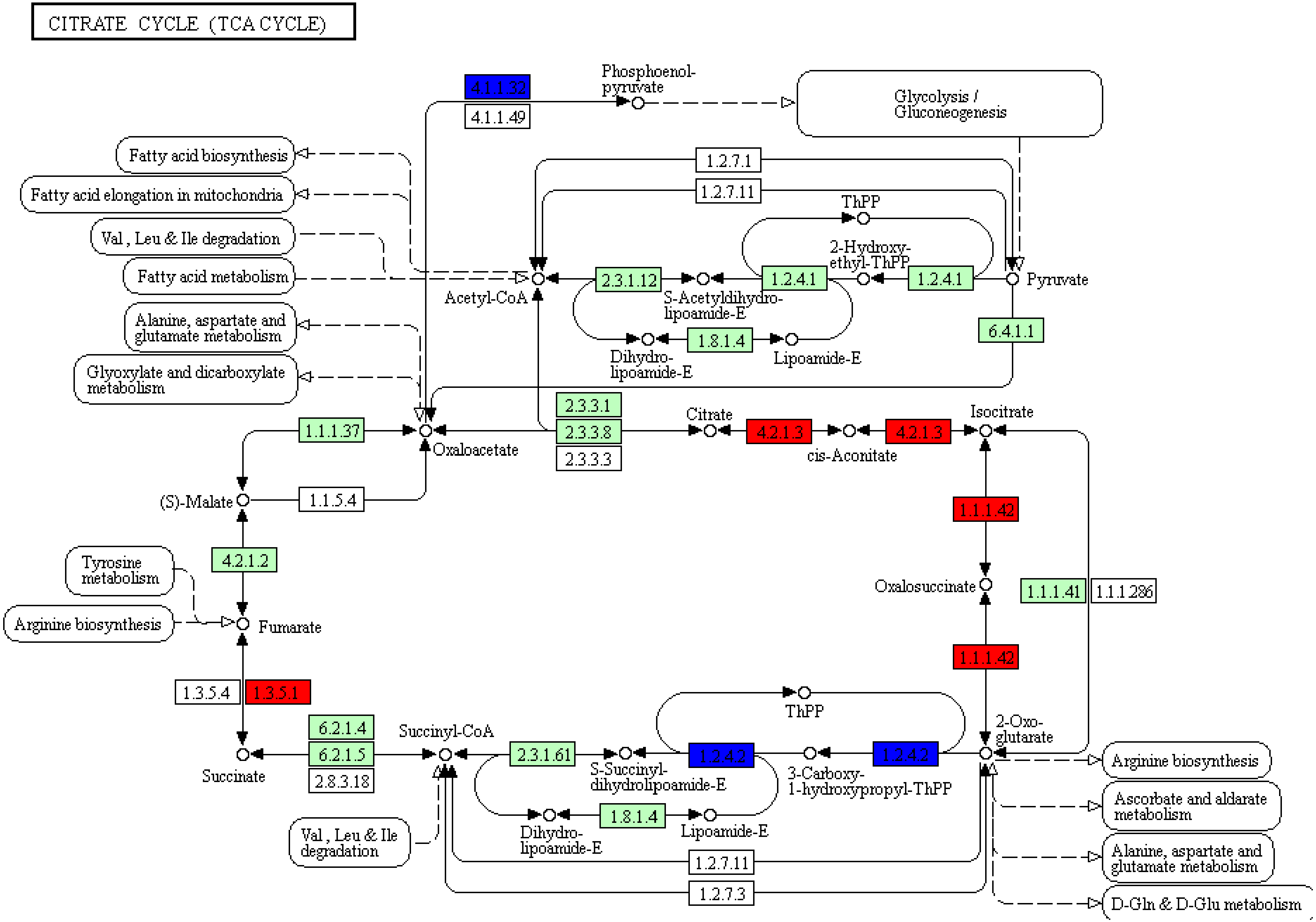
CITRATE CYCLE (TCA CYCLE)
4.1.1.32
4.1.1.49
Phosphoenol-pyruvate
Glycolysis / Gluconeogenesis
Fatty acid biosynthesis
Fatty acid elongation in mitochondria
Val, Leu & Ile degradation
Fatty acid metabolism
Alanine, aspartate and glutamate metabolism
Glyoxylate and dicarboxylate metabolism
1.2.7.1
1.2.7.11
ThPP
2-Hydroxy-ethyl-ThPP
2.3.1.12
1.2.4.1
1.2.4.1
Pyruvate
Acetyl-CoA
S-Acetyldihydro-lipoamide-E
1.8.1.4
Dihydro-lipoamide-E
Lipoamide-E
6.4.1.1
2.3.3.1
2.3.3.8
2.3.3.3
Citrate
4.2.1.3
4.2.1.3
cis-Aconitate
Isocitrate
1.1.1.37
Oxaloacetate
(S)-Malate
1.1.5.4
1.1.1.42
Oxalosuccinate
1.1.1.41
1.1.1.286
4.2.1.2
Tyrosine metabolism
Arginine biosynthesis
Fumarate
1.1.1.42
1.3.5.4
1.3.5.1
6.2.1.4
6.2.1.5
2.8.3.18
Succinate
Succinyl-CoA
2.3.1.61
ThPP
S-Succinyl-dihydrolipoamide-E
1.2.4.2
1.2.4.2
3-Carboxy-1-hydroxypropyl-ThPP
2-Oxo-glutarate
Arginine biosynthesis
Ascorbate and aldarate metabolism
Alanine, aspartate and glutamate metabolism
D-Gln & D-Glu metabolism
Val, Leu & Ile degradation
Dihydro-lipoamide-E
1.8.1.4
Lipoamide-E
1.2.7.11
1.2.7.3

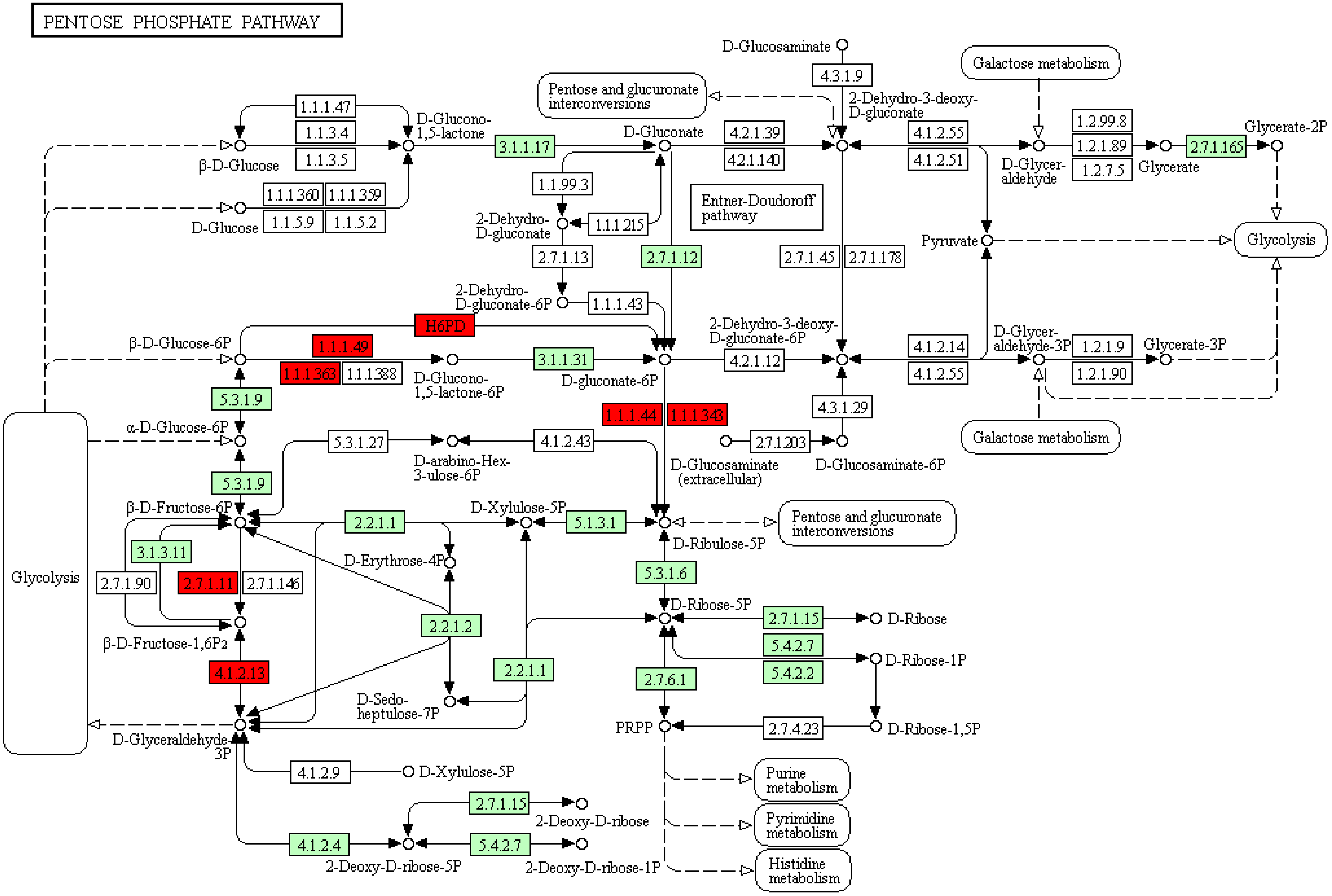
PENTOSE PHOSPHATE PATHWAY
D-Glucosaminate
4.3.1.9
Galactose metabolism
Pentose and glucuronate interconversions
2-Dehydro-3-deoxy-D-gluconate
1.1.1.47
1.1.3.4
1.1.3.5
D-Glucono-1,5-lactone
3.1.1.17
D-Gluconate
4.2.1.39
4.2.1.140
4.1.2.55
4.1.2.51
1.2.99.8
1.2.1.89
1.2.7.5
Glyceraldehyde
Glycerate
2.7.1.165
Glycerate-2P
β-D-Glucose
1.1.1.360
1.1.1.359
1.1.5.9
1.1.5.2
D-Glucose
1.1.99.3
2-Dehydro-D-gluconate
1.1.1.215
Entner-Doudoroff pathway
Pyruvate
Glycolysis
2.7.1.13
2.7.1.12
2.7.1.45
2.7.1.178
2-Dehydro-D-gluconate-6P
1.1.1.43
H6PD
β-D-Glucose-6P
1.1.1.49
1.1.1.363
1.1.1.388
D-Glucono-1,5-lactone-6P
3.1.1.31
D-gluconate-6P
2-Dehydro-3-deoxy-D-gluconate-6P
4.2.1.12
4.1.2.14
4.1.2.55
D-Glyceraldehyde-3P
1.2.1.9
1.2.1.90
Glycerate-3P
5.3.1.9
α-D-Glucose-6P
1.1.1.44
1.1.1.343
4.3.1.29
2.7.1.203
D-Glucosaminate (extracellular)
D-Glucosaminate-6P
5.3.1.27
D-arabino-Hex-3-ulose-6P
4.1.2.43
β-D-Fructose-6P
2.2.1.1
D-Xylulose-5P
5.1.3.1
D-Ribulose-5P
3.1.3.11
D-Erythrose-4P
2.7.1.90
2.7.1.11
2.7.1.146
5.3.1.6
D-Ribose-5P
2.7.1.15
D-Ribose
5.4.2.7
5.4.2.2
D-Ribose-1P
2.2.1.2
β-D-Fructose-1,6P2
4.1.2.13
D-Sedo-heptulose-7P
2.7.6.1
PRPP
2.7.4.23
D-Ribose-1,5P
D-Glyceraldehyde-3P
4.1.2.9
D-Xylulose-5P
Purine metabolism
Pyrimidine metabolism
2-Deoxy-D-ribose
4.1.2.4
2-Deoxy-D-ribose-5P
2-Deoxy-D-ribose-1P
Histidine metabolism

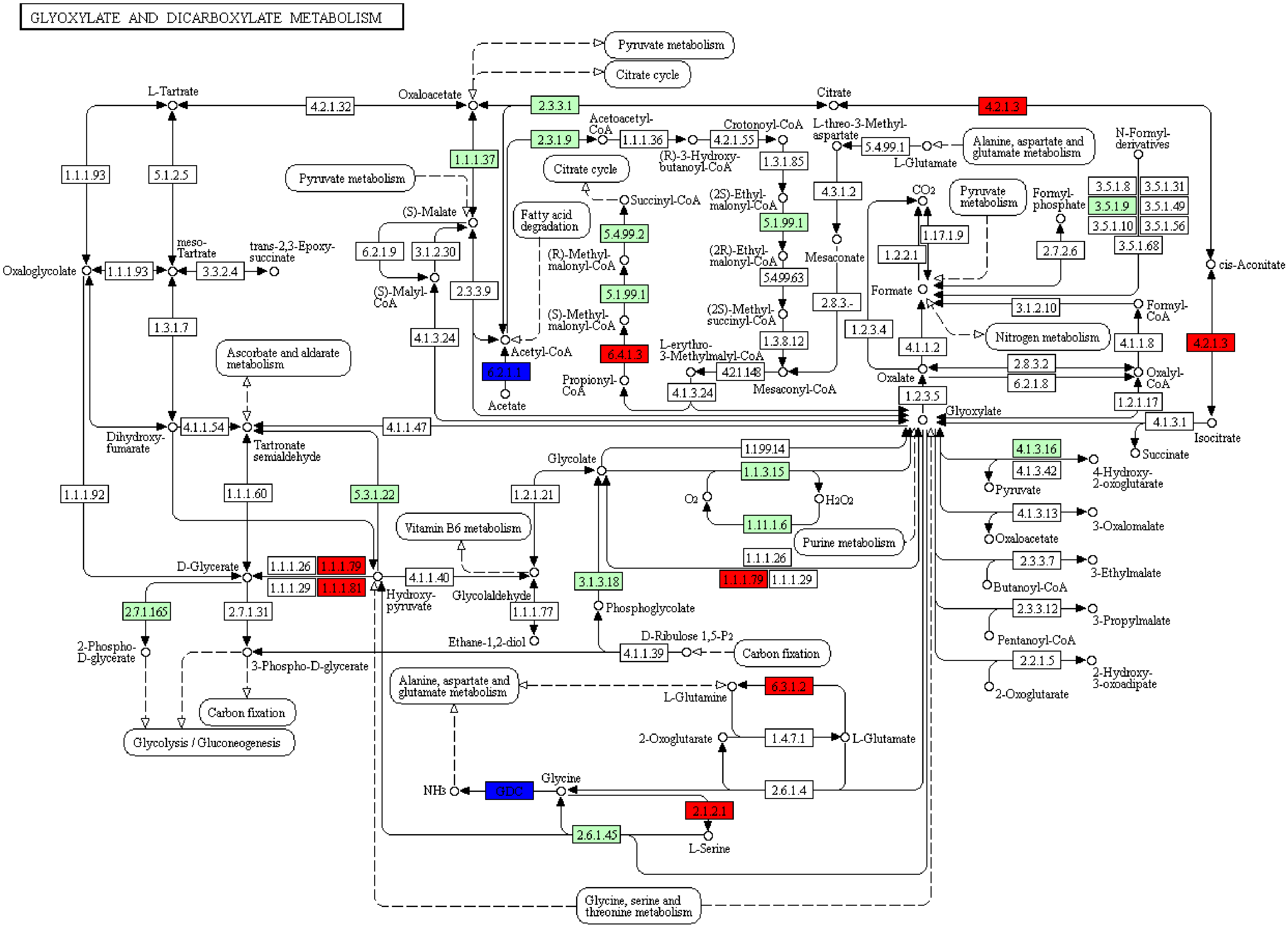
GLYOXYLATE AND DICARBOXYLATE METABOLISM
Pyruvate metabolism
Citrate cycle
L-Tartrate
4.2.1.32
Oxaloacetate
2.3.3.1
Citrate
4.2.1.3
Acetoacetyl-CoA
2.3.1.9
1.1.1.36
Crotonoyl-CoA
4.2.1.55
L-threo-3-Methyl-aspartate
5.4.99.1
L-Glutamate
Alanine, aspartate and glutamate metabolism
N-Formyl-derivatives
1.1.1.93
5.1.2.5
1.1.1.37
Pyruvate metabolism
Citrate cycle
(R)-3-Hydroxy-butanoyl-CoA
1.3.1.85
4.3.1.2
CO2
Pyruvate metabolism
Formyl-phosphate
3.5.1.8
3.5.1.31
3.5.1.9
3.5.1.49
3.5.1.10
3.5.1.56
3.5.1.68
(S)-Malate
Fatty acid degradation
Succinyl-CoA
(2S)-Ethyl-malonyl-CoA
5.4.99.2
5.1.99.1
1.17.1.9
2.7.2.6
meso-Tartrate
trans-2,3-Epoxy-succinate
6.2.1.9
3.1.2.30
(R)-Methyl-malonyl-CoA
(2R)-Ethyl-malonyl-CoA
Mesaconate
1.2.2.1
cis-Aconitate
Oxaloglycolate
1.1.1.93
3.3.2.4
(S)-Malyl-CoA
2.3.3.9
5.1.99.1
5.4.99.63
Formate
2.8.3.-
(S)-Methyl-malonyl-CoA
(2S)-Methyl-succinyl-CoA
3.1.2.10
Formyl-CoA
1.3.1.7
1.2.3.4
4.1.3.24
Acetyl-CoA
6.4.1.3
L-erythro-3-Methylmalyl-CoA
1.3.8.12
Nitrogen metabolism
4.1.1.2
4.1.1.8
4.2.1.3
Ascorbate and aldarate metabolism
6.2.1.1
4.2.1.148
2.8.3.2
Oxalyl-CoA
Propionyl-CoA
Oxalate
6.2.1.8
Acetate
4.1.3.24
Mesaconyl-CoA
1.2.3.5
1.2.1.17
Glyoxylate
4.1.3.1
Isocitrate
Dihydroxy-fumarate
4.1.1.54
4.1.1.47
Succinate
Tartronate semialdehyde
1.1.99.14
4.1.3.16
Glycolate
4-Hydroxy-2-oxoglutarate
4.1.3.42
1.1.3.15
Pyruvate
1.1.1.92
1.1.1.60
5.3.1.22
1.2.1.21
O2
H2O2
1.11.1.6
4.1.3.13
3-Oxalomalate
Vitamin B6 metabolism
Oxaloacetate
Purine metabolism
1.1.1.26
2.3.3.7
D-Glycerate
1.1.1.26
1.1.1.79
3-Ethylmalate
4.1.1.40
1.1.1.79
1.1.1.29
1.1.1.29
1.1.1.81
3.1.3.18
Butanoyl-CoA
Hydroxy-pyruvate
Glycolaldehyde
Phosphoglycolate
2.3.3.12
2.7.1.165
2.7.1.31
3-Propylmalate
1.1.1.77
Pentanoyl-CoA
2-Phospho-D-glycerate
Ethane-1,2-diol
D-Ribulose 1,5-P2
4.1.1.39
Carbon fixation
2.2.1.5
3-Phospho-D-glycerate
2-Hydroxy-3-oxoadipate
Alanine, aspartate and glutamate metabolism
6.3.1.2
L-Glutamine
2-Oxoglutarate
Carbon fixation
Glycolysis / Gluconeogenesis
2-Oxoglutarate
1.4.7.1
L-Glutamate
Glycine
NH3
GDC
2.6.1.4
2.1.2.1
2.6.1.45
L-Serine
Glycine, serine and threonine metabolism

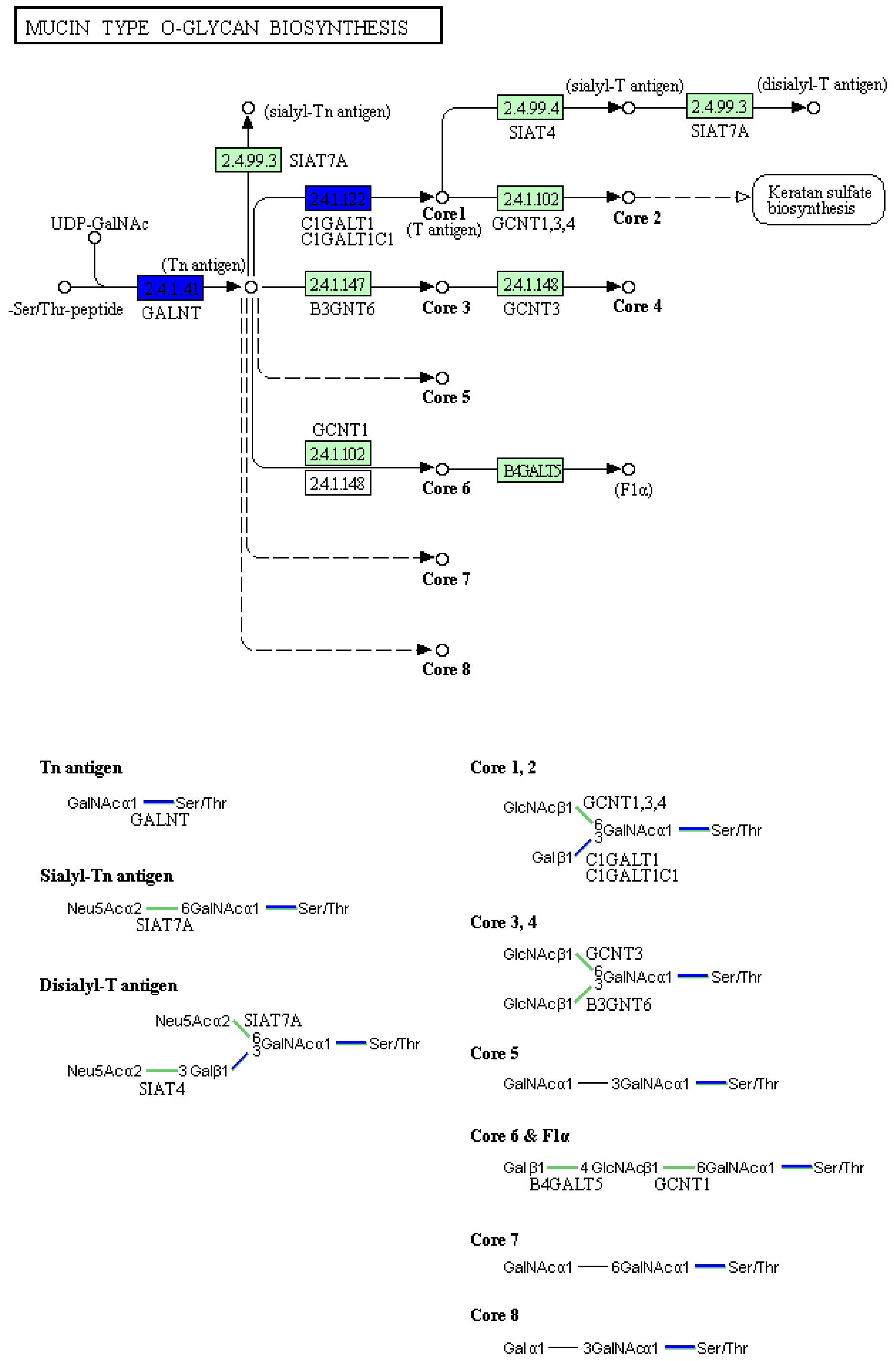
MUCIN TYPE O-GLYCAN BIOSYNTHESIS
(sialyl-Tn antigen)
2.4.99.3 SIAT7A
2.4.99.4 SIAT4
(sialyl-T antigen)
2.4.99.3 SIAT7A
(disialyl-T antigen)
UDP-GalNAc
-Ser/Thr-peptide
2.4.1.41 GALNT
(Tn antigen)
2.4.1.122 C1GALT1 C1GALT1C1
Core 1 (T antigen)
2.4.1.102 GCNT1,3,4
Core 2
Keratan sulfate biosynthesis
2.4.1.147 B3GNT6
Core 3
2.4.1.148 GCNT3
Core 4
Core 5
GCNT1
2.4.1.102
2.4.1.148
Core 6
B4GALT5
(F1α)
Core 7
Core 8
Tn antigen
GalNAcα1 Ser/Thr
GALNT
Sialyl-Tn antigen
Neu5Acα2 6GalNAcα1 Ser/Thr
SIAT7A
Disialyl-T antigen
Neu5Acα2 SIAT7A
6 3 GalNAcα1 Ser/Thr
Neu5Acα2 3 Galβ1
SIAT4
Core 1, 2
GlcNAcβ1 GCNT1,3,4
6 3 GalNAcα1 Ser/Thr
Galβ1 C1GALT1 C1GALT1C1
Core 3, 4
GlcNAcβ1 GCNT3
6 3 GalNAcα1 Ser/Thr
GlcNAcβ1 B3GNT6
Core 5
GalNAcα1 3GalNAcα1 Ser/Thr
Core 6 & F1α
Galβ1 4 GlcNAcβ1 6GalNAcα1 Ser/Thr
B4GALT5 GCNT1
Core 7
GalNAcα1 6GalNAcα1 Ser/Thr
Core 8
Galα1 3GalNAcα1 Ser/Thr

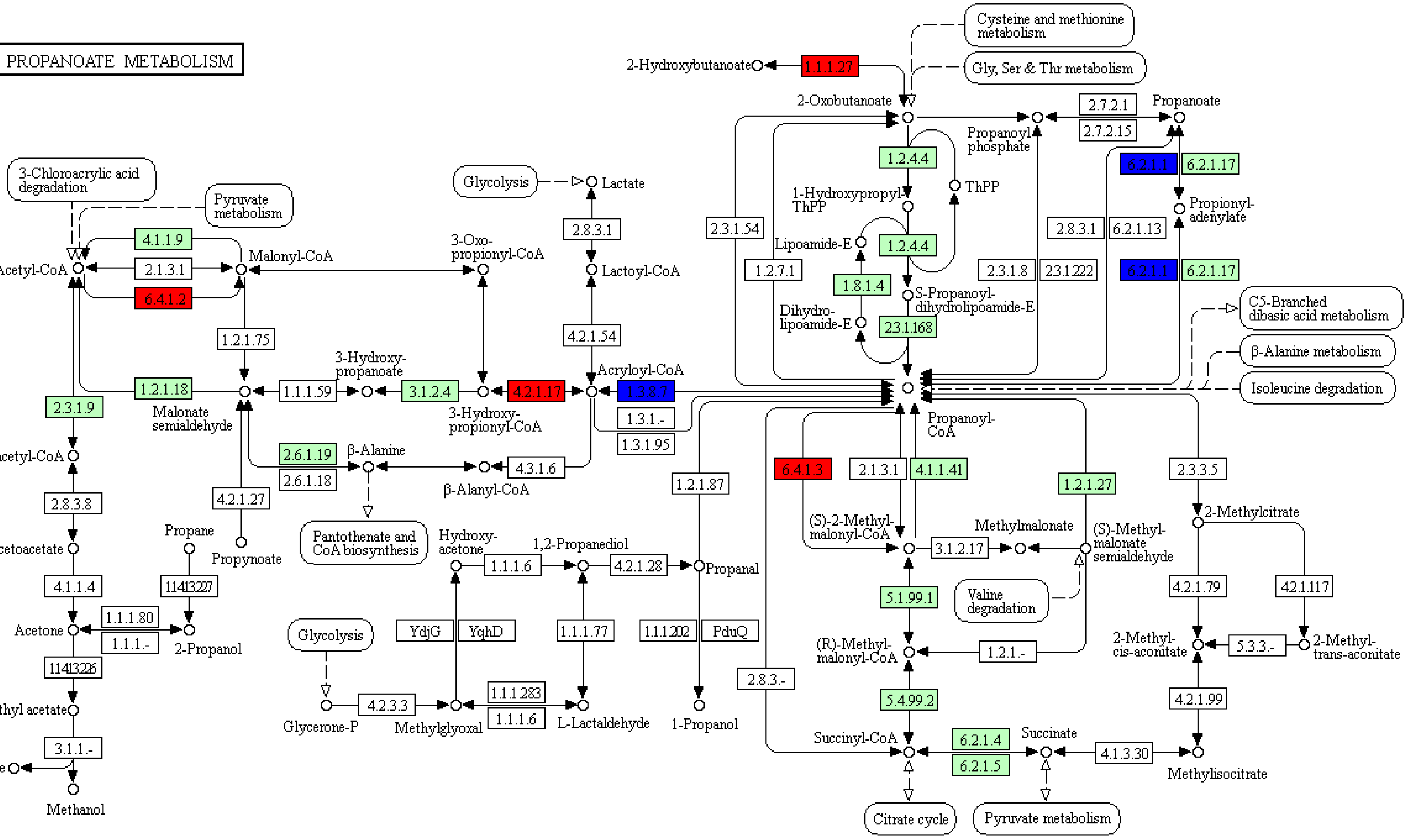

PROPANOATE METABOLISM
Cysteine and methionine metabolism
Gly, Ser & Thr metabolism
2-Hydroxybutanoate
1.1.1.27
2-Oxobutanoate
2.7.2.1
2.7.2.15
Propanoate
Propanoyl phosphate
1.2.4.4
ThPP
1-Hydroxypropyl-ThPP
6.2.1.1
6.2.1.17
Propionyl-adenylate
2.3.1.54
Lipoamide-E
1.2.4.4
2.8.3.1
6.2.1.13
1.2.7.1
1.8.1.4
2.3.1.8
2.3.1.222
S-Propanoyl-dihydrolipoamide-E
Dihydro-lipoamide-E
2.3.1.168
C5-Branched dibasic acid metabolism
β-Alanine metabolism
Isoleucine degradation
Propanoyl-CoA
3-Chloroacrylic acid degradation
Pyruvate metabolism
Glycolysis
Lactate
2.8.3.1
Lactoyl-CoA
4.2.1.54
Acryloyl-CoA
3-Oxo-propionyl-CoA
4.1.1.9
2.1.3.1
6.4.1.2
Malonyl-CoA
Acetyl-CoA
1.2.1.75
3-Hydroxy-propanoate
1.2.1.18
1.1.1.59
3.1.2.4
4.2.1.17
1.3.8.7
1.3.1.-
1.3.1.95
2.3.1.9
Malonate semialdehyde
3-Hydroxy-propionyl-CoA
Acetoacetyl-CoA
2.6.1.19
2.6.1.18
β-Alanine
4.3.1.6
β-Alanyl-CoA
2.8.3.8
4.2.1.27
Propane
Propynoate
Pantothenate and CoA biosynthesis
Acetoacetate
Hydroxy-acetone
1,2-Propanediol
1.1.1.6
4.2.1.28
Propanal
1.2.1.87
6.4.1.3
2.1.3.1
4.1.1.41
1.2.1.27
2.3.3.5
2-Methylcitrate
(S)-2-Methyl-malonyl-CoA
Methylmalonate
3.1.2.17
(S)-Methyl-malonate semialdehyde
4.1.1.4
1.14.13.227
1.1.1.80
1.1.1.-
Acetone
2-Propanol
1.14.13.226
Glycolysis
YdjG
YqhD
1.1.1.77
1.1.1.202
PduQ
5.1.99.1
Valine degradation
4.2.1.79
4.2.1.117
(R)-Methyl-malonyl-CoA
1.2.1.-
2-Methyl-cis-aconitate
5.3.3.-
2-Methyl-trans-aconitate
Methyl acetate
1.1.1.283
2.8.3.-
4.2.3.3
Glycerone-P
Methylglyoxal
1.1.1.6
L-Lactaldehyde
1-Propanol
5.4.99.2
4.2.1.99
Succinyl-CoA
6.2.1.4
6.2.1.5
Succinate
4.1.3.30
Methylisocitrate
3.1.1.-
Methanol
Citrate cycle
Pyruvate metabolism

PROXIMAL TUBULE BICARBONATE RECLAMATION

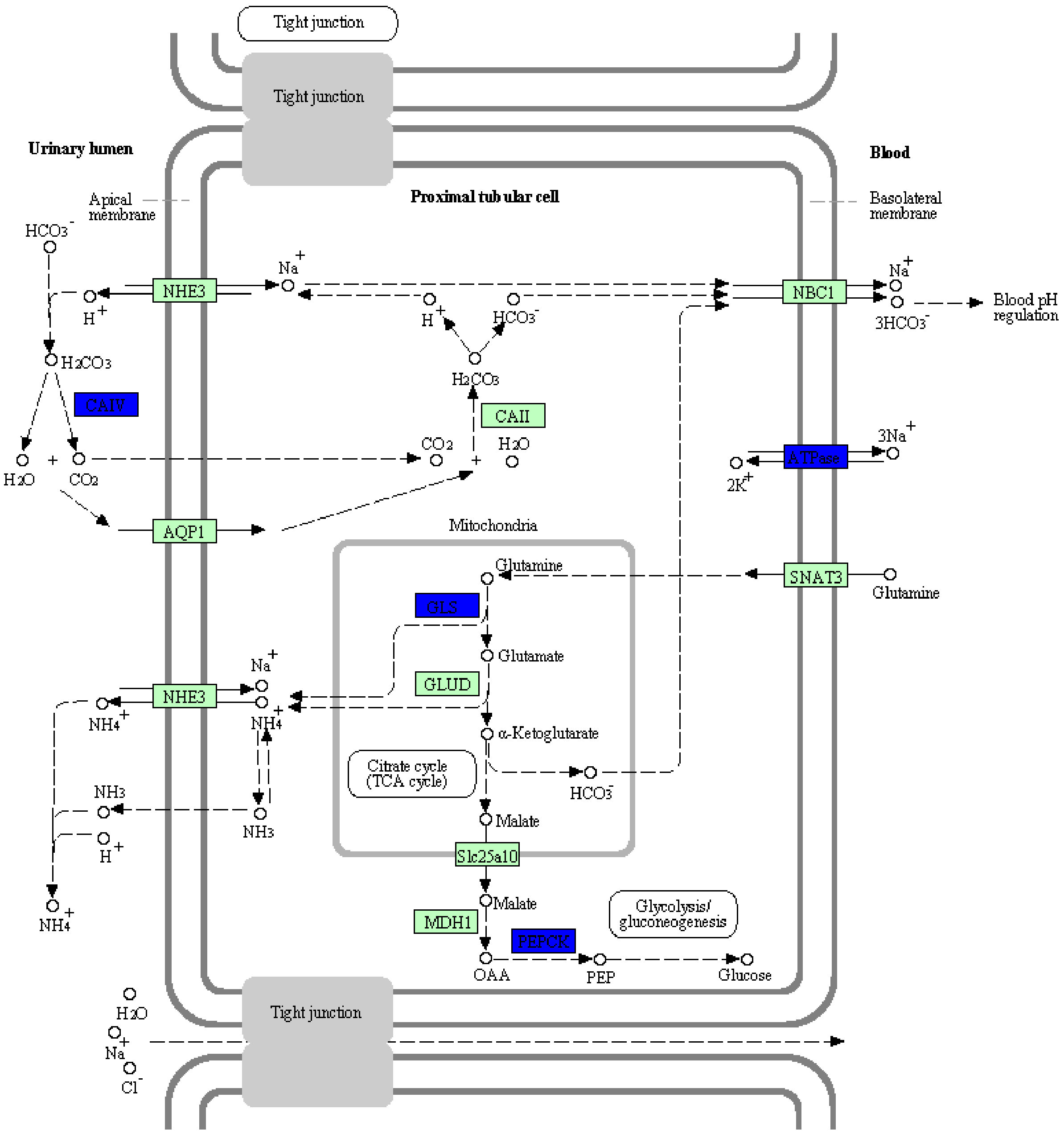

Supplement: Supplementary file 13 [file Image_1.pdf]
